# Where Are the Gaps in Diabetes Care? An Evidence Gap Mapping of the Diabetes Patient Journey in Indonesia

**DOI:** 10.1111/tmi.70100

**Published:** 2026-02-04

**Authors:** Rachmadianti S. Hanifa, Sudewi M. Khoirunnisa, Rizka Maulida, Firas F. Alkaff, Maarten J. Postma, Jurjen van der Schans

**Affiliations:** ^1^ Department of Health Sciences University Medical Center Groningen Groningen the Netherlands; ^2^ Department of Pharmacy Institut Teknologi Sumatera Lampung Selatan Indonesia; ^3^ Department of Epidemiology, Faculty of Public Health Universitas Indonesia Depok Indonesia; ^4^ Division of Pharmacology and Therapy, Department of Anatomy, Histology, and Pharmacology, Faculty of Medicine Universitas Airlangga Surabaya Indonesia; ^5^ Division of Nephrology, Department of Internal Medicine, University of Groningen University Medical Center Groningen Groningen the Netherlands; ^6^ Department of Economics, Econometrics, & Finance University of Groningen, Faculty of Economics & Business Groningen the Netherlands; ^7^ Center of Excellence for Pharmaceutical Care Innovation Universitas Padjadjaran Bandung Indonesia

**Keywords:** delivery of health care, diabetes mellitus, evidence gaps, patient journey, patient navigation

## Abstract

**Objective:**

Understanding the diabetes patient journey, from awareness, screening, diagnosis, treatment, adherence and control, is crucial for improving outcomes. However, in many low‐ and middle‐income countries, including Indonesia, data across this continuum of care remain limited due to limited health information exchange capacity. This study aimed to map and estimate the prevalence of true diabetes and key touchpoints along the patient journey to identify gaps in diabetes management in Indonesia.

**Methods:**

We applied an evidence gap mapping approach by systematically searching relevant literature published between 1 January 2014, and 27 March 2025, in PubMed, Web of Science, Scopus and Embase, complemented by unstructured searches of government websites. Evidence was visualised using a chord diagram, bar chart and heatmaps. Random‐effects meta‐analyses were used to estimate pooled prevalence, stratified by study setting and sample representativeness relative to the national population.

**Results:**

Among 94 records, none fully applied diabetes patient journey frameworks and most of the evidence was assessed as low quality. Screening was the least studied touchpoint and awareness missed nationally representative data. Pooled prevalence estimates were: true diabetes 13% (95% confidence interval (CI): 9%–17%), awareness 71% (95% CI: 54%–85%), screening 19% (95% CI: 0%–55%), diagnosis 15% (95% CI: 6%–27%), treatment 89% (95% CI: 80%–95%), adherence 59% (95% CI: 49%–69%) and control 31% (95% CI: 25%–36%). Prevalence rates were generally lower in studies conducted in the general population using nationally representative samples. Pooled prevalence across touchpoints and subgroups decreased after excluding low‐quality studies, although heterogeneity within subgroups remained high.

**Conclusion:**

Evidence gap mapping offers a practical approach to generating local patient journey data in countries with limited health information exchange capacity. Specifically for Indonesia, a comprehensive consideration of the diabetes patient journey is lacking. Barriers exist across all touchpoints, particularly in the general population.

## 1 Introduction

Diabetes is a major global health challenge, affecting an estimated 589 million adults worldwide in 2024, with approximately 80% of cases occurring in low‐ and middle‐income countries (LMICs) [1]. The global burden of diabetes is further reflected by the rising diabetes‐related mortality from 12.4 to 20.5 deaths per 100,000 population in recent decades, disproportionately affecting individuals aged 30–69 years [2].

In Indonesia, diabetes ranks among the top five contributors to morbidity, with the national prevalence rising from 10.9% in 2018 to 11.7% in 2023 [3, 4]. Recognising this growing burden, the Indonesian government has established an Integrated NCD care system to ensure the continuity of diabetes care since 2013. It includes cascade care from prevention at the community level to diagnosis and initial treatment at the primary health care facilities and advanced care at the secondary and tertiary level for managing complications [5]. This approach aligns with the World Health Organization's Package of Essential Noncommunicable (PEN) Disease Interventions [6].

However, despite over a decade of this policy effort, diabetes prevalence in the country continues to rise and mortality from diabetes‐related complications is projected to double between 2020 and 2045 under the current health system landscape [5, 7]. This trend suggests a possible gap between policy design and its effective implementation, highlighting the need to better understand where patients drop out along the diabetes care continuum.

Generally, the overall care pathway for NCDs includes six key touchpoints: awareness, screening, diagnosis, treatment, adherence and control [8, 9]. This framework emphasises that the pathway for NCDs often begins before individuals engage with the healthcare system. It typically starts when people become aware of the disease and its risk factors, prompting them to utilise healthcare services [9]. This clinical pathway can also be viewed as the journey of diabetes patients across the entire continuum of care. Mapping this journey is particularly relevant for identifying bottlenecks in the current NCD care system and providing evidence‐based insights for improving care [8, 9, 10].

However, NCD patient journey data, particularly for diabetes, is sparse in LMICs, which may be caused by limited capacity in health information system infrastructure [8, 11]. Likewise, systematic reviews of PEN intervention implementation in other LMICs constantly cite an inadequate electronic registration system as a barrier to tracking the progress [12, 13]. Additionally, relying on international data to understand local patient journey patterns may misguide local policy development, given the significant differences in care structures, governance and patient behaviours.

In light of this, this study aims to fill the evidence gap on the diabetes patient journey data in Indonesia by synthesising available evidence across the six key patient journey touchpoints. Specifically, it seeks to answer the following research questions: What is the estimated prevalence of true diabetes and its key patient journey touchpoints (i.e., awareness, screening, diagnosis, treatment, adherence and control) in Indonesia? The findings could help policymakers identify bottlenecks in diabetes management and provide actionable solutions to strengthen the diabetes care continuum in Indonesia.

## 2 Methodology

### 2.1 Study Design

This study employed an evidence gap mapping (EGM) methodology, guided by the MAPS framework (Mapping the Patient Journey Towards Actionable Beyond‐the‐Pills Solutions), to identify knowledge gaps and synthesise prevalence data across key touchpoints in the diabetes patient journey in LMICs [9]. In general, EGM aims to systematically organise existing literature within a structured framework to improve the accessibility of evidence [14, 15]. The EGM process began with a systematic search of relevant databases and grey literature. The extracted data were then synthesised and visualised to identify gaps and estimate the prevalence of true diabetes, as well as its patient journey touchpoints.

The definition of true diabetes prevalence and its patient journey touchpoints are explained in Table 1. True diabetes was included to distinguish biochemically diabetes cases from the diagnosis touchpoint. The study protocol is registered at https://osf.io/under the 10.17605/OSF.IO/52XGR identifier.

**TABLE 1:** Definitions of True Diabetes and Its Patient Journey Touchpoints.

Terms	Definition
True diabetes	Based on the latest Indonesian Diabetes Management Guidelines [16], which include: HbA1c value of ≥ 6.5% or Fasting plasma glucose value of ≥ 126 mg/dL or 2‐h plasma glucose of ≥ 200 mg/dL during an oral glucose tolerance test or Random plasma glucose of ≥ 200 mg/dL followed by classic symptoms of hyperglycemia or hyperglycemic crisis
Diabetes patient journey touchpoints	
Awareness	Self‐reported knowledge about diabetes symptoms, risk factors and complications.
Screening	Self‐reported blood glucose measurement or diabetes screening using any other validated diabetes screening tools.
Diagnosis	Self‐reported diagnosis of diabetes by a health care professional.
Treatment	Self‐reported use of any glucose‐lowering medication among the diagnosed diabetes population
Adherence	Self‐reported adherence with the prescribed glucose‐lowering medication among the treated diabetes population
Control	The achievement of target therapy among treated diabetes populations based on the latest Indonesian Diabetes Management Guidelines [16], which includes: HbA1c value of < 7% or Capillary pre‐prandial blood glucose measurement value between 80–130 mg/dL or 2‐h plasma glucose value of < 180 mg/dL

### 2.2 Search Strategies

A structured search of the Web of Science, Scopus, Embase and PubMed databases was conducted to identify articles published between January 1, 2014, and March 27, 2025. This timeframe was selected to minimise bias in patient journey prevalence, as the implementation of the National Health Insurance Scheme in January 2014 may have influenced the diabetes patient journey [17]. The search focused on diabetes and its patient journey touchpoints. Detailed search strategies are provided in Supporting Information A. An unstructured search on the government website, along with citation searching from the identified systematic review, was also performed.

### 2.3 Eligibility Criteria for Included Studies

Eligible studies were selected based on the following criteria:

1. Studies involving adults (≥ 18 years) reporting the prevalence of true diabetes or its patient journey touchpoints.
2. Peer‐reviewed observational studies (cross‐sectional, case–control or cohort) or intervention studies using data collected after 31 December 2013.
3. Studies conducted among the Indonesian population.

Studies were excluded if they focused solely on specific populations (e.g., pregnant women, patients with comorbidities), had unclear or inconsistent definitions of patient journey touchpoints as defined in this study or had inconsistent denominators for calculating prevalence. Studies using similar databases that covered the same touchpoints were also excluded and only the studies with the largest sample sizes were retained for the final analysis. For the awareness touchpoints, studies were excluded only if ‘knowledge’ did not clearly pertain to symptoms, risk factors or complications.

### 2.4 Screening of Studies

RSH conducted a comprehensive search of studies using both structured and unstructured strategies, removing duplicates and excluding non‐human studies. RSH and SMK or FFA independently screened the articles based on titles and abstracts. RSH performed full‐text screening against the predetermined inclusion and exclusion criteria while RM or FFA verified these decisions. Any reviewer disagreements during the screening process were resolved through discussion, with input from JvdS.

### 2.5 Data Extraction and Risk of Bias Assessment

RSH conducted the initial data extraction and risk of bias assessment, which was verified by SMK, RM or FFA. Data were extracted into a standardised grid in Microsoft Excel 365, capturing information on study design, settings, sample size, population characteristics, quantitative data for patient journey touchpoints and their measurement method, along with the risk of bias assessment. For longitudinal studies, only baseline data were included. For case–control studies, data were extracted exclusively from the control group or groups without other comorbidities. The risk of bias was assessed using the Joanna Briggs Institute Critical Appraisal Checklist for Studies Reporting Prevalence Data, assuming all studies are cross‐sectional [18]. Each item was scored as 1 for ‘Yes or Not Applicable’, 0 for ‘Unclear or No’, with total scores ranging from 0 to 9, categorised into low (0–3), medium (4–6) or high quality (7–9).

### 2.6 Data Analysis and Visualisation

A chord diagram was used to illustrate the extent to which individual studies assessed multiple patient journey touchpoints simultaneously. A bar graph was used to present the number of touchpoints assessed by the included studies, along with their quality, study setting and sample representativeness relative to the national population. The heatmap illustrated the geographical distribution of sample coverage. A random‐effects meta‐analysis was conducted to estimate the pooled prevalence of true diabetes and each patient journey touchpoint. Clopper‐Pearson confidence intervals were calculated for each observation and arcsine transformations of proportions were applied before the meta‐analysis [19, 20]. Given the expected high heterogeneity, we used the Restricted Maximum Likelihood to estimate between‐study variance and the Hartung‐Knapp‐Sidik‐Jonkman method to calculate the confidence interval around the pooled prevalence [21]. Prediction intervals were computed to further assess heterogeneity [22, 23].

Subgroup analyses were performed based on the representativeness of the study sample relative to the national population and the study's settings. To account for methodological variation, post hoc subgroup analyses were also conducted for touchpoints assessed with different measurement tools or definitions. Studies were excluded from subgroup analysis if data on the geographical spread of the samples were unclear. According to the MAPS framework, studies with sample sizes of at least 500 can be classified as nationally representative [9]. Considering Indonesia's vast geographical diversity, we refined this definition as follows:

1. Nationally representative sample: participants from at least two geographical regions in Indonesia (Java, Kalimantan, Maluku, Nusa Tenggara, Papua, Sulawesi or Sumatra).
2. Regionally representative samples: Participants from at least two different provinces within the same geographical region.
3. Provincially representative samples: participants from a single province.

Post hoc sensitivity analysis by excluding all low‐quality evidence was also performed to assess the robustness of the estimates. All analyses were conducted using R (version 4.4.1, 2024‐06‐14 UCRT) with the metaprop package and data visualisation was constructed using ggplot2, leaflet and igraph packages [24, 25, 26].

## 3 Results

### 3.1 Review of Retrieved Records

**FIGURE 1:**
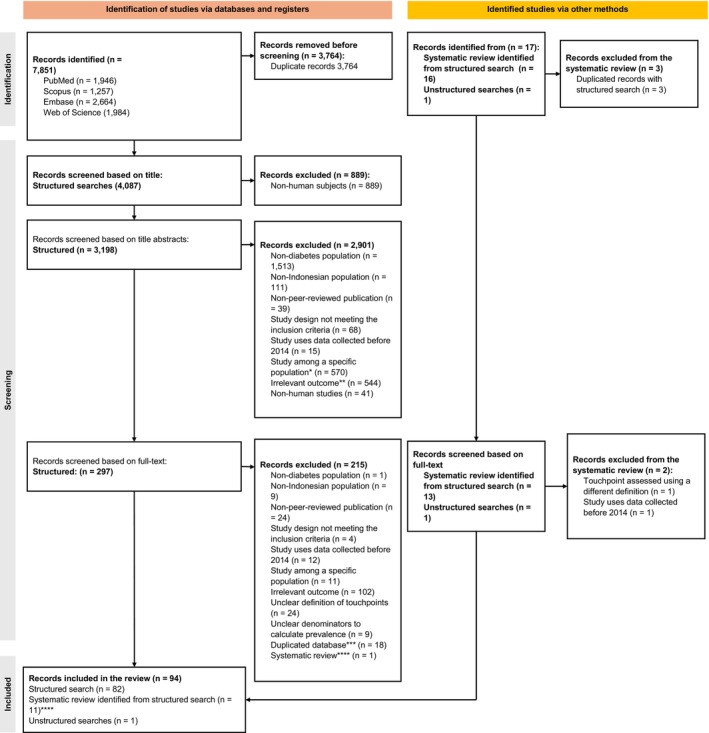
Flowchart describing the inclusion of records into the final analysis. *Conducted only in specific groups, e.g., people with specific comorbidities, smokers, pregnant women and those < 18 years old. **No diabetes‐related touchpoints were reported. ***Database using similar data source. ****Relevant systematic review with included studies appraised in the unstructured search.

A total of 7851 studies were identified through the structured search. After removing duplicates and non‐human studies, 3198 studies remained for title and abstract screening. Of these, 297 were assessed in full text, with 215 excluded based on the eligibility criteria. Among the excluded studies, 18 were removed because they addressed the same diabetes patient journey touchpoints using identical databases (Supporting Information B). From the structured search, we also identified a systematic review comprising 16 articles, which were subsequently assessed. Eleven of these articles met our inclusion criteria and were incorporated into the analysis. Additionally, one record from a government website was added, resulting in a total of 94 records included in the final analysis (Figure 1). A detailed description of the included records is further explained in Supporting Information C.

### 3.2 Evidence Gap Mapping

Among the 94 included records, six assessed only true diabetes prevalence, while the remaining 88 focused on one or more diabetes patient journey touchpoints.

**FIGURE 2:**
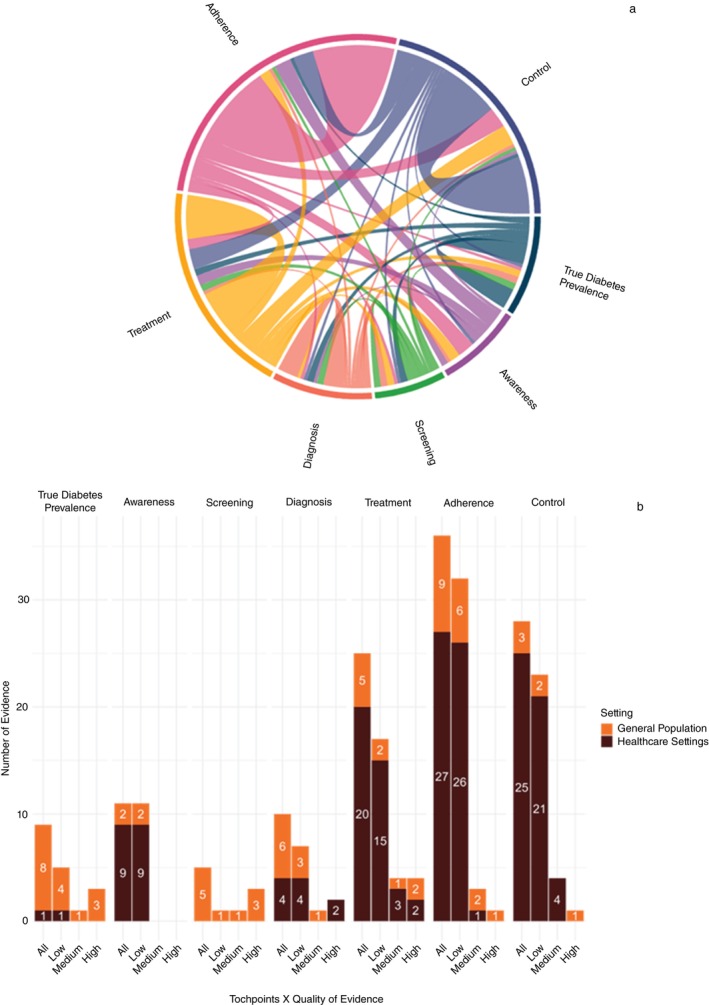
(a) Concurrence of diabetes touchpoints within the individual records; (b) Number of evidence by touchpoints type, settings and quality.

In total, 115 touchpoints and nine true diabetes prevalence data points were identified. However, none of these records assessed both true diabetes prevalence and all six patient journey touchpoints simultaneously. Most studies evaluated only one aspect, either true diabetes prevalence or a single touchpoint (Figure 2a). Only a small number of records assessed multiple touchpoints: 21 records assessed two touchpoints, one assessed three and one assessed five (Supporting Information C).

Among the touchpoints, diabetes screening was the least studied, appearing in only five of the 94 records, whereas adherence and control were the most frequently investigated (Figure 2b). Additionally, most data came from studies conducted in healthcare settings, except for true diabetes prevalence and screening, which were predominantly assessed in general population settings. According to the risk of bias assessment, the majority of data sources were rated as low quality, except for screening data, which tended to be of higher quality. All evidence on diabetes awareness came from low‐quality studies, whereas nearly all high‐quality data sources originated from general population studies using nationally representative samples (Figure 2b and Table D2 in Supporting Information D).

**FIGURE 3:**
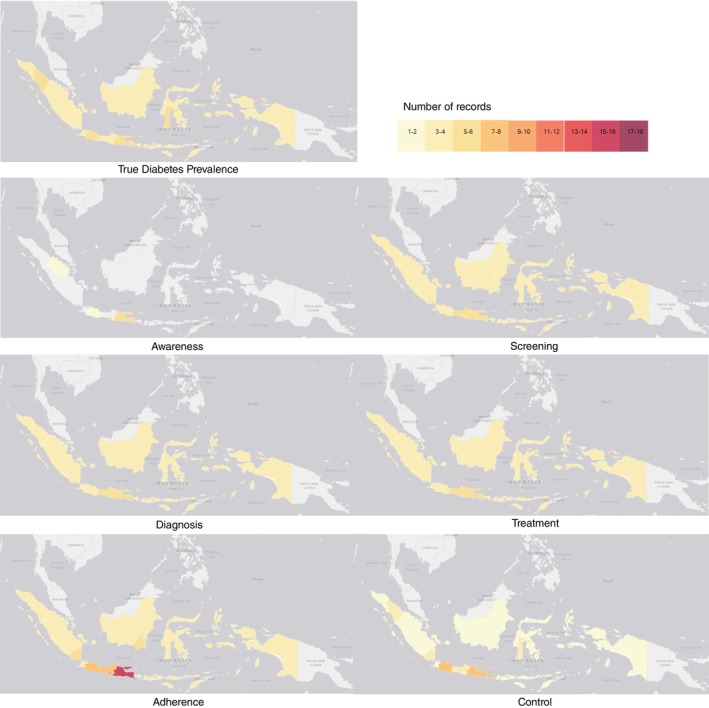
Heatmap of evidence mapping by geographical sample coverage. Four studies were excluded from the graph: one assessing diabetes treatment and adherence, one assessing treatment and control and two assessing adherence due to unclear geographical settings.

Figure 3 maps the number of studies based on their geographical sample coverage. While most touchpoints were represented by studies from various regions of Indonesia, diabetes awareness was an exception, with evidence limited to participants from East Java, the Special Region of Yogyakarta, West Java and Riau.

### 3.3 Pooled Estimates of Diabetes and Touchpoints Prevalence

**FIGURE 4:**
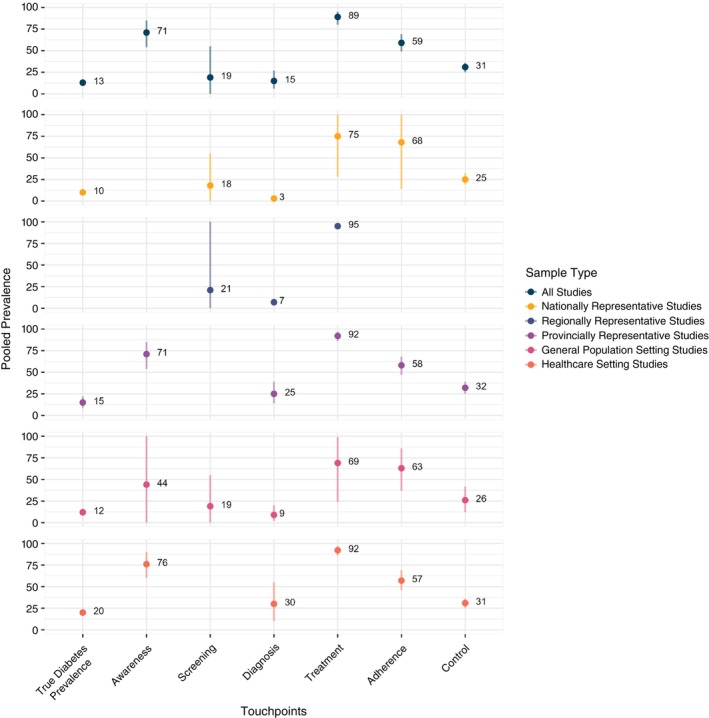
Pooled prevalence of diabetes and its touchpoints by sample type. *Point represents the pooled prevalence value, while the upper and lower line represents its confidence interval. **One awareness and three adherence touchpoints data were not included in the subgroup analysis based on sample coverage type due to unclear geographical settings.

Figure 4 illustrates the pooled prevalence of true diabetes and its patient journey touchpoints, categorised by the type of sample and study setting. The overall pooled prevalence of true diabetes was 13% (95% confidence interval (CI): 9%–17%, *I* ^2^ = 96%, *p* < 0.0001), while the prevalence of diabetes awareness was 71% (95% CI: 54%–85%, *I* ^2^ = 97.7%, *p* < 0.0001). The prevalence of diabetes screening was 19% (95% CI: 0%–55%, *I* ^2^ = 100%, *p* < 0.0001), while the prevalence of diagnosed diabetes was 15% (95% CI: 6%–27%, *I* ^2^ = 99.4%, *p* < 0.0001). Additionally, 89% (95% CI: 80%–95%, *I* ^2^ = 100%, *p* < 0.0001) of diagnosed diabetes patients received treatment. However, treatment adherence was only 59% (95% CI: 49%–69%, *I* ^2^ = 99.1%, *p* < 0.0001) and the prevalence of diabetes control was 33% (95% CI: 31%–34%, *I* ^2^ = 95.7%, *p* < 0.0001). When low‐quality evidence was excluded from the sensitivity analysis, the pooled prevalence of true diabetes, screening, diagnosis and treatment decreased to 10%, 11%, 4% and 78%, respectively. In contrast, adherence increased to 68% and diabetes control remained similar at 32%. Sensitivity analysis was not performed for awareness, as all evidence was rated as low‐quality (Supporting Information F).

The heterogeneity analysis revealed substantial variability across the included studies, likely driven by differences in sample types and study settings. Notably, the pooled prevalence of diagnosed, treated and controlled diabetes was significantly lower in studies using nationally representative samples than in those using provincially representative samples, even after removing low‐quality studies. On the contrary, the pooled prevalence of true diabetes, awareness and diagnosed diabetes was significantly higher in studies conducted in healthcare settings than in those based on general population samples. Specifically for diagnosed diabetes, this finding remains unchanged after removing low‐quality studies. No significant differences were observed in the subgroup analyses of the pooled prevalence of diabetes treatment, adherence and diabetes control based on settings. However, after removing the low‐quality studies, the pooled prevalence of diabetes control became significantly higher in healthcare‐based studies than in general population samples, while the treatment and adherence touchpoints remained the same.

Despite these subgroup patterns, considerable variability persisted across touchpoints even within subgroups, suggesting that additional factors beyond sample type and setting may contribute to the observed heterogeneity.

For awareness and adherence, differences in measurement tools and cut‐off points may also explain heterogeneity: studies using instruments other than DKQ‐24 or applying higher DKQ‐24 cut‐offs reported markedly lower awareness, while those using single‐item questions or tools other than MMAS, ARMS or MARS reported higher adherence. For screening, self‐reported risk‐based diabetes screening showed a lower prevalence estimate than self‐reported blood glucose testing. Finally, despite the high pooled prevalence of glucose‐lowering medication use (~90%), applying a stricter threshold of ≥ 23 prescription days revealed a substantially lower prevalence of 32% (See Supporting Information E for subgroup analysis).

## 4 Discussion

### 4.1 Main Findings

This review mapped, synthesised and quantified the available evidence on key touchpoints of the diabetes patient journey in Indonesia, including awareness, screening, diagnosis, treatment, adherence and control, as well as the true diabetes prevalence. First, none of the 94 eligible records comprehensively captured all patient journey touchpoints. Similar patterns have been reported in MAPS framework studies on diabetes neuropathy in the Philippines and Saudi Arabia, where evidence focused on single touchpoints rather than the entire continuum of care [27, 28]. Second, nationally representative and high‐quality evidence on diabetes awareness, a crucial step for engaging patients in timely care, is lacking. This reflects a broader issue in many LMICs, where studies on diabetes knowledge are limited and public awareness programs remain a low policy priority [29, 30]. Third, pooled estimates varied notably by sample representativeness, even among medium to high‐quality data, likely reflecting geographic disparities in disease burden and healthcare access, as shown in the previous studies [31, 32, 33]. Lastly, while our findings are specific to Indonesia, they reflect a wider challenge faced by LMICs with limited health information exchange systems [11]. In this context, evidence gap mapping offers a practical and transferable approach to identifying critical data gaps, clarifying service delivery bottlenecks and informing more targeted strategies to strengthen NCD care across the continuum [8, 9, 15, 34].

### 4.2 Gaps Along Patient Journey Touchpoints

Our overall estimate of true diabetes prevalence was 13% (10% in medium to high‐quality studies only), aligning closely with the age‐standardised prevalence reported by the International Diabetes Federation (IDF) of 11.3% [1]. Notably, prevalence estimates across all touchpoints were generally lower in the general population‐based studies compared to healthcare‐based studies. This discrepancy may reflect access barriers, suggesting that the implementation of national health insurance and the Integrated NCD Care program, initiated in 2014, may not yet have fully achieved the intended improvements in healthcare access [5, 17].

Pooled estimate of good diabetes awareness was moderate at 71%, with significantly lower rates in general population studies (44%) compared to healthcare settings (76%). Heterogeneity remained high even within the subgroup, likely due to differing questionnaires used and the cut‐off points. Nevertheless, even within studies using comparable questionnaires and cut‐off thresholds, awareness remained consistently lower in general population (31%) samples than in healthcare settings (62%) [35, 36]. These suggest a link between healthcare access and disease awareness, as supported by a study showing improved diabetes awareness among participants in community‐based health intervention as part of the Integrated NCDs care program [37].

The moderate awareness level is further reflected in the low estimated screening prevalence of 19% (11% in medium to high‐quality studies only), supporting evidence that poor awareness can hinder screening uptake [38]. The persistent heterogeneity within subgroups likely reflects variation in screening tools and data collection periods. For example, screening rates increased from 11% in a 2014/2015 national study [39], to 34% in 2023 [4], potentially reflecting the impact of community‐based screening initiatives introduced in early 2014 [37]. Additionally, the pooled prevalence of risk score‐based diabetes screening was low at 4%. In Indonesia, such screening is typically delivered through a digital application, where its uptake may be influenced by limited health literacy and low perceived personal risk, despite the potential of the digital platform to reach large segments of the population [40].

The estimated pooled prevalence of diagnosed diabetes was 15% (4% among medium to high‐quality studies only). Nationally representative studies reported a significantly lower prevalence (3%) compared to provincially representative studies (25% or 10% among medium to high‐quality studies only) and general population studies reported a significantly lower prevalence (9% or 4% in medium to high‐quality studies only) compared to those based in healthcare settings (30%). These differences likely reflect a substantial burden of undiagnosed diabetes in the general population, exacerbated by inequities in healthcare access. According to the IDF, 73.2% of diabetes cases in Indonesia remain undiagnosed, well above the Western Pacific regional average of 50% [1]. This gap may result from limited diagnostic capacity, particularly in low‐resource settings [31]. Higher diagnosis rates in healthcare‐based studies may also reflect the increased likelihood of identifying diabetes as a comorbidity among patients seeking care for other conditions [41].

Pooled treatment prevalence was high at 89% (78% in medium to high‐quality studies only). Nationally representative studies reported significantly lower treatment rates than provincially representative ones (75% vs. 92% for overall studies; 75% vs. 78% in medium to high‐quality studies only). This variation may reflect regional differences in health system capacity, periods of data collection and the definition of treatments. For example, a 2014/2015 study from Western Indonesia reported only 13% treatment coverage [42], while the 2023 national survey reported 92% [4], suggesting a positive impact of JKN and the Integrated NCD Care program on improving access to diabetes treatment [5, 17]. However, a 2016 study based on insurance claims data applied a strict 23‐day medication prescription threshold and found a much lower treatment prevalence of 32%, suggesting that many patients may not receive care aligned with clinical guidelines [43].

Treatment adherence was estimated at 59% (68% in medium to high‐quality studies only), with substantial heterogeneity likely influenced by differences in measurement methods. Single‐item or unclear self‐report questions yielded a higher pooled adherence prevalence of 70%–75% than multi‐item questionnaires, such as MMAS, ARMS and MARS (29%–62%), likely reflecting overestimation from single‐item questions, which cannot adequately account for social desirability bias [44]. Beyond measurement issues, adherence is shaped by multiple factors, including socioeconomic status, healthcare accessibility and patient‐related barriers [45]. One included study found that the use of complementary and alternative medicine, a common practice in Indonesia [46], was significantly associated with lower adherence to glucose‐lowering medications [47]. This may imply that moderate diabetes awareness, as noted earlier, may also contribute to the suboptimal adherence observed in this review.

Finally, pooled diabetes control prevalence, which was all based on HbA1c < 7% measurement, was estimated at 31% (32% when excluding low‐quality studies), with lower rates in the general population than in healthcare settings (26% vs. 31% in overall studies; 19% vs. 39% in medium to high‐quality studies only). These findings may reflect the critical role of sustained engagement with healthcare systems in achieving glycemic control. Even with higher adherence estimates in medium‐ to high‐quality studies, poor control likely reflects additional barriers, including ineffective referral pathways, inadequate provider competency and suboptimal access to guideline‐recommended treatments, which were reported in the included studies and echoed in our review [48, 49]. Moreover, poor adherence to behavioural changes, though not captured in this review, is known to remain low among treated individuals and may further contribute to suboptimal glycemic control [50].

### 4.3 Implications for Policy and Practice

Our findings highlight critical bottlenecks along the diabetes care continuum in Indonesia. While community‐based health interventions under the current integrated NCD care approach have improved diabetes awareness and screening uptake, these gains have not led to better outcomes in diagnosis, treatment adherence and diabetes control. These gaps may stem from uneven intervention uptake, especially among men and younger adults and from modest awareness levels that may not sufficiently increase perceived risk to drive care‐seeking, as noted in previous studies [37, 40, 51]. Although treatment coverage appears high, access to guideline‐recommended therapies remains low, likely due to the unintended consequences of the current health financing model [52]. This shortfall may contribute to high rates of uncontrolled diabetes. Geographic disparities in health system capacity, as identified in prior research, may also exacerbate these challenges [33].

Based on the above‐mentioned gaps, we proposed several actionable strategies to strengthen the diabetes patient journey in Indonesia. First, standardised data collection at both national and subnational levels is crucial for informed policymaking. Despite recent efforts to integrate health data through digital transformation, a major gap remains in diabetes awareness data, with no institution currently responsible for its collection [53]. Given the critical role of health literacy in enabling healthcare access and our finding that moderate awareness may impede progression along the care continuum, nationally representative studies are needed to assess diabetes knowledge among the general population, using a questionnaire such as DKQ‐24, which has been validated in Indonesia [54, 55]. Further, research can also explore how improving diabetes knowledge influences perceived risk and care‐seeking behaviour.

Second, policymakers and healthcare practitioners should actively involve patients when shaping diabetes policy or interventions. We highlight that the diabetes patient journey in Indonesia is influenced by the accessibility of healthcare services, many of which are rooted in sociodemographic inequities. The World Health Organization highlights the value of patient engagement in improving care quality and system responsiveness [56]. Evidence shows that co‐creation of interventions with patients and public participation in health decision‐making is associated with better service accessibility and quality [57, 58].

Third, health policymakers should formally recognise community health workers (CHWs) within the healthcare system and expand training opportunities for general practitioners. Although CHWs play a key role in the integrated NCD care policy, they still lack formal status, resulting in inadequate training and institutional support [37, 59]. In parallel, the policy also relies on primary healthcare providers, yet many of them have insufficient clinical knowledge of diabetes, underscoring the need for improved pre‐service and on‐the‐job training in diabetes care [60, 61].

Lastly, reforming the health financing system is essential, particularly by optimising the budget allocation for prevention efforts and addressing the unintended consequences of the current curative and rehabilitative financing model. Currently, only about 17% of total health expenditure is allocated to health promotion and disease prevention, which is further strained by a shortage of trained health promoters, unclear activity guidelines, fragmented funding sources and inflexible budget usage [62, 63]. Although the existing curative and rehabilitative financing system aligns with the integrated NCD care model, some unintended effects may hinder treatment access, adherence and diabetes control. Under the INA‐CBGs scheme, hospitals provide only a 7‐day supply of diabetes medication, with the remaining doses requiring a more complex non‐CBGs reimbursement process. This may force patients to make frequent visits or seek medications externally, potentially disrupting adherence [43]. Once stabilised, patients return to primary care under the Chronic Disease Management Program, which uses a pay‐for‐performance financing model tied to glycemic control. Since glycemic control highly depends on the patient factors beyond provider control, this model may unintentionally disincentivize providers from treating patients with poorer baseline characteristics, potentially limiting equity [52].

### 4.4 Strengths and Limitations

This review has several strengths. First, we used an evidence gap mapping methodology that followed a semi‐systematic review approach, combining elements of both systematic and narrative reviews. This allowed analysis to maximise the coverage of the included studies while maintaining flexibility in synthesis. Second, we imposed no language restrictions, minimising potential publication bias. However, several limitations should also be acknowledged. First, we excluded studies conducted on specific populations, which may have led to an underestimation of the overall results. However, this was a reasonable approach, as the patient journey can differ significantly between specific populations and including these studies could have introduced more heterogeneity to the findings. Second, most of the included studies are of low to moderate quality, which could reduce the precision of the pooled estimates. Nevertheless, subgroup analyses showed that nationally representative studies—most of which were high quality—produced lower prevalence estimates. Similarly, sensitivity analyses excluding low‐quality studies also yielded lower estimates, except for adherence. Lastly, our findings are generalizable only to the Indonesian context or potentially to other LMICs with similar healthcare systems, as the patient journey is closely shaped by local health system structures. Nevertheless, we argue that the evidence gap mapping approach used in this review provides a transferable model for other LMICs aiming to generate country‐level data on NCD care pathways.

## 5 Conclusion

This review reveals significant data gaps in the diabetes patient journey in Indonesia, particularly regarding awareness and screening touchpoints. Despite efforts to address the high diabetes burden through the existing integrated NCD programs and national health insurance, substantial gaps persist at all touchpoints and the pooled prevalence at each stage is even lower when limited to medium to high‐quality studies. Addressing these issues requires further research, patient involvement in decision‐making, improved quality of primary healthcare workers and recognition of community health workers, along with an evaluation of the current NCD financing system. Finally, this study demonstrates how evidence gap mapping could not only identify data gaps in the diabetes care continuum but also support country‐specific actionable solutions, an approach that can also be applied to other LMICs to strengthen chronic disease management.

## Funding

This research received internal grant from Graduate School of Medical Science, University Medical Center Groningen.

## Conflicts of Interest

M.J.P. reports grants and honoraria from various pharmaceutical companies, including those developing, producing and marketing diabetes drugs. However, these grants and honoraria are not related to this specific study. The remaining authors declare no conflicts of interest.

## Supporting information


**Data S1:** tmi70100‐sup‐0001‐Supinfo.docx.

## Data Availability

All data used in this review are available in the Supporting Information.

## References

[1] International Diabetes Federation , Diabetes Atlas, 11th ed. (International Diabetes Federation, 2025), https://diabetesatlas.org/resources/idf‐diabetes‐atlas‐2025/.

[2] IHME , Burden of Disease 2021 (University of Washington, 2021), https://vizhub.healthdata.org/gbd‐compare/.

[3] Ministry of Health of Republic of Indonesia , Indonesia National Health Research 2018 Report (Ministry of Health of Republic of Indonesia, 2019), https://repository.badankebijakan.kemkes.go.id/id/eprint/3514/1/Laporan%20Riskesdas%202018%20Nasional.pdf.

[4] Badan Kebijakan Pembangunan Kesehatan Ministy of Health of Republic of Indonesia , Survei Kesehatan Indonesia Dalam Angka (Indonesian Health Survey in Number) 2023 (Badan Kebijakan Pembangunan Kesehatan Ministry of Health of Republic of Indonesia, 2023), https://layanandata.kemkes.go.id/katalog‐data/ski/ketersediaan‐data/ski‐2023.

[5] Directorate of Non‐communicable Disease, Ministry of Health of Indonesia , Pandu PTM (Ministry of Health of Republic of Indonesia, 2017), https://www.scribd.com/document/385364672/PANDU‐PTM‐ENGLISH.

[6] World Health Organization , WHO Package of Essential Noncommunicable (PEN) Disease Intervention for Primary Health Care [Internet] (World Health Organization, 2020), https://www.who.int/publications/i/item/who‐package‐of‐essential‐noncommunicable‐(pen)‐disease‐interventions‐for‐primary‐health‐care.

[7] M. Wahidin , A. Achadi , B. Besral , et al., “Projection of Diabetes Morbidity and Mortality Till 2045 in Indonesia Based on Risk Factors and NCD Prevention and Control Programs,” Scientific Reports 14, no. 1 (2024): 5424, 10.1038/s41598-024-54563-2. 38443384 PMC10914682

[8] R. Devi , K. Kanitkar , R. Narendhar , K. Sehmi , and K. Subramaniam , “A Narrative Review of the Patient Journey Through the Lens of Non‐Communicable Diseases in Low‐ and Middle‐Income Countries,” Advanced Therapies 37, no. 12 (2020): 4808–4830, 10.1007/s12325-020-01519-3. PMC7553852 33052560

[9] T. Bharatan , R. Devi , P. H. Huang , et al., “A Methodology for Mapping the Patient Journey for Noncommunicable Diseases in Low‐ and Middle‐Income Countries,” Journal of Healthcare Leadership 13 (2021): 35–46, 10.2147/JHL.S288966. 33542673 PMC7853412

[10] E. L. Davies , L. N. Bulto , A. Walsh , et al., “Reporting and Conducting Patient Journey Mapping Research in Healthcare: A Scoping Review,” Journal of Advanced Nursing 79, no. 1 (2023): 83–100, 10.1111/jan.15479. 36330555 PMC10099758

[11] A. Akhlaq , B. McKinstry , K. B. Muhammad , and A. Sheikh , “Barriers and Facilitators to Health Information Exchange in Low‐ and Middle‐Income Country Settings: A Systematic Review,” Health Policy and Planning 31, no. 9 (2016): 1310–1325, 10.1093/heapol/czw056. 27185528

[12] M. Aminpour , A. Aryankhesal , A. A. Hashjin , and H. Pourasghari , “Implementation of the Package of Essential Non‐Communicable (PEN) Disease Interventions in Low‐Resource Settings: A Systematic Review,” Iranian Journal of Public Health 53, no. 10 (2024): 2226–2238, 10.18502/ijph.v53i10.16700. 39544853 PMC11557760

[13] J. P. Tripathy and S. Mishra , “How Effective Was Implementation of the Package of Essential Non‐Communicable Disease (PEN) Interventions: A Review of Evidence?,” Diabetes and Metabolic Syndrome: Clinical Research and Reviews 15, no. 5 (2021): 102266, 10.1016/j.dsx.2021.102266. 34496339

[14] B. Snilstveit , M. Vojtkova , A. Bhavsar , J. Stevenson , and M. Gaarder , “Evidence & Gap Maps: A Tool for Promoting Evidence Informed Policy and Strategic Research Agendas,” Journal of Clinical Epidemiology 79 (2016): 120–129, 10.1016/j.jclinepi.2016.05.015. 27387966

[15] F. Campbell , A. C. Tricco , Z. Munn , et al., “Mapping Reviews, Scoping Reviews, and Evidence and Gap Maps (EGMs): The Same but Different—The “Big Picture” Review Family,” Systematic Reviews 12, no. 1 (2023): 45, 10.1186/s13643-023-02178-5. 36918977 PMC10014395

[16] S. A. Soelistijo , H. Novida , A. Rudijanto , and P. Soewando , Guideline of Management and Prevention of Type 2 Diabetes Mellitus in Indonesia (PERKENI, 2021), https://pbperkeni.or.id/wp‐content/uploads/2021/11/22‐10‐21‐Website‐Pedoman‐Pengelolaan‐dan‐Pencegahan‐DMT2‐Ebook.pdf.

[17] R. Agustina , T. Dartanto , R. Sitompul , et al., “Indonesian Health Systems Group. Universal health coverage in Indonesia: concept, progress, and challenges,” Lancet 393, no. 10166 (2019): 7 –102, 10.1016/s0140-6736(18)31647-7. 30579611

[18] Z. Munn , S. Moola , K. Lisy , D. Riitano , and C. Tufanaru , “Methodological Guidance for Systematic Reviews of Observational Epidemiological Studies Reporting Prevalence and Cumulative Incidence Data,” International Journal of Evidence‐Based Healthcare 13, no. 3 (2015): 147–153, 10.1097/xeb.0000000000000054. 26317388

[19] T. H. Barker , C. B. Migliavaca , C. Stein , et al., “Conducting Proportional Meta‐Analysis in Different Types of Systematic Reviews: A Guide for Synthesisers of Evidence,” BMC Medical Research Methodology 21, no. 1 (2021): 189, 10.1186/s12874-021-01381-z. 34544368 PMC8451728

[20] J. J. Barendregt , S. A. Doi , Y. Y. Lee , R. E. Norman , and T. Vos , “Meta‐Analysis of Prevalence,” Journal of Epidemiology and Community Health 67, no. 11 (2013): 974–978, 10.1136/jech-2013-203104. 23963506

[21] J. IntHout , J. P. Ioannidis , and G. F. Borm , “The Hartung‐Knapp‐Sidik‐Jonkman Method for Random Effects Meta‐Analysis Is Straightforward and Considerably Outperforms the Standard DerSimonian‐Laird Method,” BMC Medical Research Methodology 14, no. 1 (2014): 25, 10.1186/1471-2288-14-25. 24548571 PMC4015721

[22] C. B. Migliavaca , C. Stein , V. Colpani , et al., “Meta‐Analysis of Prevalence: *I* ^2^ Statistic and How to Deal With Heterogeneity,” Research Synthesis Methods 13, no. 3 (2022): 363–367, 10.1002/jrsm.1547. 35088937

[23] R. C. M. Van Aert , C. H. Schmid , D. Svensson , and D. Jackson , “Study Specific Prediction Intervals for Random‐Effects Meta‐Analysis: A Tutorial: Prediction Intervals in Meta‐Analysis,” Research Synthesis Methods 12, no. 4 (2021): 429–447, 10.1002/jrsm.1490. 33939307 PMC8361666

[24] H. Wickham , ggplot2: Elegant Graphics for Data Analysis (Springer international publishing, 2016).

[25] C. Graul , leafletR: Interactive Web‐Maps Based on the Leaflet JavaScript Library, 2016, http://cran.r‐project.org/package=leafletR.

[26] G. Schwarzer , meta: General Package for Meta‐Analysis, 2006, https://CRAN.R‐project.org/package=meta.

[27] A. Amir , S. Khader , Z. El Chami , S. Bahlas , M. Bakir , and S. Arifeen , “Management of Neuropathic Pain in Patients With Diabetic Peripheral Neuropathy and Low Back Pain in Saudi Arabia: Evidence and Gaps,” Journal of Family and Community Medicine 28, no. 3 (2021): 155, 10.4103/jfcm.jfcm_79_21. 34703375 PMC8496701

[28] R. B. Espiritu‐Picar , B. J. Matawaran , J. M. Lim , and P. Ratnasingham , “Mapping the Journey of Patients With Painful Diabetic Peripheral Neuropathy in The Philippines,” Acta Medica Philippina 57, no. 6 (2023): 46–51, 10.47895/amp.vi0.4471. PMC11522588 39483691

[29] P. C. Lim , R. Rajah , C. Y. Lee , T. Y. Wong , S. S. A. Tan , and S. A. Karim , “Systematic Review and Meta‐Analysis of Diabetes Knowledge Among Type 2 Diabetes Patients in Southeast Asia,” Review of Diabetic Studies 17, no. 2 (2021): 82–89, 10.1900/RDS.2021.17.82. PMC9380083 34852899

[30] J. C. Miranda , S. A. Raza , B. Kolawole , et al., “Enhancing Diabetes Care in LMICs: Insights From a Multinational Consensus,” Pakistan Journal of Medical Sciences 39, no. 6 (2023): 1889–1906, 10.12669/pjms.39.7.8881. PMC10626083 37936776

[31] W. Adisasmito , V. Amir , A. Atin , A. Megraini , and D. Kusuma , “Geographic and Socioeconomic Disparity in Cardiovascular Risk Factors in Indonesia: Analysis of the Basic Health Research 2018,” BMC Public Health 20, no. 1 (2020): 1004, 10.1186/s12889-020-09099-1. 32586296 PMC7318418

[32] F. Kurniawan , F. S. Sigit , S. Trompet , et al., “Lifestyle and Clinical Risk Factors in Relation With the Prevalence of Diabetes in the Indonesian Urban and Rural Populations: The 2018 Indonesian Basic Health Survey,” Preventive Medicine Reports 38 (2024): 102629, 10.1016/j.pmedr.2024.102629. 38375173 PMC10874845

[33] R. B. Fanda , A. Probandari , Y. Yuniar , et al., “The Availability of Essential Medicines in Primary Health Centres in Indonesia: Achievements and Challenges Across the Archipelago,” Lancet Regional Health ‐ Southeast Asia 22 (2024): 100345, 10.1016/j.lansea.2023.100345. 38482146 PMC10934320

[34] A. L. Joseph , H. Monkman , A. Kushniruk , and Y. Quintana , “Exploring Patient Journey Mapping and the Learning Health System: Scoping Review,” JMIR Human Factors 10 (2023): e43966, 10.2196/43966. 36848189 PMC10012009

[35] N. L. Masruroh , A. F. Pangastuti , N. Melizza , and A. D. Kurnia , “Level of Knowledge and Family Support Toward Medication Adherence Among Patient With Diabetes Mellitus in Malang, Indonesia,” Indian Journal of Forensic Medicine and Toxicology 15, no. 1 (2021): 1406–1413, 10.37506/ijfmt.v15i1.13610.

[36] W. R. B. Santosa , N. Nambiar , and E. Abdullah , “Uncovering the Multifaceted Influences on Type‐2 Diabetes Mellitus Incidence in Public Health Centre, Indonesia,” Malaysian Journal of Nursing 16, no. 1 (2024): 80–88, 10.31674/mjn.2024.v16i01.009.

[37] M. Fritz , M. Grimm , H. T. My Hanh , et al., “Effectiveness of Community‐Based Diabetes and Hypertension Prevention and Management Programmes in Indonesia and Viet Nam: A Quasi‐Experimental Study,” BMJ Global Health 9, no. 5 (2024): e015053, 10.1136/bmjgh-2024-015053. PMC11116884 38777393

[38] J. Manne‐Goehler , P. Geldsetzer , K. Agoudavi , et al., “Health System Performance for People With Diabetes in 28 Low‐ and Middle‐Income Countries: A Cross‐Sectional Study of Nationally Representative Surveys,” PLoS Medicine 16, no. 3 (2019): e1002751, 10.1371/journal.pmed.1002751. 30822339 PMC6396901

[39] J. Mulyanto , Y. Wibowo , D. A. Ernawati , D. W. D. Lestari , and D. S. Kringos , “Exploring Inequalities in the Use, Quality, and Outcome of the Diabetes Management Program of Indonesian National Health Insurance,” Health Equity 7, no. 1 (2023): 644–652, 10.1089/heq.2023.0025. 37786529 PMC10541918

[40] M. Fritz , M. Grimm , I. Weber , E. Yom‐Tov , and B. Praditya , “Can Social Media Encourage Diabetes Self‐Screenings? A Randomized Controlled Trial With Indonesian Facebook Users,” npj Digital Medicine 7, no. 1 (2024): 245, 10.1038/s41746-024-01246-x. 39271847 PMC11399376

[41] M. Griselda , S. D. Alfian , I. A. Wicaksono , M. Wawruch , and R. Abdulah , “Findings From the Indonesian Family Life Survey on Patterns and Factors Associated With Multimorbidity,” Scientific Reports 13, no. 1 (2023): 18607, 10.1038/s41598-023-42603-2. 37903815 PMC10616186

[42] J. Mulyanto , D. S. Kringos , and A. E. Kunst , “Socioeconomic Inequalities in the Utilisation of Hypertension and Type 2 Diabetes Management Services in Indonesia,” Tropical Medicine and International Health 24 (2019): 1301–1310, 10.1111/tmi.13303. 31465584 PMC6899976

[43] B. Hidayat , R. V. Ramadani , A. Rudijanto , P. Soewondo , K. Suastika , and J. Y. Siu Ng , “Direct Medical Cost of Type 2 Diabetes Mellitus and Its Associated Complications in Indonesia,” Value Health Regional Issues 28 (2022): 82–89, 10.1016/j.vhri.2021.04.006. 34839111

[44] M. J. Stirratt , J. Dunbar‐Jacob , H. M. Crane , et al., “Self‐Report Measures of Medication Adherence Behavior: Recommendations on Optimal Use,” Translational Behavioral Medicine 5, no. 4 (2015): 470–482, 10.1007/s13142-015-0315-2. 26622919 PMC4656225

[45] M. Azharuddin , M. Adil , M. Sharma , and B. Gyawali , “A Systematic Review and Meta‐Analysis of Non‐Adherence to Anti‐Diabetic Medication: Evidence From Low‐ and Middle‐Income Countries,” International Journal of Clinical Practice 75, no. 11 (2021): e14717, 10.1111/ijcp.14717. 34378293

[46] S. Pengpid and K. Peltzer , “Utilization of Traditional and Complementary Medicine in Indonesia: Results of a National Survey in 2014–15,” Complementary Therapies in Clinical Practice 33 (2018): 156–163, 10.1016/j.ctcp.2018.10.006. 30396615

[47] S. D. Alfian , H. Sukandar , N. Arisanti , and R. Abdulah , “Complementary and Alternative Medicine Use Decreases Adherence to Prescribed Medication in Diabetes Patients,” Annals of Tropical Medicine and Public Health 9 (2016): 174–179, 10.4103/1755-6783.179108.

[48] N. N. M. Soetedjo , S. M. McAllister , C. Ugarte‐Gil , et al., “Disease Characteristics and Treatment of Patients With Diabetes Mellitus Attending Government Health Services in Indonesia, Peru, Romania and South Africa,” Tropical Medicine & International Health 23, no. 10 (2018): 1118–1128, 10.1111/tmi.13137. 30106222

[49] H. Permana , R. C. Koesoemadinata , N. N. M. Soetedjo , et al., “Diabetes Mellitus Patients in Indonesia: Management in a Tertiary Hospital Compared to Primary Health Care,” Universal Medicap 41, no. 2 (2022): 157–168, 10.18051/UnivMed.2022.v41.157-168.

[50] F. R. Muharram , J. B. Swannjo , D. L. Tahapary , S. A. Nasution , and D. Oceandy , “Diabetes Care Performance in Indonesia: A Serial Cross‐Sectional Analysis of Behavioral, Clinical, and Laboratory Outcomes From 2013 to 2023,” Lancet Regional Health 65 (2025): 101759, 10.1016/j.lanwpc.2025.101759. PMC12681878 41362420

[51] V. Widyaningsih , R. P. Febrinasari , E. P. Pamungkasari , et al., “Missed Opportunities in Hypertension Risk Factors Screening in Indonesia: A Mixed‐Methods Evaluation of Integrated Health Post (POSBINDU) Implementation,” BMJ Open 12, no. 2 (2022): e051315, 10.1136/bmjopen-2021-051315. PMC8862503 35190419

[52] S. Nappoe , H. Djasri , and M. Kurniawan , Chronic Disease Management Programme (PROLANIS) in Indonesia: Case Study (World Health Organization, Organisation for Economic Cooperation and Development, 2023), https://extranet.who.int/kobe_centre/sites/default/files/pdf/Indonesia_case_study_policy_brief.pdf.

[53] Ministry of Health of the Republic of, Indonesia , Blueprint for Digital Health Transformation Strategy 2024 (Ministry of Health of the Republic of Indonesia, 2021), https://oss2.dto.kemkes.go.id/artikel‐web‐dto/ENG‐Blueprint‐for‐Digital‐Health‐Transformation‐Strategy‐Indonesia%202024.pdf.

[54] J. F. Levesque , M. F. Harris , and G. Russell , “Patient‐Centred Access to Health Care: Conceptualising Access at the Interface of Health Systems and Populations,” International Journal for Equity in Health 12, no. 1 (2013): 18, 10.1186/1475-9276-12-18. 23496984 PMC3610159

[55] I. Cahyaningsih , M. R. Rokhman , N. Maziyyah , E. Niamuzisilawati , K. Taxis , and P. Denig , “Translation and Validation of the Diabetes Knowledge Questionnaire in Indonesian Patients With Type 2 Diabetes,” Science of Diabetes Self‐Management Care 50, no. 6 (2024): 484–496, 10.1177/26350106241287445. 39425573 PMC11600658

[56] World Health Organization , Patients for Patient Safety, 2024, https://www.who.int/initiatives/patients‐for‐patient‐safety.

[57] Y. Bombard , G. R. Baker , E. Orlando , et al., “Engaging Patients to Improve Quality of Care: A Systematic Review,” Implementation Science 13, no. 1 (2018): 98, 10.1186/s13012-018-0784-z. 30045735 PMC6060529

[58] A. Conklin , Z. Morris , and E. Nolte , “What Is the Evidence Base for Public Involvement in Health‐Care Policy?: Results of a Systematic Scoping Review,” Health Expectations 18, no. 2 (2015): 153–165, 10.1111/hex.12038. 23252574 PMC5060770

[59] T. Gadsden , S. Sujarwoto , N. Purwaningtyas , et al., “Understanding Community Health Worker Employment Preferences in Malang District, Indonesia, Using a Discrete Choice Experiment,” BMJ Global Health 7, no. 8 (2022): e008936, 10.1136/bmjgh-2022-008936. PMC9379506 35953209

[60] Y. Primanda and D. I. Fatah , “Knowledge and Experience of Community Health Volunteer (Cadre) on Type 2 Diabetes Mellitus Management in Yogyakarta,” Open Access Macedonian Journal of Medical Sciences 9, no. 4 (2021): 240–244, 10.3889/oamjms.2021.5863.

[61] D. T. Stein , N. Sudharsanan , S. Dewi , J. Manne‐Goehler , F. Witoelar , and P. Geldsetzer , “Change in Clinical Knowledge of Diabetes Among Primary Healthcare Providers in Indonesia: Repeated Cross‐Sectional Survey of 5105 Primary Healthcare Facilities,” BMJ Open Diabetes Research & Care 8, no. 1 (2020): e001415, 10.1136/bmjdrc-2020-001415. PMC7536835 33020133

[62] Ministry of Health of Republic of Indonesia , National Health Accounts Indonesia 2021, 2023, https://kms.kemkes.go.id/contents/1715911450956‐NationalHealthAccountsTahun2021comp.pdf.

[63] A. Fuady , M. Anindhita , M. Haniifah , et al., “Bridging the Gap: Financing Health Promotion and Disease Prevention in Indonesia,” Health Research Policy and Systems 22, no. 1 (2024): 146, 10.1186/s12961-024-01206-7. 39407235 PMC11481782

