# Supplementary material for: Where Are the Gaps in Diabetes Care? An Evidence Gap Mapping of the Diabetes Patient Journey in Indonesia
Source: Trop Med Int Health. 2026 Feb 4;31(4):409–21. doi: 10.1111/tmi.70100 (PMC13050617; doi:10.1111/tmi.70100)
Supplement: Supplementary file 1 — Data S1: tmi70100‐sup‐0001‐Supinfo.docx. [file TMI-31-409-s001.docx]

**Where are the Gaps in Diabetes Care? An Evidence Gap Map of the Diabetes Patient Journey in Indonesia**

Supplementary materials

**Table of Contents**

[**1.** **Search Strategy** 2](#_Toc200719561)

[PubMed 2](#_Toc200719562)

[Web of Sciences 2](#_Toc200719563)

[Scopus 2](#_Toc200719564)

[Embase 2](#_Toc200719565)

[**2.** **Excluded Studies Due to Duplicated Databases** 4](#_Toc200719566)

[**3.** **Details of the Included Studies** 5](#_Toc200719567)

[**4.** **Details of Risk Bias Assessment** 34](#_Toc200719568)

[**5.** **Pooled Prevalence Estimates** 42](#_Toc200719569)

[**References** 52](#_Toc200719570)

# **Search Strategy**

## PubMed

((diabetes) OR (type 2 diabetes) OR (high blood sugar) OR (hyperglycemia)) AND ((epidemiology) OR (prevalence) OR (incidence) OR (national) OR (survey) OR (registry)) AND

((awareness) OR (knowledge) OR (health literacy) OR (health education) OR (screening) OR (diagnosed) OR (undiagnosed) OR (treatment) OR (treated) OR (untreated) OR (control) OR (controlled) OR (uncontrolled) OR (adherence) OR (compliance) OR (adhere) OR (therapy) OR (non-adherence)) AND

((Indonesia) or (java) or (sumatera) or (kalimantan) or (sulawesi) or (papua))

## Web of Sciences

((diabetes) OR (type 2 diabetes) OR (high blood sugar) OR (hyperglycemia))  AND ((epidemiology) OR (prevalence) OR (incidence) OR (national) OR (survey) OR (registry))  AND ((awareness) OR (knowledge) OR (health literacy) OR (health education) OR (screening) OR (diagnosed) OR (undiagnosed) OR (treatment) OR (treated) OR (untreated) OR (control) OR (controlled) OR (uncontrolled) OR (adherence) OR (compliance) OR (adhere) OR (therapy) OR (non-adherence))  AND ((Indonesia) OR (Java) OR (Sumatera) OR (Kalimantan) OR (Sulawesi) OR (Papua))

## Scopus

TITLE-ABS-KEY((diabetes) OR (type 2 diabetes) OR (high blood sugar) OR (hyperglycemia))

AND TITLE-ABS-KEY((epidemiology) OR (prevalence) OR (incidence) OR (national) OR (survey) OR (registry))

AND TITLE-ABS-KEY((awareness) OR (knowledge) OR (health literacy) OR (health education) OR (screening) OR (diagnosed) OR (undiagnosed) OR (treatment) OR (treated) OR (untreated) OR (control) OR (controlled) OR (uncontrolled) OR (adherence) OR (compliance) OR (adhere) OR (therapy) OR (non-adherence))

AND TITLE-ABS-KEY((Indonesia) OR (Java) OR (Sumatera) OR (Kalimantan) OR (Sulawesi) OR (Papua))

## Embase

(('diabetes'/exp OR diabetes OR 'type 2 diabetes'/exp OR 'type 2 diabetes' OR (type AND ('2'/exp OR 2) AND ('diabetes'/exp OR diabetes)) OR 'high blood sugar' OR (high AND ('blood'/exp OR blood) AND ('sugar'/exp OR sugar)) OR 'hyperglycemia'/exp OR hyperglycemia) AND ('epidemiology'/exp OR epidemiology OR 'prevalence'/exp OR prevalence OR 'incidence'/exp OR incidence OR national OR 'survey'/exp OR survey OR 'registry'/exp OR registry) AND ('awareness'/exp OR awareness OR 'knowledge'/exp OR knowledge OR 'health literacy'/exp OR 'health literacy' OR (('health'/exp OR health) AND ('literacy'/exp OR literacy)) OR 'health education'/exp OR 'health education' OR (('health'/exp OR health) AND ('education'/exp OR education)) OR 'screening'/exp OR screening OR diagnosed OR undiagnosed OR 'treatment'/exp OR treatment OR treated OR untreated OR 'control'/exp OR control OR controlled OR uncontrolled OR 'adherence'/exp OR adherence OR 'compliance'/exp OR compliance OR adhere OR 'therapy'/exp OR therapy OR 'non adherence') AND ('indonesia'/exp OR indonesia OR java OR sumatera OR kalimantan OR sulawesi OR 'papua'/exp OR papua) AND [2014-2025]/py) AND ('article'/it))

# **Excluded Studies Due to Duplicated Databases**

| **No** | **Tittle** | **Citation** |
| --- | --- | --- |
| 1 | Prevalence and risk factors for chronic kidney disease in Indonesia: An analysis of the National Basic Health Survey 2018 | Hustrini, N.M., et. al (2022) (1) |
| 2 | The prevalence of edentulism and their related factors in Indonesia, 2014/15 | Pengpid, S., et. al (2018) (2) |
| 3 | Joint effect of high blood pressure and physical inactive on diabetes mellitus: a population-based cross-sectional survey | Hanafi, A.S., et. al (2020) (3) |
| 4 | Sociodemographic and behavioural risk factors associated with low awareness of diabetes mellitus medication in Indonesia: Findings from the Indonesian Family Life Survey (IFLS-5) | Khoiry Q.A., et. al (2023) (4) |
| 5 | Risk Factors for Diabetes Mellitus In Indonesia: Analysis Of IFLS Data 2014 | Afifah, A.M.N., et. al (2022) (5) |
| 6 | Prevalence and determinants of obesity among individuals with diabetes in Indonesia | Azam, M., et. al (2023) (6) |
| 7 | Modifiable risk factors in adults with and without prior cardiovascular disease: findings from the Indonesian National Basic Health Research | Arsyad D.S., et. al (2022) (7) |
| 8 | Hypertension, diabetes and cognitive impairment among elderly | Erlianti, C.P., et. al (2022) (8) |
| 9 | Predictor risk of diabetes mellitus in Indonesia, based on national health survey | Nugroho, P.S., et. al (2020) (9) |
| 10 | Association between Hypertension with the Incidence of Type 2 Diabetes Mellitus in South Kalimantan (Data Analysis of Indonesia Family Life Survey 5 Year 2014) | Oktaviannoor, H., et. al (2020) (10) |
| 11 | Determinants of Cardiovascular Diseases in the Elderly Population in Indonesia: Evidence from Population-Based Indonesian Family Life Survey (IFLS) | Zakaria, S.I., et. al (2022) (11) |
| 12 | Geographic and socioeconomic disparity in cardiovascular risk factors in Indonesia: analysis of the Basic Health Research 2018 | Adisasmito, W., et. al (2020) (12) |
| 13 | Indonesian tooth loss predictor in middle-aged and elderly populations based on sociodemographic factors and systemic disease: a cross-sectional study | Susilawati, S., et.al (2025) (13) |
| 14 | The same risk factors of hypertension in women in rural and urban areas (following analysis of data Indonesia Family Life survey 5 in 2014) | Dwi L.F., et.al (2020) (14) |
| 15 | Psychological distress among middle-aged adults with Diabetes Mellitus: findings from the Indonesia national population health survey (*Riset Kesehatan Dasar*, Riskesdas 2018) | Dilaga, M.S., et.al (2025) (15) |
| 16 | The impact of lifestyle changes on the prevalence of prediabetes and diabetes in urban and rural Indonesia: results from the 2013 and 2018 Indonesian basic health research (Riskesdas) survey | Liberty, I.A., et.al (2024) (16) |
| 17 | The association between depressive symptoms, access to diabetes care, and glycemic control in five middle-income countries | Merkel, L., et.al (2024) (17) |
| 18 | Medication adherence contributes to an improved quality of life in Type 2 Diabetes Mellitus patients: a cross-sectional study | Alfian S.D., et.al (2016) (18) |

# **Details of the Included Studies**

| **No** | **Authors** | **Study Design** | **Settings** | **Touchpoints** | **Measurement Method** | **Sample Size** | **Prevalence (%)** | **Quality** |
| --- | --- | --- | --- | --- | --- | --- | --- | --- |
| 1 | Aditama, L., et.al (2020) (19) | Cross-sectional | - T2D patients who used oral antidiabetic medication were recruited consecutively from 17 PHCs in East Java. - 80% were female, and 67.5% were >59 years old. | Adherence | Adopted Pharmaceutical Care Practice questionnaires. A mean score equal to 1 was considered adherent | 40 | 20.00 | Low |
| 2 | Alfian S.D., et.al (2016) (20) | Cross-sectional | - T2D patients who were prescribed glucose-lowering medication for at least 3 months were consecutively recruited from a secondary-level care facility in West Java from February to April 2014. - 71% were female, and 61% were > 59 years old. | Adherence | Morisky Medication Adherence Scale with a cut-off value of > 5 to define medium to high adherence. | 114 | 55.30 | Low |
| 3 | Akrom, A., et.al (2019) (21) | Cross-sectional | - T2D patients without complications and who use at least one oral antidiabetic drug for at least 3 months were consecutively recruited from a PHC in the Special Region of Yogyakarta in 2018. - 68.9% were female, and 75% were < 60 years old. | Adherence | Medication Adherence Rating Scale with unclear cut-off value. | 122 | 81.00 | Low |
| 4 | Amelia and Harahap (2019) (22) | Cross-sectional | - Hospitalized patients in one of the hospitals in North Sumatra were consecutively recruited - 53.3% were female and 65.5% were > 45 years old | Diagnosis | Medical record | 180 | 21.67 | Low |
| 5 | Amelia, R., et.al (2024) (23) | Cross-sectional | - T2D patients who visited and underwent medical follow-up in PHC in North Sumatra were consecutively recruited from June to August 2022. - 70.6% were female, and 63.6% were > 55 years old. | Control | HbA1c < 7% was examined via venous blood puncture. | 89 | 15.73 | Low |
| 6 | Amiruddin, R., et.al (2021) (24) | Quasi-experimental | - T2D patients living in the area of 2 PHCs in South Sulawesi were consecutively recruited in 2020. - 71% were female and >60% were > 45 years old. | Treatment | Medical record. | 60 | 98.33 | Low |
| 7 | Ananda, R.D.S, et.al (2019) (25) | Cross-sectional | - T2D patients registered at the Sleman Health and Demographic Surveillance System in May 2019 who used the same anti-diabetic treatment for at least 6 months were consecutively recruited. - 59.3% were female, and 68.5% were < 65 years old. | Adherence | Morisky Medication Adherence Scale 8, with a cut-off value of > 5 to define moderate to good adherence. | 108 | 76.85 | Low |
| 8 | Ardiany, D., et.al (2022) (26) | Cross-sectional | - T2D patients at the tertiary-level hospital in Surabaya were consecutively recruited between July and December 2019. - 59.7% were female, and mean age was 54 years old. | Control | HbA1c < 7% was examined during the study. | 72 | 23.61 | Low |
| 9 | Arifin, B., et.al (2019) (27) | Cross-sectional | - T2D patients were consecutively recruited from three PHCs or general practitioner clinics in East Java, Central Java, and West Java from 2015 to 2016. - 43% were female, and the mean age was 60 ± 10 years. | Treatment | Self-reported diabetes medication from the patient’s doctor. | 632 | 95.00 | Medium |
| 10 | Arifin, B., et.al (2019) (28) | Cross-sectional | - T2D patients were consecutively recruited from PHCs and secondary care level in Java and Sulawesi from 2015 to 2017. - 57% were female, and the mean age was 59.32 ± 9.7 years. | Treatment | Self-reported diabetes medication from the patient’s doctor. | 907 | 94.59 | Medium |
| 11 | Arliny, Y., et.al (2022) (29) | Cross-sectional | - T2D patients without complications and treated in two secondary or tertiary level care in Jakarta were consecutively recruited from December 201y to January 2019. - 60.7% were female, and 83% were > 49 years old. | Control | Self-reported HbA1c < 7%. | 242 | 38.84 | Low |
| 12 | Asril, N.M., et.al (2020) (30) | Cross-sectional | - T2D patients from a PHC in Bali were consecutively recruited from August to October 2017. - 56.7% were female, and the median age was 54.6. | Treatment | Medical record. | 203 | 61.08 | Low |
| 13 | Azam, M., et.al (2023) (31) | Cross-sectional | - Secondary data analysis of the National Health Survey 2018 among the sample who had self-reported a diabetes diagnosis by a doctor. - 67% were female, and 82.9% were > 44 years old. | Treatment | Self-reported use of any diabetes medication. | 3911 | 78.77 | Medium |
|  |  |  |  | Adherence | Self-reported adherence to antidiabetic medication (yes/no). | 3911 | 58.45 | Medium |
| 14 | Azmiardi, A., et.al (2023) | Cross-sectional | - Secondary data analysis of the Indonesian Family Life Survey 2014/2015 among the sample who had self-reported a diabetes diagnosis by a doctor. - 53.75% were female, and 64.99% were > 49 years old. | Adherence | Self-reported adherence to antidiabetic medication (yes/no). | 774 | 52.84 | Medium |
| 15 | Bhaskara, G., et.al (2022) (32) | Cross-sectional | - T2D patients who visited a secondary level care in Bali were consecutively recruited from January to April 2021. - 60.5% were female, and 64% were of productive age. | Control | HbA1c < 7% was examined during the study. | 124 | 41.93 | Low |
| 16 | Darmada, and Wulandari (2020) (33) | Cross-sectional | - T2D patients who were on oral diabetic medication for at least 6 months were consecutively recruited from a secondary hospital in Bali. - 58.8% were men, and the mean age was 58.6 years. | Adherence | Medication Adherence Rating Scale with a cut-off value of >24 to define moderate to high adherence. | 68 | 17.64 | Low |
| 17 | Dewanti, L., et.al (2024) (34) | Cross-sectional | - T2D patients who regularly visited a PHC in East Java were consecutively recruited from July to November 2018. - 64% were female, and 54% were > 50 years old. | Control | HbA1c < 7% was taken from the laboratory data. | 81 | 24.69 | Low |
| 18 | Dwiyatna, S., et.al (2024) (35) | Cross-sectional | - T2D patients who use insulin were consecutively recruited from the hospital in East Java from May to July 2023. - 66% were female, and >50% were 50 years old. | Awareness | Diabetes Knowledge Questionnaire 24, with a cut-off value of > 9 to determine moderate to good diabetes awareness. | 141 | 87.94 | Low |
|  |  |  |  | Adherence | Adherence to Refills and Medications Scale with a cut-off value of < 13 to define adherence. | 141 | 33.33 | Low |
|  |  |  |  | Control | HbA1c < 7% | 141 | 36.17 | Low |
| 19 | Elnaem, M.H., et.al (2025) (36) | Cross-sectional | - T2D patients were consecutively recruited from online platforms. - Around 70% were female, and around 88% were < 50 years old. | Treatment | Self-reported use of any anti-diabetic medication. | 324 | 90.12 | Low |
|  |  |  |  | Adherence | Malaysian Medication Adherence Assessment Tool with a cut-off value of > 53 to determine adherence. | 324 | 5.5 | Low |
| 20 | Faridah, I.N., et.al (2022) (37) | Cross-sectional | - T2D patients who use traditional medicine for at least a month were consecutively recruited from 3 PHCs in the Special Region of Yogyakarta. - 57.3% were > 60 years old, and 70.9% were female. | Adherence | Medication Adherence Rating Scale with a cut-off value of > 24 to define adherence to antidiabetic medication. | 110 | 58.18 | Low |
| 21 | Fibriana, A.I., et.al (2020) (38) | Case-control | - Diabetic patients registered at a hospital in Central Java without TB (control group) were consecutively recruited between January and July 2019. - 55.6% were female, and 88.9% were 15 – 64 years old. | Adherence | Morisky Medication Adherence Scale with unclear cut-off value. | 45 | 68.88 | Low |
| 22 | Fritz, M., et.al (2024) (39) | Quasi-experimental | - The general population residing in selected community health posts in East and Central Java was randomly selected and matched. - 82% were female, and the mean age was around 47 years. | Screening | Self-reported blood sugar measurement. | 1,552 | 60.11 | Low |
|  |  |  |  | Diagnosed | Self-reported diabetes diagnosis by doctor or healthcare professional. | 1,552 | 7.22 | Low |
| 23 | Fritz, M., et.al (2024) (40) | RCT | - All Facebook users who were reached by the “Ada Gula, Ada Diabetes” campaign were randomly exposed to different types of ads between March 15 and April 5, 2022. - 44% were female, and 52% were > 44 years old. | Screening | Completion of the FINDRISC questionnaire. | 286,776 | 0.51 | Medium |
| 24 | Handayani, O.W.K., et.al (2019) (41) | Cross-sectional | - Outpatient T2D patients were randomly selected from 4 community health centers’ working areas in the Special Region of Yogyakarta. - 69.8% were > 55 years old. | Adherence | Unclear method. | 119 | 76.47 | Low |
| 25 | Hendrianingtyas, M., et. al (2020) (42) | Cross-sectional | - Outpatient T2D patients were consecutively recruited in one of the hospitals in Central Java between June and July 2019. - 52.2% were male, and the median age was 62 (37 – 75) | Control | HbA1c < 7% | 69 | 36.23 | Low |
| 26 | Herwana and Febiana (2025) (43) | Cross-sectional | - T2D patients were consecutively recruited among the general population in the Jakarta Special Region during a community engagement activity between June and November 2022. - 79.5% were female and 64.4% were aged 35 – 60 years old. | Treatment | Self-report of any anti-diabetic medication use. | 73 | 61.64 | Low |
|  |  |  |  | Control | HbA1c < 7% | 73 | 26.03 | Low |
| 27 | Hidayat, B., et.al (2022) (44) | Retrospective cohort | - Claim data of the Indonesian National Health Insurance between January 1 and December 31, 2016. - 57% were female, and 39% were > 60 years old. | Treatment | Prescription of 23 days of anti-diabetic medication supply. | 812,204 | 32.40 | High |
| 28 | Indrayanti, S., et.al (2019) (45) | Case-control | - Outpatient T2D patients in a hospital in West Java were consecutively recruited. - 53.3% were male, and the mean age was 56.09 ± 13.19 years. | Diagnosed | Medical record | 75 | 25.33 | Low |
| 29 | Jasmine, N.S., et.al (2020) (46) | Cross-sectional | - T2D patients using oral anti-diabetic drugs were consecutively recruited from a PHC in West Java. - 55.7% were female, and 70% were < 65 years. | Awareness | DKQ-24 with unclear cut-off value. | 113 | 56.64 | Low |
|  |  |  |  | Adherence | MMAS-8 with unclear cut-off value. | 113 | 31.86 | Low |
| 30 | Jaya, M.K.A., et.al (2024) (47) | Cross-sectional | - T2D patients using at least one antidiabetic medication were consecutively recruited from 2 hospitals in Bali. - 51% were female and 58% > 55 years old. | Control | HbA1c < 7% | 89 | 8.99 | Low |
| 31 | Jaya, M.K.A., et.al (2024) (48) | Case-control | - T2D ambulatory patients without hypoglycemia unawareness (control) were consecutively recruited from three hospitals in Bali between January 2023 and July 2024. - 45% were female, and 76% were aged 20 – 64 years. | Control | HbA1c < 7% from medical record. | 100 | 26.00 | Low |
| 32 | Julaiha, S., et.al (2019) (49) | Cross-sectional | - All T2D outpatient patients in a hospital in Lampung were recruited. - 70.5% were female, and 53% were < 60 years. | Adherence | MMAS-8 with a cut-off value of < 3. | 200 | 58.00 | Low |
| 33 | Kresnowati, et.al (2025) (50) | Cross-sectional | - All patients recorded in the national health insurance database in 2022, 2023, and 2024 were included. | Screening | History of diabetes risk screening by themselves or by health workers in primary health care. | 263,709,211 | 10.82 | High |
| 34 | Kristina, S.A., etl.al (2020) (51) | Cross-sectional | - T2D patients were randomly recruited from 8 hospitals in the western, central, and eastern regions of Indonesia. - 52.55% were female and 73.33% were > 40 years. | Treatment | Medical record. | 2,550 | 100.00 | High |
|  |  |  |  | Control | HbA1c < 7% | 2,550 | 30.59 | Medium |
| 35 | Kristina, S.A., etl.al (2021) (52) | Cross-sectional | - Adults aged 17 – 60 residing in the Special Region of Yogyakarta were randomly selected from the residential registry. - 55% were female and 93.3% were > 19 years old. | Awareness | 13 key questions about diabetes risk factors and symptoms, with a cut-off value of > 6 indicating good diabetes awareness. | 780 | 53.85 | Low |
|  |  |  |  | Diagnosis | Self-reported diabetes diagnosis. | 780 | 20.26 | Low |
| 36 | Kristanti, D., et.al (2021) (53) | Prospective cohort | - Secondary data analysis of the Bogor Cohort study using participants aged > 25 years. - 71.1% were female, and the mean age was 49.6 ± 9.9 years. | True Prevalence | Self-reported doctor diagnosis or blood glucose measurement indicating diabetes. | 3,077 | 9.13 | Low |
| 37 | Kurnia, A.D., et.al (2017) (54) | Cross-sectional | - T2D patients in a city in East Java were randomly selected from five PHCs using a multistage sampling method. - 70.1% were female, and 96.1% were > 40 years. | Awareness | Diabetes Knowledge Questionnaire with the cut-off value of > 13 for good diabetes awareness. | 127 | 68.50 | Low |
|  |  |  |  | Treatment | Self-report of any anti-diabetic medication use. | 127 | 84.25 | Low |
| 38 | Kurnia, A.D., et.al (2022) (55) | Cross-sectional | - T2D patients enrolled in PHC in a city in East Java were consecutively recruited. - 86.7% were female, and the mean age was 61.97 ± 7.85 years. | Treatment | Medical record. | 120 | 100.00 | Low |
| 39 | Kurniati, I., et.al (2024) (56) | Cross-sectional | - T2D patients were consecutively recruited from two hospitals in Bandar Lampung and West Java. - 45.2% were female, and the mean age was 59 years. | Control | HbA1c < 7% was examined via venous blood puncture. | 209 | 29.67 | Low |
| 40 | Kurniawan, F., et.al (2024) (57) | Cross-sectional | - Secondary data analysis of the National Health Survey 2018. - 50.3% were men, and the mean age was 42.6 years for urban area, and 50.5% were men, and the mean age was 44.5 years for rural area. | True Prevalence | Self-reported diabetes diagnosis or blood measurement indicating diabetes | Urban:  Rural: | Urban: 10.9 (10.4 - 11.5)  Rural: 11.00 (10.4 – 11.7) | High |
|  |  |  |  | Diagnosis | Self-reported diabetes diagnosis. | Urban:  Rural: | Urban: 3.8 (3.5 – 4.2)  Rural: 1.9 (1.6 – 2.1) | High |
| 41 | Lim, L.L., et.al (2023) (58) | Cross-sectional | - T2D patients treated with diet with or without glucose-lowering medication were consecutively recruited from several hospitals and community health centers in Indonesia between 2013 and 2015. - 52.6% were female and 75.1% were > 49 years old. | Control | HbA1c < 7% | 1,573 | 30.13 | Medium |
| 42 | Maharani, A., et.al (2019) (59) | Cross-sectional | - All eligible individuals from 8 villages in a city in East Java were recruited. - 56.6% were female, and the median age was 54.9 (10.7) years. | True Prevalence | Self-reported diabetes diagnosis, or was treated with any blood glucose-lowering medication, or had a random capillary blood glucose level > 199 mg/dL. | 22,093 | 9.8 | Medium |
| 43 | Makkulawu, A., et.al (2019) (60) | Cross-sectional | - 3 elderly people from 32 elderly houses in East Java were consecutively recruited. - 81.13% were female, and 65.48% were > 59 years. | Adherence | ARMS with a cut-off value of < 8. | 281 | 32.74 | Low |
| 44 | Malini, H., et.al (2022) (61) | Cross-sectional | - T2D patients from the outpatient clinics of a community health center in Indonesia were consecutively recruited. - 97.8% were > 40 years old, and 66.3% were female. | Adherence | Self-report daily medication use in the past 7 days. | 89 | 97 | Low |
| 45 | Masuroh, N.L, et.al (2021) (62) | Cross-sectional | - T2D patients were randomly selected in one community in a city in East Java. - 81.2% were female, and 71% were 18 – 65 years. | Awareness | Diabetes Knowledge Questionnaire with a cut-off value of > 55.99% to define good diabetes awareness. | 48 | 31.25 | Low |
|  |  |  |  | Adherence | Morisky Medication Adherence Scale with a cut-off value of > 6 to define adherence. | 48 | 68.75 | Low |
| 46 | Mayasari, D.S., et.al (2021) (63) | Cross-sectional | - T2D patients enrolled at the outpatient clinic of a hospital in the Special Region of Yogyakarta were consecutively recruited between April and July 2020. - 55.6% were male, and the median age was 55 (49 – 60) years. | Treatment | Medical record. | 81 | 97.53 | Low |
|  |  |  |  | Control | HbA1c < 7% was examined via peripheral blood measurement. | 81 | 28.40 | Low |
| 47 | Mulyanto, J., et.al (2019) (64) | Cross-sectional | - Secondary data analysis of the Indonesian Family Life Survey 2014/2015 among respondents who had blood glucose measurement. - 55.2% were women, and more than 40% were > 45 years. | True Prevalence | HbA1c ≥6.5% | 6,758 | 7.2 (6.6 – 7.8) | High |
|  |  |  |  | Screening | Self-reported blood glucose measurement during the past 12 months. | 6,758 | 11.2 (10.4 – 12.0) | High |
|  |  |  |  | Treatment | Self-report any anti-diabetes medication consumption | 485 | 13.0 (10.0 – 16.6) | High |
| 48 | Muhammadong, J., et.al (2024) (65) | Cross-sectional | - Residents of a village in South Sulawesi were randomly selected between March and May 2023. - 42% were male, and 72% were aged 17 – 25 years. | True Prevalence | FBG > 125 mg/dL or RCBG > 199 mg/dL combined with T2D classical symptoms. | 270 | 22.96 | Low |
| 49 | Nanda, O.D., et.al (2018) (66) | Cross-sectional | - T2D female patients were consecutively recruited in a PHC in East Java between January and June 2019. - 88% were > 50 years. | Adherence | MMAS-8 with a cut-off value of > 5. | 26 | 69.23 | Low |
| 50 | Natasya, A., et.al (2018) (67) | Cross-sectional | - T2D patients using pharmacological therapy for at least 3 months were consecutively recruited from an outpatient clinic in a general hospital in West Java. - 68.5% were female, and 58.3% were geriatric patients. | Adherence | Unclear method. | 108 | 44.4 | Low |
|  |  |  |  | Control | HbA1c <7% through measurement during the study. | 108 | 37.96 | Low |
| 51 | Nazriati, E., et.al (2018) (68) | Cross-sectional | - T2D patients registered at a PHC in Riau were randomly selected in 2017. - 62.5% were female, and 43% were > 55 years. | Awareness | DKQ-24 with unclear cut-off value. | 40 | 82.50 | Low |
|  |  |  |  | Adherence | MMAS-8 with unclear cut-off value. | 40 | 77.5 | Low |
| 52 | Ningsih, O.S., et.al (2024) (69) | Cross-sectional | - T2D patients were consecutively recruited from a hospital and a city health center in East Nusa Tenggara between February and April 2023. - 50% were female, and 62.5% were aged 45 – 59 years. | Treatment | Self-report of any anti-diabetic medication use. | 72 | 94.4 | Low |
| 53 | Notariza, K.R., et.al (2021) (70) | Cross-sectional | - T2D patients who were older than 60 years were consecutively recruited from a PHC in Central Sulawesi. - 61.7% were female, and the median age was 66 (66 – 82). | Treatment | Self-reported receiving any anti-diabetic medication. | 60 | 88.33 | Low |
| 54 | Nugrahaeni, D.K., et.al (2022) (71) | Cross-sectional | - T2D registered at the chronic disease management program at PHCs and a clinic in West Java were consecutively recruited. - 75% were female, and 57.5% were < 60 years. | Treatment | Self-report receiving any anti-diabetic medication. | 40 | 90.00 | Low |
| 55 | Nugroho, D.B., et.al (2022) (72) | Cross-sectional | - The general population of a city in a Special Region of Yogyakarta was randomly selected using a multistage random sampling method. - 64.5% were women, and the mean age was 48.64. | Diagnosed | Self-reported diabetes diagnosis by doctor or healthcare professional. | 4,611 | 10.04 | Medium |
| 56 | Pratama, I.P.Y, et.al (2019) (73) | Cross-sectional | - T2D patients were consecutively recruited from a hospital in the Special Region of Yogyakarta between September and October 2017. - 58% were female, and 61.5% were > 59 years old. | Awareness | Diabetes Knowledge Questionnaire 24 with an unclear cut-off value. | 200 | 100 | Low |
| 57 | Pratiwi, C., et.al (2022) (74) | Retrospective cohort | - All inpatient T2D patients admitted to a hospital in Jakarta Special Region from January 2016 to 2018 were included. - 55.2% were < 60 years old. - 51.2% were male. | Treatment | Medical record. | 475 | 77.89 | Medium |
| 58 | Pavitasari, A., et.al (2022) (75) | Case control | - The general population who visited the Vegan Festival Surabaya were consecutively recruited. - 58% were males, and the age ranged from 47 to 66 years. | Adherence | Morisky Medication Adherence Scale with an unclear cut-off value. | 22 | 90.91 | Low |
| 59 | Permana, H., et.al (2022) (76) | Cross-sectional | - T2D patients were consecutively recruited from a general hospital and 25 PHCs in West Java between December 2013 and February 2015. - 63.2% were female, and 83.5% > 49 years old. | Treatment | Self-reported receiving any anti-diabetic medication. | 809 | 91.97% | Low |
|  |  |  |  | Control | HbA1c < 7% | 230 | 17.82% | Low |
| 60 | Presetiawati, I., et.al (2017) (77) | Quasi-experimental | - T2D patients were consecutively recruited from a hospital in West Java. - 77% were female, and 67% were > 55 years. | Adherence | Morisky Medication Adherence Scale with an unclear cut-off value. | 30 | 46.67 | Low |
|  |  |  |  | Control | HbA1c < 7% | 30 | 3.33 | Low |
| 61 | Putra, G.M, et.al (2023) (78) | Case-control | - T2D patients without osteoporosis (control) were consecutively recruited from a hospital in Bali from February to April 2022. - 63.6% were female. | Control | HbA1c < 7% via venous puncture. | 22 | 54.54 | Low |
| 62 | Rahayu, F.P., et.al (2021) (79) | Cross-sectional | - T2D patients from 3 PHCs in Indonesia, who were registered in the Prolanis program and used anti-diabetic medication, were consecutively recruited. - 69.4% were > 54 years old, and 76.9% were female. | Adherence | Medication Adherence to Treatment (MAT) questionnaire with a cut-off value of the mean. | 78 | 65.38 | Medium |
| 63 | Rahem, A., et.al (2021) (80) | Cross-sectional | - T2D patients who routinely did check-ups in a PHC in East Java were consecutively recruited. - 73.2% were female, and >50 aged >50 years. | Adherence | Unclear method. | 425 | 45.41 | Low |
| 64 | Rahem, A., et.al (2023) (81) | Cross-sectional | - T2D patients who visited a PHC in East Java and who were consuming antidiabetic drugs were consecutively recruited. | Adherence | Unclear method. | 272 | 63.97 | Low |
| 65 | Rasinah, N., et.al (2016) (82) | Cross-sectional | - T2D patients were consecutively recruited from a PHC in the Special Region of Yogyakarta between August and October 2015. - 74.8% were female, and 60.2% were < 60 years. | Adherence | MMAS-8 with a cut-off value of > 5. | 123 | 43.90 | Low |
| 66 | Romadhlon, D.S., et.al (2022) (83) | Cross-sectional | - T2D patients enrolled in a diabetes management center in East Java were consecutively recruited. - 64.5% were female, and the mean age was 53.26 ± 7.1 | Treatment | Self-reported diabetes treatment. | 200 | 89.00 | Low |
| 67 | Romadhlon, D.S., et.al (2025) (84) | RCT | - T2D patients having HbA1c level >7% were consecutively recruited from a diabetes management center in East Java between December 2022 and July 2023. - 68% were female | Treatment | Self-reported diabetes treatment | 84 | 100.00 | Low |
| 68 | Rosaria, E., et.al (2020) (85) | Cross-sectional | - T2D patients at a hospital in South Sulawesi were consecutively recruited in 2020. - 69.2% were female, and the mean age was 59.23. | Control | HbA1c <7% | 52 | 40.38 | Low |
| 69 | Riesvi, W.A., et.al (2019) (86) | Cross-sectional | - T2D patients who were prescribed a combination therapy were consecutively recruited from a health center in East Java. - 80.9% were female, and 87.2% were > 49 years old. | Adherence | A questionnaire on the information about the accuracy of duration, interval, and method of drug administration. | 47 | 100.00 | Low |
| 70 | Risdahiyanti, et.al (2020) (87) | Case-control | - Patients without CHD (control) were consecutively recruited from a hospital in Central Java. - 73% were female, and 57% were > 40 years. | Diagnosis | Medical record. | 49 | 22.44 | Low |
| 71 | Santosa, W.R.B, et.al (2024) (88) | Cross-sectional | - T2D patients aged 36 – 65 years were consecutively recruited from a PHC in East Java. - 56% were female, and 75% were 46 – 65 years. | Awareness | Diabetes Knowledge Questionnaire with the cut-off value of 55% to define good awareness. | 92 | 61.96 | Low |
| 72 | Sari, M.I., et.al (2019) (89) | Cross-sectional | - T2D patients were consecutively recruited from a hospital in South Sulawesi. - 58.4% were female, and the mean age was 57.77. | Control | HbA1c < 7% | 89 | 28.09 | Medium |
| 73 | Sauriasari and Sakti (2018) (90) | Quasi-experimental | - T2D patients who were prescribed oral antidiabetic drugs were consecutively recruited from a health center in the Jakarta Special Region from March to June 2017. - 76% were female, and the mean age was 55 years. | Control | HbA1c < 7% via finger-prick blood. | 30 | 23.33 | Low |
| 74 | Siregar, F.A., et.al (2023) (91) | Cross-sectional | - The general population of 6 districts in a city in North Sumatra was consecutively recruited. - 91.7% were female, and the median age was 49.76 years. | True Prevalence | OGTT with a cut-off value of >125 mg/dL. | 300 | 11.33 | Low |
| 75 | Soetedjo, N.N.M., et.al (2018) | Cross-sectional | - T2D patients were consecutively recruited from a hospital in West Java between December 2013 and June 2016. - 63.9% were female, and the median age was 59 (53 – 65). | Treatment | Self-reported diabetes medication. | 783 | 91.95 | Low |
|  |  |  |  | Control | HbA1c <7% | 783 | 28.01 | Low |
| 76 | Srikartika, V.M., et.al (2016) (92) | Cross-sectional | - All T2D patients who visited an outpatient clinic in South Kalimantan were recruited between December 2014 and March 2015. - 54.2% were female, and 46.3% were > 55 years. | Adherence | MMAS-8 with a cut-off value of > 5 | 48 | 72.92 | Low |
| 77 | Subekti, I., et.al (2017) (93) | Cross-sectional | - T2D patients were consecutively recruited from a hospital in the Jakarta Special Region. - 57.2% were female, and the median age was 60 (47 – 86). | Treatment | Self-reported diabetes medication. | 299 | 99.67 | Low |
| 78 | Suprapti, B., et.al (2023) (94) | Cross-sectional | - T2D patients who were treated at a secondary referral hospital in East Java were consecutively recruited. - 46.7% were female, and the median age was 61 (28 – 87). | Adherence | Brief Medication Questionnaire. | 321 | 16.51 | Low |
|  |  |  |  | Control | HbA1c < 7% | 304 | 65.13 | Low |
| 79 | Tarigan and Megawati (2024) (95) | Cross-sectional | - People who visited a community health post in North Sumatra were consecutively recruited. - 76% were female, and 52% were < 45 years. | True Prevalence | FBG > 125 mg/dL | 412 | 20.15 | Low |
| 80 | Turana, Y., et.al (2020) (96) | Cross-sectional | - General population aged >59 without physical or mental health conditions and olfactory disability were consecutively recruited from the Jakarta Special Region. - 64.7% were female, and the mean age was 67.4. | True Prevalence | Self-reported diabetes diagnosis, being treated with diabetes medication, or having a plasma glucose level > 6.9 mmol. | 470 | 20.12 | Low |
| 81 | Ulfah, N.H., et.al (2022) (97) | Cross-sectional | - T2D patients registered in 2 PHCs in a city in East Java were randomly chosen. - 77.8% were female, and 72% were middle-aged and elderly people. | Awareness | Knowledge about diabetes with an unclear questionnaire. | 158 | 56.33 | Low |
|  |  |  |  | Treatment | Self-report any anti-diabetic medication use | 158 | 94.30 | Low |
| 82 | Veryanti, P.R., et.al (2023) (98) | Cross-sectional | - T2D patients were consecutively recruited from five general hospitals in the Jakarta Special Region between 2021 and 2023. - 57.7% were female, and 62.3% were < 60 years old. | Control | HbA1c < 7% from the medical record. | 501 | 71.01 | Medium |
| 83 | Wati, N.S., et.al (2021) (99) | Cross-sectional | - The general population who had been diagnosed with T2D for at least 3 months was consecutively recruited. - 49% were < 50 years, and 53% were female. | Adherence | Self-reported adherence | 264 | 90.53 | Low |
|  |  |  |  | Control | Self-reported HbA1c < 7% | 264 | 39.77 | Low |
| 84 | Wibowo, M.I.N.A., et.al (2020) (100) | Cross-sectional | - T2D patients were consecutively recruited from a PHC in Central Java. - 85% were women, and 71% were > 60 years old. | Adherence | MARS-5 with a cut-off value of > 5. | 66 | 27.27 | Low |
| 85 | Wijayanti, E.P., et.al (2020) (101) | Cross-sectional | - T2D patients who take oral antidiabetic drugs were consecutively recruited from a PHC in East Java. | Adherence | Unclear method | 58 | 100 | Low |
| 86 | Wulandari, N., et.al (2020) (102) | Cross-sectional | - T2D patients who were on at least 2 diabetic medications and had visited PHC at least twice in the past 6 months were consecutively recruited from 6 PHCs in Jakarta in 2019. - 67.8% were female, and 51.7% were > 60 years old. | Control | HbA1c < 7% | 143 | 24.48 | Low |
| 87 | Wulan, W.R., et.al (2023) (103) | Cross-sectional | - Prolanis participants were consecutively recruited from 5 PHCs in Central Java. - 72% were female, and 54.14% were < 65 years old. | Diagnosed | Self-reported doctor diagnosis. | 157 | 63.70 | Low |
| 88 | Wungu, C.D.K., et.al (2024) (104) | Cross-sectional | - The general population was consecutively recruited in a village in East Java. - 78.9% were female, and 52.22% were > 59 years old. | Diagnosed | Self-reported doctor diagnosis | 90 | 33.33 | Low |
| 89 | Yasin, N.M., et.al (2024) (105) | Cross-sectional | - T2D patients were consecutively recruited from 8 PHCs in Yogyakarta Special Region between January and May 2024. - 65.80% were female, and 89% aged > 54 years. | Awareness | Diabetes Knowledge Questionnaire with a cut-off value of > 9 points. | 155 | 85.81 | Low |
|  |  |  |  | Treatment | Self-report of any anti-diabetic medication. | 155 | 100.00 | Low |
| 90 | Yasin, N.M., et.al (2024) (106) | Cross-sectional | - T2D patients using at least one oral antidiabetic medication were consecutively recruited from 5 PHCs in the Special Region of Yogyakarta. - 76.9% were female, and 98.1% were aged > 44 years old. | Awareness | Diabetes Knowledge Questionnaire with a cut-off value of > 5 points. | 104 | 59.61 | Low |
|  |  |  |  | Adherence | Morisky, Green, and Levine Medication Adherence Questionnaire with a cut-off value of < 3 for adherence. | 104 | 74.01 | Low |
| 91 | Yulianti, T., et.al (2020) (107) | Cross-sectional | - Outpatient T2D patients using antidiabetic medication for at least 1 month were consecutively recruited from a hospital in Central Java. - 61.2% were < 60 years old, and 52.9% were female. | Adherence | MARS-5 with a cut-off value of > 24. | 85 | 43.53 | Low |
| 92 | Yunir, E., et.al (2023) (108) | Cross-sectional | - Outpatient T2D patients were consecutively recruited from a hospital in Jakarta Special Region between October 2019 and 2020. - 62.9% were female, and median age was 61 (54 – 66). | Control | HbA1c < 7% | 291 | 37.80 | Low |
| 93 | Zairina, E., et.al (2022) (109) | Cross-sectional | - T2D patients were consecutively recruited from 63 PHCs in East Java between April and September 2019. - 75.6% were female, and the mean age was 61.31 | Adherence | ARMS with a cut-off value of < 13 for adherence. | 266 | 30.45 | Low |
| 94 | National Health Survey 2023 (110) | Cross-sectional | - The general population aged > 15 years or older was randomly selected using a multi-stage random sampling method in Indonesia in 2023. | Prevalence | Blood glucose measurement with FBG > 125 mg/dL or OGTT > 199 mg/dL |  | 11.7 (11.1 – 12.4) | High |
|  |  |  |  | Screening | Self-reported blood glucose measurement in the past 1 year. | 638,178 | 33.6 | High |
|  |  |  |  | Diagnosed diabetes | Self-reported diabetes diagnosis |  | 2.2 (2.2 – 2.3) | High |
|  |  |  |  | Treatment | Self-reported use of any glucose-lowering medication. | 14,593 | 92.00 | High |
|  |  |  |  | Adherence | Self-reported adherence | 13,891 | 89.50 (88.7 – 90.3) | High |
|  |  |  |  | Control | HbA1c <7% |  | Men: 18.6 (15.2 – 22.7)  Women: 20.5 (15.5 – 26.6) | High |

# **Details of Risk Bias Assessment**

The risk of bias assessment was performed using the Johanna Briggs Institute Critical Appraisal Checklist for prevalence studies (111), which consists of the following items:

Table D 1 JBI Checklist

| **No** | **Items** | **Judgement** |
| --- | --- | --- |
| 1. | Was the sample frame appropriate to address the target population? | - Yes, if samples were recruited from at least two different regions, or two different provinces within the same region, or two different cities within the same province. - No, if the samples were from a single center. - Unclear if the geographical areas of the samples were not clear. |
| 2. | Were the participants recruited in an appropriate way? | - Yes, if the study employed a random sampling or total sampling method. - No, if the study employed a consecutive sampling method. - Unclear if the study employed a random sampling method, but the method of randomization was not clear. |
| 3. | Was the sample size adequate? | - Yes, if the sample size ≥ 500. - No, if the sample size < 500. |
| 4. | Were the study subjects and setting described in detail? | - Yes, if the study described information about sex and gender. - No, if the study did not describe information about sex and gender, or only described one of the characteristics. |
| 5. | Was the data analysis conducted with sufficient coverage of the identified sample? | - Yes, if the samples had a balanced proportion of sex and age distribution. - No, if the samples did not have a balanced proportion of sex and age distribution. - Unclear if the study subjects were not described in detail; therefore, judgment on sufficient coverage could not be made. |
| 6. | Were valid methods used for the identification of the condition? | - Yes, if the study used measurement tools that have been validated among the Indonesian population. - No, if the study used measurement tools that have not been validated among the Indonesian population. - Unclear if the study claimed that the measurement tools have been validated, but unclear if it was validated among the Indonesian population or not. |
| 7. | Was the condition measured in a standard, reliable way for all participants? | - Yes, if there were a standard operation procedure for the data collection and measurement method. - No, if there were no information about the operation procedure for the data collection and measurement method. |
| 8. | Was there appropriate statistical analysis? | - Yes, if the prevalence was presented with a confidence interval. - No, if there was no confidence interval. |
| 9. | Was the response rate adequate, and if not, was the low response rate managed appropriately? | - Yes, if there was information about non-response during the sampling method, and an appropriate measure to handle the non-response was performed. - No if there was no information about the non-response during the sampling method. - Unclear if there was information about the non-response during the sampling recruitment, but the method of handling was not clear. - Not applicable if the study used a total sampling method. |

**Table D 2 Risk of bias assessment of the included evidence**

| **No** | **Authors** | **Touchpoints** | **Joanna Briggs Institute Critical Appraisal Checklist** | | | | | | | | |  |
| --- | --- | --- | --- | --- | --- | --- | --- | --- | --- | --- | --- | --- |
|  |  |  | **1** | **2** | **3** | **4** | **5** | **6** | **7** | **8** | **9** | **Total Score** |
| 1 | Aditama, L., et.al (2020) (19) | Adherence | No | No | No | Yes | No | Yes | Unclear | No | No | 2 |
| 2 | Alfian S.D., et.al (2016) (20) | Adherence | No | No | No | Yes | No | No | Unclear | No | No | 1 |
| 3 | Akrom, A., et.al (2019) (21) | Adherence | No | No | No | Yes | No | Yes | Unclear | No | No | 2 |
| 4 | Amelia and Harahap (2019) (22) | Diagnosis | No | No | No | Yes | Yes | Yes | Unclear | No | No | 3 |
| 5 | Amelia, R., et.al (2024) (23) | Control | No | No | No | Yes | No | Yes | Yes | No | No | 3 |
| 6 | Amiruddin, R., et.al (2021) (24) | Treatment | No | No | No | Yes | No | Unclear | Yes | No | No | 2 |
| 7 | Ananda, R.D.S, et.al (2019) (25) | Adherence | No | Unclear | No | Yes | No | Unclear | Unclear | No | No | 1 |
| 8 | Ardiany, D., et.al (2022) (26) | Control | No | No | No | Yes | Unclear | Yes | Yes | No | No | 3 |
| 9 | Arifin, B., et.al (2019) (27) | Treatment | Yes | No | Yes | Yes | No | No | Yes | No | No | 4 |
| 10 | Arifin, B., et.al (2019) (28) | Treatment | Yes | No | Yes | Yes | Yes | No | Yes | No | No | 5 |
| 11 | Arliny, Y., et.al (2022) (29) | Control | No | No | No | Yes | No | No | Unclear | No | No | 1 |
| 12 | Asril, N.M., et.al (2020) (30) | Treatment | No | No | No | Yes | Unclear | Yes | Unclear | No | No | 2 |
| 13 | Azam, M., et.al (2023) (31) | Treatment | Yes | Yes | Yes | Yes | No | Yes | Yes | No | No | 6 |
|  |  | Adherence | Yes | Yes | Yes | Yes | No | Yes | Yes | No | No | 6 |
| 14 | Azmiardi, A., et.al (2023) | Adherence | Yes | Yes | Yes | Yes | No | Yes | Yes | No | No | 6 |
| 15 | Bhaskara, G., et.al (2022) (32) | Control | No | No | No | Yes | No | Yes | Unclear | No | No | 2 |
| 16 | Darmada, and Wulandari (2020) (33) | Adherence | No | No | No | Yes | No | Unclear | Unclear | No | No | 1 |
| 17 | Dewanti, L., et.al (2024) (34) | Control | No | No | No | Yes | No | Yes | Unclear | No | No | 2 |
| 18 | Dwiyatna, S., et.al (2024) (35) | Awareness | No | No | No | Yes | No | Yes | Unclear | No | No | 2 |
|  |  | Adherence | No | No | No | Yes | No | Yes | Unclear | No | No | 2 |
|  |  | Control | No | No | No | Yes | No | Unclear | Unclear | No | No | 1 |
| 19 | Elnaem, M.H., et.al (2025) (36) | Treatment | No | No | No | Yes | No | Unclear | Yes | No | No | 2 |
|  |  | Adherence | No | No | No | Yes | No | Unclear | Yes | No | No | 2 |
| 20 | Faridah, I.N., et.al (2022) (37) | Adherence | No | No | No | Yes | No | Yes | Unclear | No | No | 2 |
| 21 | Fibriana, A.I., et.al (2020) (38) | Adherence | No | No | No | Yes | No | No | Unclear | No | No | 1 |
| 22 | Fritz, M., et.al (2024) (39) | Screening | No | Yes | Yes | Yes | No | Unclear | Unclear | No | No | 3 |
|  |  | Diagnosed | No | Yes | Yes | Yes | No | Unclear | Unclear | No | No | 3 |
| 23 | Fritz, M., et.al (2024) (40) | Screening | No | Yes | Yes | Yes | No | Yes | Yes | No | No | 5 |
| 24 | Handayani, O.W.K., et.al (2019) (41) | Adherence | No | Yes | No | No | Unclear | Unclear | Unclear | No | No | 1 |
| 25 | Hendrianingtyas, M., et. al (2020) (42) | Control | No | No | No | Yes | No | Yes | Unclear | No | No | 2 |
| 26 | Herwana and Febiana (2025) (43) | Treatment | No | No | No | Yes | No | Unclear | Unclear | No | No | 1 |
|  |  | Control | No | No | No | Yes | No | Yes | Unclear | No | No | 2 |
| 27 | Hidayat, B., et.al (2022) (44) | Treatment | Yes | Yes | Yes | Yes | No | Yes | Yes | No | N/A | 7 |
| 28 | Indrayanti, S., et.al (2019) (45) | Diagnosed | No | No | No | Yes | Yes | Yes | Unclear | No | Unclear | 3 |
| 29 | Jasmine, N.S., et.al (2020) (46) | Awareness | No | No | No | Yes | Yes | Unclear | Unclear | No | No | 2 |
|  |  | Adherence | No | No | No | Yes | Yes | Unclear | Unclear | No | No | 2 |
| 30 | Jaya, M.K.A., et.al (2024) (47) | Control | No | No | No | Yes | No | Unclear | Unclear | No | No | 1 |
| 31 | Jaya, M.K.A., et.al (2025) (48) | Control | No | No | No | Yes | No | Yes | Yes | No | No | 3 |
| 32 | Julaiha, S., et.al (2019) (49) | Adherence | No | Yes | No | Yes | No | Unclear | Unclear | No | N/A | 3 |
| 33 | Kresnowati, et.al (2025) (50) | Screening | Yes | Yes | Yes | No | Yes | Yes | Yes | No | N/A | 7 |
| 34 | Kristina, S.A., etl.al (2020) (51) | Treatment | Yes | Yes | Yes | Yes | Yes | Yes | Yes | No | No | 7 |
|  |  | Control | Yes | Yes | Yes | Yes | Yes | Unclear | Yes | No | No | 6 |
| 35 | Kristina, S.A., etl.al (2021) (52) | Awareness | No | Unclear | Yes | Yes | No | Yes | Unclear | No | No | 3 |
|  |  | Diagnosis | No | Unclear | Yes | Yes | No | Yes | Unclear | No | No | 3 |
| 36 | Kristanti, D., et.al (2021) (53) | True Prevalence | No | Unclear | Yes | Yes | No | Yes | Unclear | No | No | 3 |
| 37 | Kurnia, A.D., et.al (2017) (54) | Awareness | No | Yes | No | Yes | No | Unclear | Unclear | No | No | 2 |
|  |  | Treatment | No | Yes | No | Yes | No | Unclear | Unclear | No | No | 2 |
| 38 | Kurnia, A.D., et.al (2022) (55) | Treatment | No | No | No | Yes | No | Yes | Unclear | No | No | 2 |
| 39 | Kurniati, I., et.al (2024) (56) | Control | No | No | No | Yes | Unclear | Yes | Yes | No | No | 3 |
| 40 | Kurniawan, F., et.al (2024) (57) | True Prevalence | Yes | Yes | Yes | Yes | Yes | Yes | Yes | Yes | Unclear | 8 |
|  |  | Diagnosis | Yes | Yes | Yes | Yes | Yes | Yes | Yes | Yes | Unclear | 8 |
| 41 | Lim, L.L., et.al (2023) (58) | Control | Unclear | No | Yes | No | Unclear | Yes | Yes | Yes | No | 4 |
| 42 | Maharani, A., et.al (2019) (59) | True Prevalence | No | Yes | Yes | Yes | No | Yes | Yes | No | N/A | 6 |
| 43 | Makkulawu, A., et.al (2019) (60) | Adherence | No | No | No | Yes | No | Yes | Unclear | No | No | 2 |
| 44 | Malini, H., et.al (2022) (61) | Adherence | No | No | Yes | Yes | No | Unclear | Unclear | No | No | 2 |
| 45 | Masuroh, N.L, et.al (2021) (62) | Awareness | No | Yes | No | Yes | No | Unclear | Unclear | No | No | 2 |
|  |  | Adherence | No | Yes | No | Yes | No | Yes | Unclear | No | No | 3 |
| 46 | Mayasari, D.S., et.al (2021) (63) | Treatment | No | No | No | Yes | Unclear | Yes | Unclear | No | No | 2 |
|  |  | Control | No | No | No | Yes | Unclear | Yes | Unclear | No | No | 2 |
| 47 | Mulyanto, J., et.al (2019) (64) | True Prevalence | Yes | Yes | Yes | Yes | Yes | Yes | Yes | Yes | Unclear | 8 |
|  |  | Screening | Yes | Yes | Yes | Yes | Yes | Yes | Yes | Yes | Unclear | 8 |
|  |  | Treatment | Yes | Yes | Yes | Yes | Yes | Yes | Yes | Yes | Unclear | 8 |
| 48 | Muhammadong, J., et.al (2024) (65) | True Prevalence | No | No | No | Yes | No | Yes | Unclear | No | No | 2 |
| 49 | Nanda, O.D., et.al (2018) (66) | Adherence | No | No | No | Yes | No | Unclear | Unclear | No | No | 1 |
| 50 | Natasya, A., et.al (2018) (67) | Adherence | No | No | No | Yes | No | No | Unclear | No | No | 1 |
|  |  | Control | No | No | No | Yes | No | Yes | Unclear | No | No | 2 |
| 51 | Nazriati, E., et.al (2018) (68) | Awareness | No | Unclear | No | Yes | No | Unclear | Unclear | No | No | 1 |
|  |  | Adherence | No | Unclear | No | Yes | No | Unclear | Unclear | No | No | 1 |
| 52 | Ningsih, O.S., et.al (2024) (69) | Treatment | No | No | No | Yes | Yes | Unclear | Unclear | No | No | 2 |
| 53 | Notariza, K.R., et.al (2021) (70) | Treatment | No | No | No | Yes | No | No | Unclear | No | No | 1 |
| 54 | Nugrahaeni, D.K., et.al (2022) (71) | Treatment | No | No | No | Yes | No | No | Unclear | No | No | 1 |
| 55 | Nugroho, D.B., et.al (2022) (72) | Diagnosed | No | Yes | Yes | Yes | No | Yes | Yes | No | No | 5 |
| 56 | Pratama, I.P.Y, et.al (2019) (73) | Awareness | No | No | No | Yes | No | Yes | Unclear | No | No | 2 |
| 57 | Pratiwi, C., et.al (2022) (74) | Treatment | No | No | No | Yes | Yes | Yes | Yes | No | No | 4 |
| 58 | Pavitasari, A., et.al (2022) (75) | Adherence | No | No | No | Yes | No | No | No | No | No | 1 |
| 59 | Permana, H., et.al (2022) (76) | Treatment | No | No | Yes | Yes | No | No | Yes | No | No | 3 |
|  |  | Control | No | No | No | No | Unclear | Yes | Yes | No | No | 2 |
| 60 | Presetiawati, I., et.al (2017) (77) | Adherence | No | No | No | Yes | No | Unclear | Unclear | No | No | 1 |
|  |  | Control | No | No | No | Yes | No | Unclear | Unclear | No | No | 1 |
| 61 | Putra, G.M, et.al (2023) (78) | Control | No | No | No | No | Unclear | Yes | Unclear | No | No | 1 |
| 62 | Rahayu, F.P., et.al (2021) (79) | Adherence | No | Yes | No | Yes | No | Yes | Yes | No | N/A | 5 |
| 63 | Rahem, A., et.al (2021) (80) | Adherence | No | No | No | Yes | No | No | Unclear | No | No | 1 |
| 64 | Rahem, A., et.al (2023) (81) | Adherence | No | No | No | No | Unclear | No | Unclear | No | No | 0 |
| 65 | Rasinah, N., et.al (2016) (82) | Adherence | Unclear | No | No | Yes | No | Yes | Unclear | No | No | 2 |
| 66 | Romadhlon, D.S., et.al (2022) (83) | Treatment | No | No | No | Yes | No | Unclear | Unclear | No | No | 1 |
| 67 | Romadhlon, D.S., et.al (2025) (84) | Treatment | No | No | No | Yes | No | No | Unclear | No | No | 1 |
| 68 | Rosaria, E., et.al (2020) (85) | Control | No | No | No | No | No | Unclear | Unclear | No | No | 0 |
| 69 | Riesvi, W.A., et.al (2019) (86) | Adherence | No | No | No | Yes | No | No | No | No | No | 1 |
| 70 | Risdahiyanti, et.al (2020) (87) | Diagnosis | No | No | No | Yes | No | Yes | Unclear | No | Unclear | 2 |
| 71 | Santosa, W.R.B, et.al (2024) (88) | Awareness | No | No | No | Yes | Yes | Yes | Unclear | No | No | 3 |
| 72 | Sari, M.I., et.al (2019) (89) | Control | No | No | No | Yes | Yes | Yes | Yes | No | No | 4 |
| 73 | Sauriasari and Sakti (2018) (90) | Control | No | No | No | Yes | No | Yes | Unclear | No | No | 2 |
| 74 | Siregar, F.A., et.al (2023) (91) | True Prevalence | No | No | No | Yes | No | Yes | Unclear | No | No | 2 |
| 75 | Soetedjo, N.N.M., et.al (2018) | Treatment | No | No | Yes | Yes | No | Unclear | Yes | No | No | 3 |
|  |  | Control | No | No | Yes | Yes | No | Unclear | Yes | No | No | 3 |
| 76 | Srikartika, V.M., et.al (2016) (92) | Adherence | No | No | No | Yes | Yes | Unclear | Unclear | No | No | 2 |
| 77 | Subekti, I., et.al (2017) (93) | Treatment | No | No | No | Yes | Yes | Unclear | Unclear | No | No | 2 |
| 78 | Suprapti, B., et.al (2023) (94) | Adherence | No | No | No | Yes | No | Yes | Unclear | No | No | 2 |
|  |  | Control | No | No | No | Yes | No | Yes | Unclear | No | No | 2 |
| 79 | Tarigan and Megawati (2024) (95) | True Prevalence | No | No | No | Yes | No | Yes | Yes | No | No | 3 |
| 80 | Turana, Y., et.al (2020) (96) | True Prevalence | No | No | No | Yes | No | Unclear | Unclear | No | Unclear | 1 |
| 81 | Ulfah, N.H., et.al (2022) (97) | Awareness | No | Yes | No | Yes | No | No | Unclear | No | No | 2 |
|  |  | Treatment | No | Yes | No | Yes | No | Yes | Unclear | No | No | 3 |
| 82 | Veryanti, P.R., et.al (2023) (98) | Control | No | No | Yes | Yes | No | Yes | Yes | No | No | 4 |
| 83 | Wati, N.S., et.al (2021) (99) | Adherence | No | No | No | Yes | Yes | Unclear | Unclear | No | No | 2 |
|  |  | Control | No | No | No | Yes | Yes | No | Unclear | No | No | 2 |
| 84 | Wibowo, M.I.N.A., et.al (2020) (100) | Adherence | No | No | No | Yes | No | Yes | Unclear | No | No | 2 |
| 85 | Wijayanti, E.P., et.al (2020) (101) | Adherence | No | No | No | No | Unclear | No | Unclear | No | No | 0 |
| 86 | Wulandari, N., et.al (2020) (102) | Control | No | No | No | Yes | No | Yes | Yes | No | No | 3 |
| 87 | Wulan, W.R., et.al (2023) (103) | Diagnosed | No | No | No | Yes | No | Yes | Unclear | No | No | 2 |
| 88 | Wungu, C.D.K., et.al (2024) (104) | Diagnosed | No | No | No | Yes | No | Unclear | Yes | No | No | 2 |
| 89 | Yasin, N.M., et.al (2024) (105) | Awareness | No | No | No | Yes | No | Yes | No | No | No | 2 |
|  |  | Treatment | No | No | No | Yes | No | Yes | No | No | No | 2 |
| 90 | Yasin, N.M., et.al (2024) (106) | Awareness | No | No | No | Yes | No | Yes | Yes | No | No | 3 |
|  |  | Adherence | No | No | No | Yes | No | Yes | Yes | No | No | 3 |
| 91 | Yulianti, T., et.al (2020) (107) | Adherence | No | No | No | Yes | No | Yes | Unclear | No | No | 2 |
| 92 | Yunir, E., et.al (2023) (108) | Control | No | No | No | Yes | No | Unclear | Unclear | No | No | 1 |
| 93 | Zairina, E., et.al (2022) (109) | Adherence | No | No | No | Yes | No | Unclear | Unclear | No | No | 1 |
| 94 | National Health Survey 2023 (110) | Prevalence | Yes | Yes | Yes | Yes | Yes | Yes | Yes | Yes | Unclear | 8 |
|  |  | Screening | Yes | Yes | Yes | Yes | Yes | Yes | Yes | Yes | Unclear | 8 |
|  |  | Diagnosed diabetes | Yes | Yes | Yes | Yes | Yes | Yes | Yes | Yes | Unclear | 8 |
|  |  | Treatment | Yes | Yes | Yes | Yes | Yes | Yes | Yes | Yes | Unclear | 8 |
|  |  | Adherence | Yes | Yes | Yes | Yes | Yes | Yes | Yes | Yes | Unclear | 8 |
|  |  | Control | Yes | Yes | Yes | Yes | Yes | Yes | Yes | Yes | Unclear | 8 |

# **Pooled Prevalence Estimates and Subgroups Analysis**

## Subgroup analysis by sample representativeness and settings

Table E 1 Summary of subgroup analysis by sample representativeness and settings

| **Touchpoints** | **Subgroups** | **N. studies** | **Pooled prevalence** | **95%CI** | **I2** | **Prediction interval** |
| --- | --- | --- | --- | --- | --- | --- |
| True diabetes | Pooled | 10 | 13 | 9 – 17 | 96.0; p<0.0001 | 4 – 27 |
|  | Nationally representative studies | 4 | 10 | 7 – 14 | 97.4; p<0.0001 |  |
|  | Regionally representative studies | 0 |  |  |  |  |
|  | Provincially representative studies | 6 | 15 | 9 – 22 | 96.0, p<0.0001 |  |
|  | General population settings | 9 | 12 | 9 – 16 | 95.9; p<0.0001 |  |
|  | Healthcare setting studies | 1 | 20 | 16 – 24 |  |  |
| Awareness | Pooled | 11 | 71 | 54 – 85 | 97.7; p<0.0001 | 16 – 100 |
|  | Nationally representative studies | 0 |  |  |  |  |
|  | Regionally representative studies | 0 |  |  |  |  |
|  | Provincially representative studies | 11 | 71 | 54 – 85 | 97.7; p<0.0001 |  |
|  | General population settings | 2 | 44 | 0 – 100 |  |  |
|  | Healthcare setting studies | 9 | 76 | 60 – 90 | 97.1; p<0.0001 |  |
| Screening | Pooled | 5 | 19 | 0 – 55 | 100.0; p=0 | 0 – 97 |
|  | Nationally representative studies | 3 | 18 | 0 – 55 | 100.0; p=0 |  |
|  | Regionally representative studies | 2 | 21 | 0 – 100 | 100.0; p=0 |  |
|  | Provincially representative studies | 0 |  |  |  |  |
|  | General population settings | 5 | 19 | 0 – 55 | 100.0; p=0 |  |
|  | Healthcare settings | 0 |  |  |  |  |
| Diagnosis | Pooled | 11 | 15 | 6 – 27 | 99.4; p<0.0001 | 0 – 61 |
|  | Nationally representative studies | 3 | 3 | 1 – 6 | 98.1; p<0.0001 |  |
|  | Regionally representative studies | 1 | 7 | 6 – 8 |  |  |
|  | Provincially representative studies | 7 | 25 | 14 – 39 | 97.5; p <0.0001 |  |
|  | General population settings | 7 | 9 | 2 – 20 | 99.5; p<0.0001 |  |
|  | Healthcare settings | 4 | 30 | 10 – 55 | 92.8; p<0.0001 |  |
| Treatment | Pooled | 25 | 89 | 80 - 95 | 100.0; p=0 | 34 – 100 |
|  | Nationally representative studies | 6 | 75 | 28 – 100 | 100.0; p=0 |  |
|  | Regionally representative studies | 1 | 95 | 93 – 96 |  |  |
|  | Provincially representative studies | 17 | 92 | 86 – 97 | 96.0; p<0.0001 |  |
|  | General population settings | 5 | 69 | 86 – 97 | 99.8; p=0 |  |
|  | Healthcare settings | 20 | 92 | 44 – 100 | 99.9; p=0 |  |
| Adherence | Pooled | 36 | 59 | 49 – 69 | 99.1; p=0 | 7 – 100 |
|  | Nationally representative studies | 3 | 68 | 14 – 100 | 99.9; p=0 |  |
|  | Regionally representative studies | 0 |  |  |  |  |
|  | Provincially representative studies | 30 | 58 | 47 – 68 | 97.1; p<0.0001 |  |
|  | General population settings | 9 | 63 | 37 – 86 | 99.7; p=0 |  |
|  | Healthcare settings | 27 | 57 | 46 – 69 | 96.8; p<0.0001 |  |
| Control | Pooled | 29 | 31 | 25 – 36 | 95.7; p<0.0001 | 7 – 62 |
|  | Nationally representative studies | 5 | 25 | 19 – 32 | 88.5; p<0.0001 |  |
|  | Regionally representative studies | 0 |  |  |  |  |
|  | Provincially representative studies | 24 | 32 | 25 – 39 | 95.7; p<0.0001 |  |
|  | General population settings | 4 | 26 | 12 – 42 | 92.2; p=0.526 |  |
|  | Healthcare settings | 25 | 31 | 25 – 36 | 95.7; p<0.0001 |  |


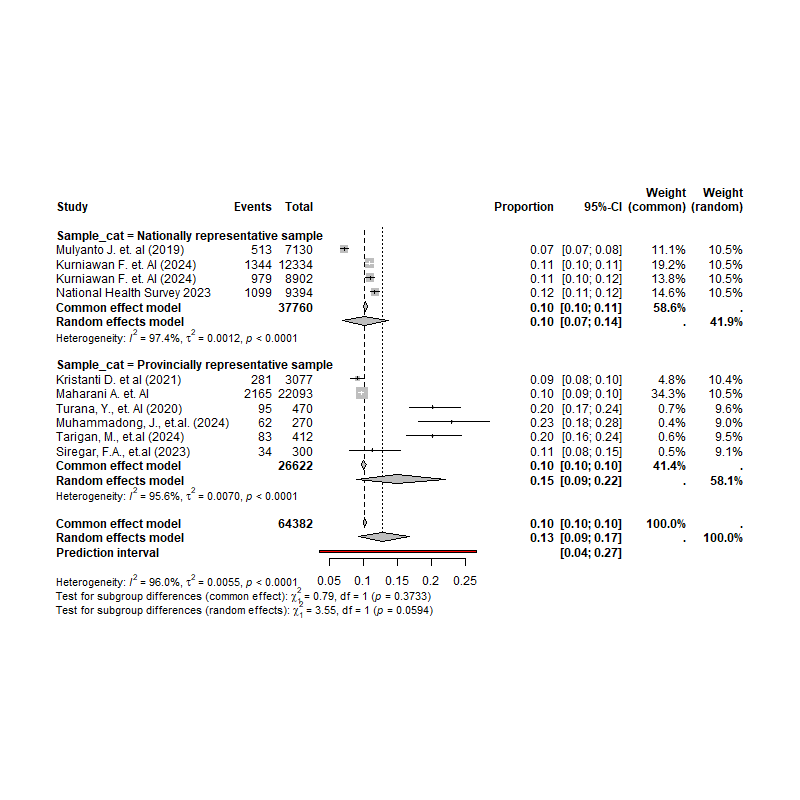


Figure E1 1 Pooled Diabetes Prevalence by Sample Type


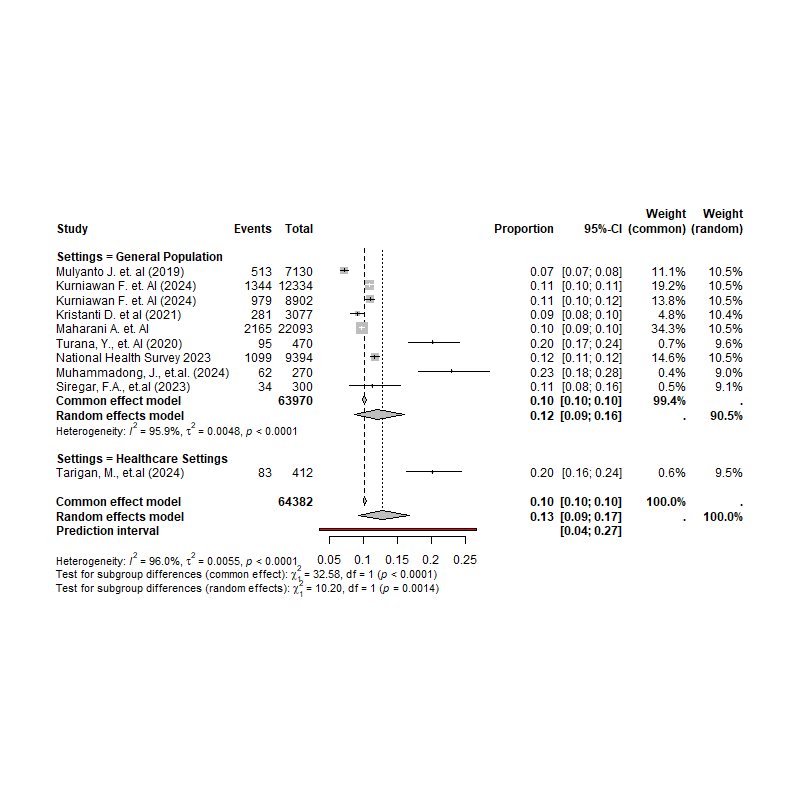


Figure E1 2 Pooled diabetes prevalence by study settings

*
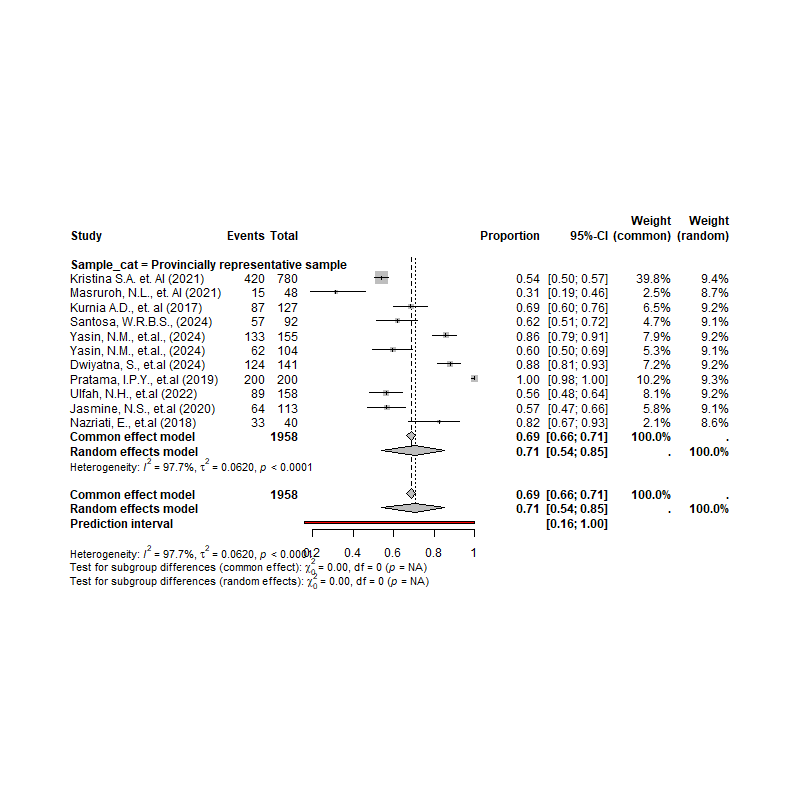
*

Figure E1 3 Pooled good diabetes awareness by sample type


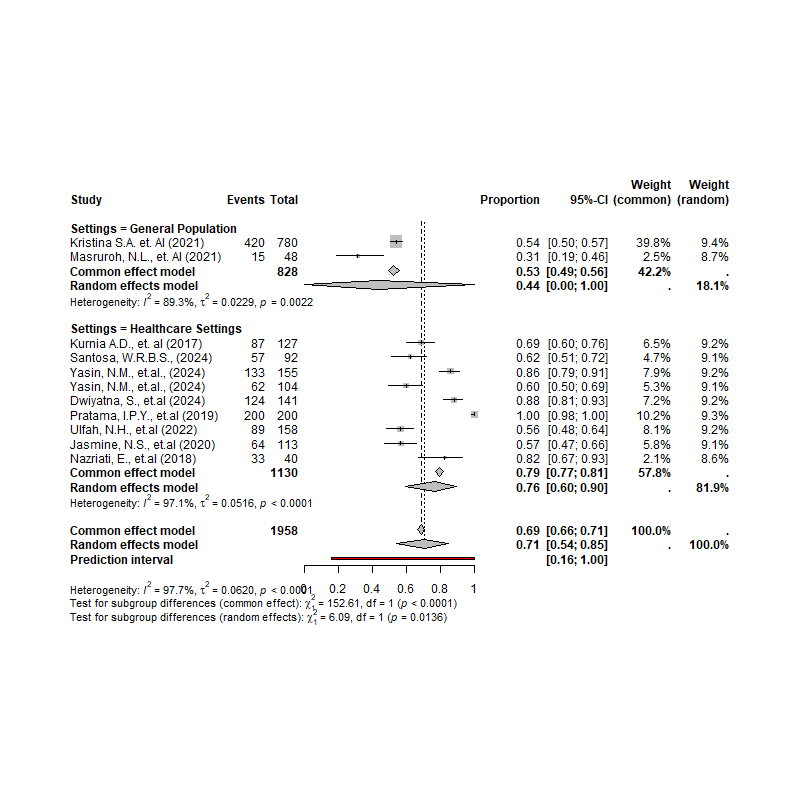


Figure E1 4 Pooled good diabetes awareness by settings


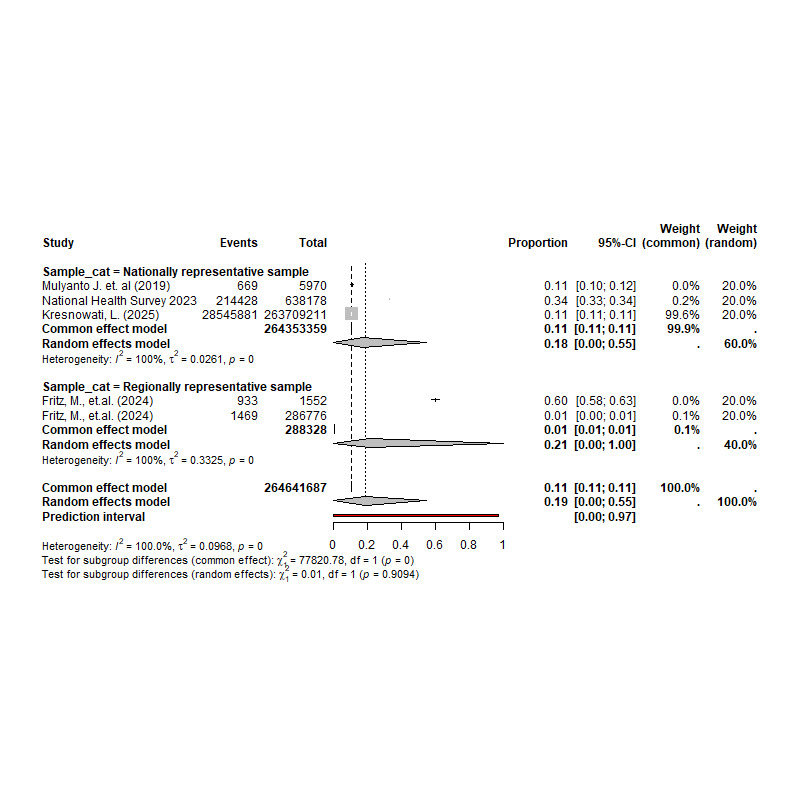


Figure E1 5 Pooled diabetes screening by sample type


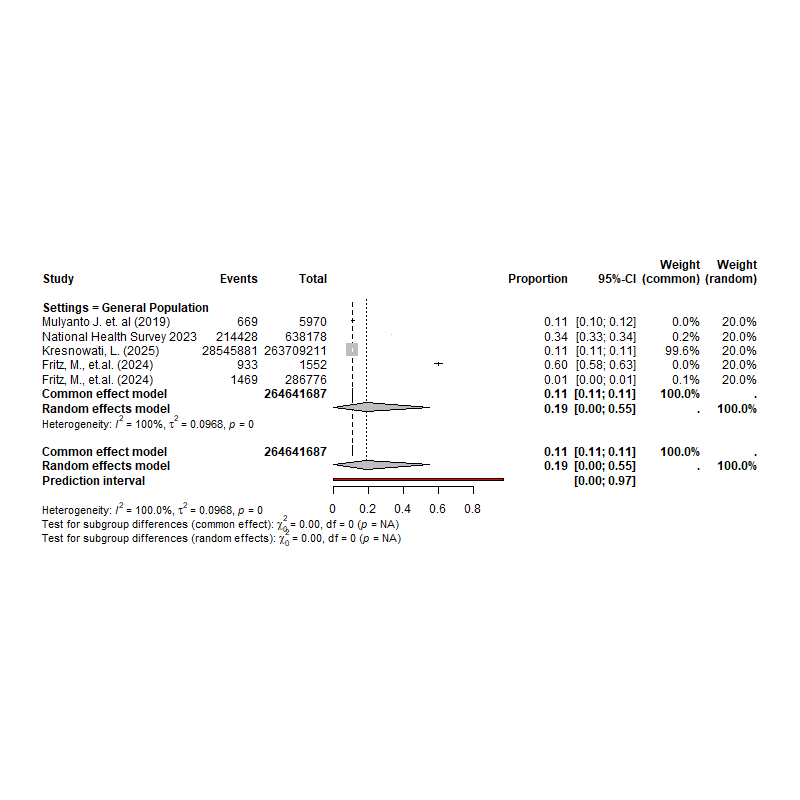


Figure E1 6 Pooled diabetes screening by settings


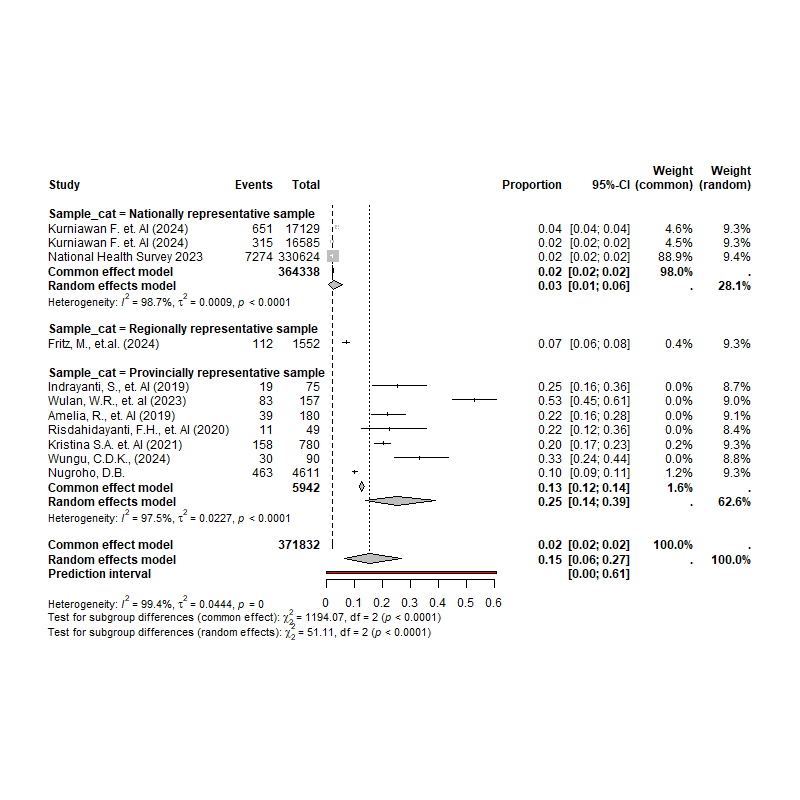


Figure E1 7 Pooled diabetes diagnosis by sample type


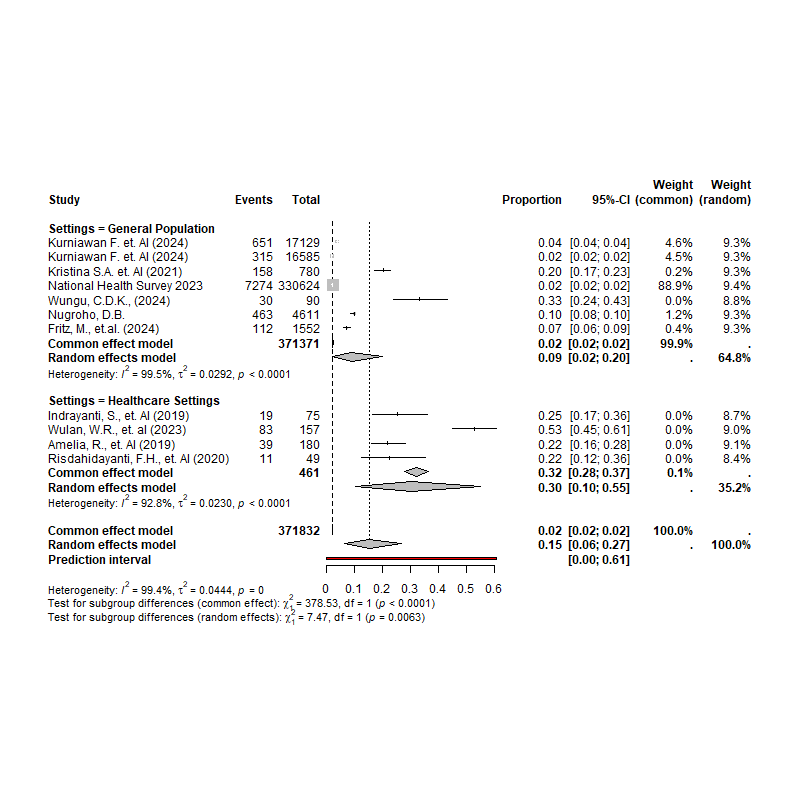


Figure E1 8 Pooled diabetes diagnosis by settings


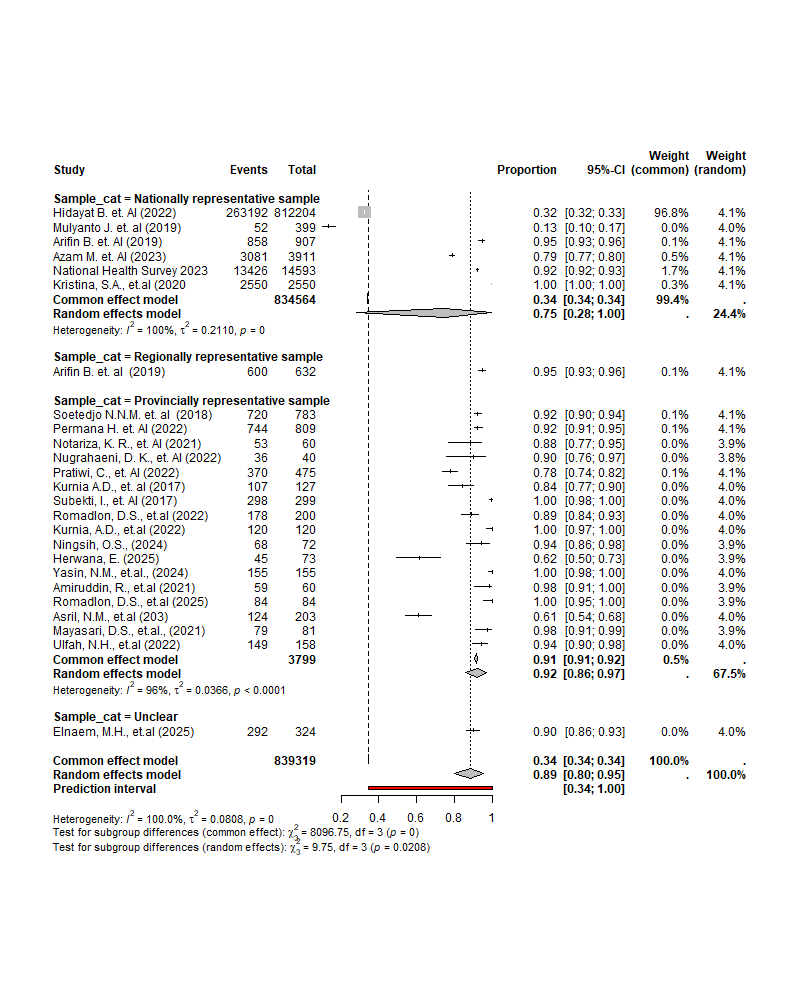


Figure E1 9 Pooled diabetes treatment by sample type


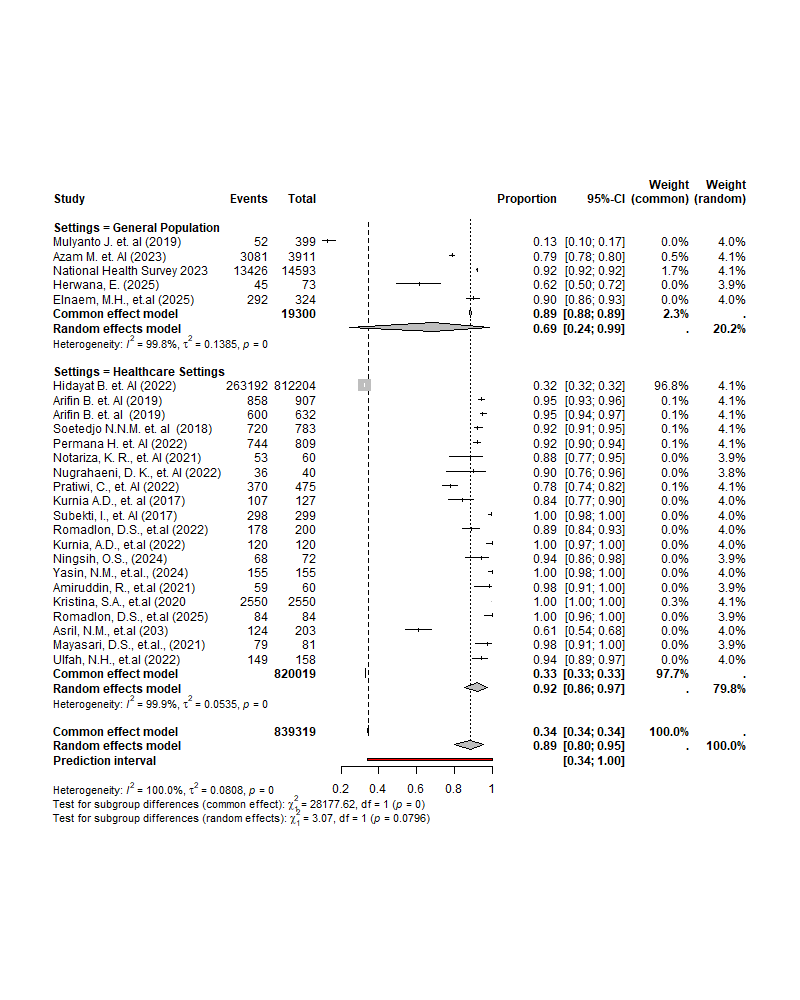


Figure E1 10 Pooled diabetes treatment prevalence by settings


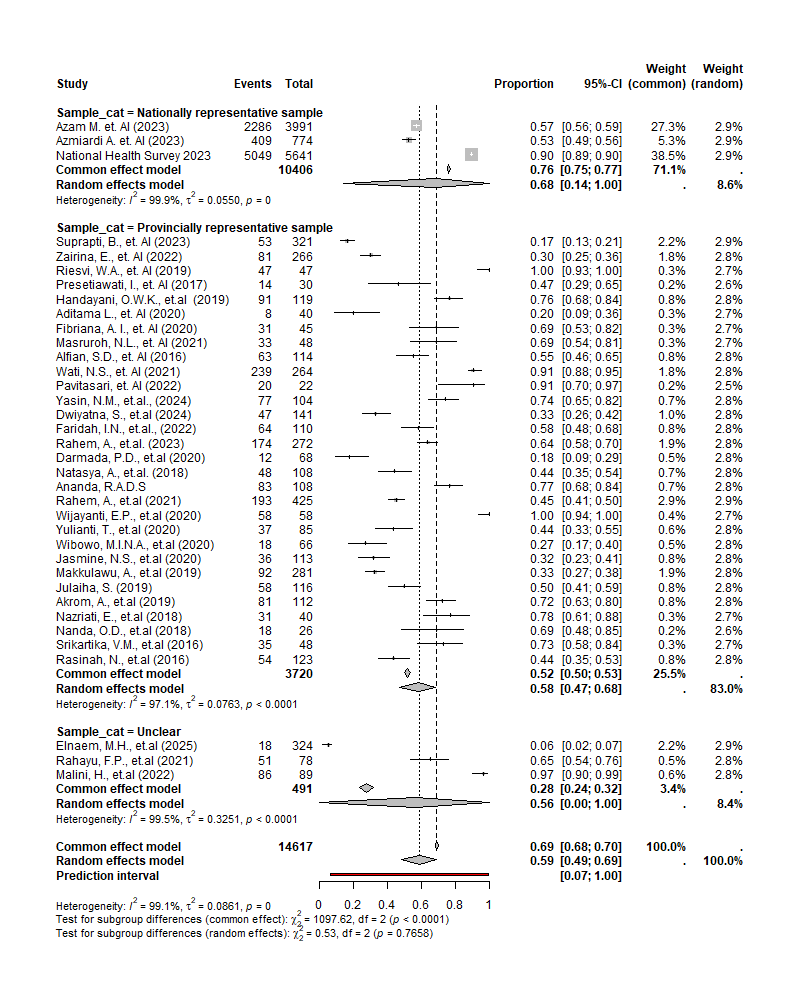


Figure E1 11 Pooled diabetes treatment adherence by sample type


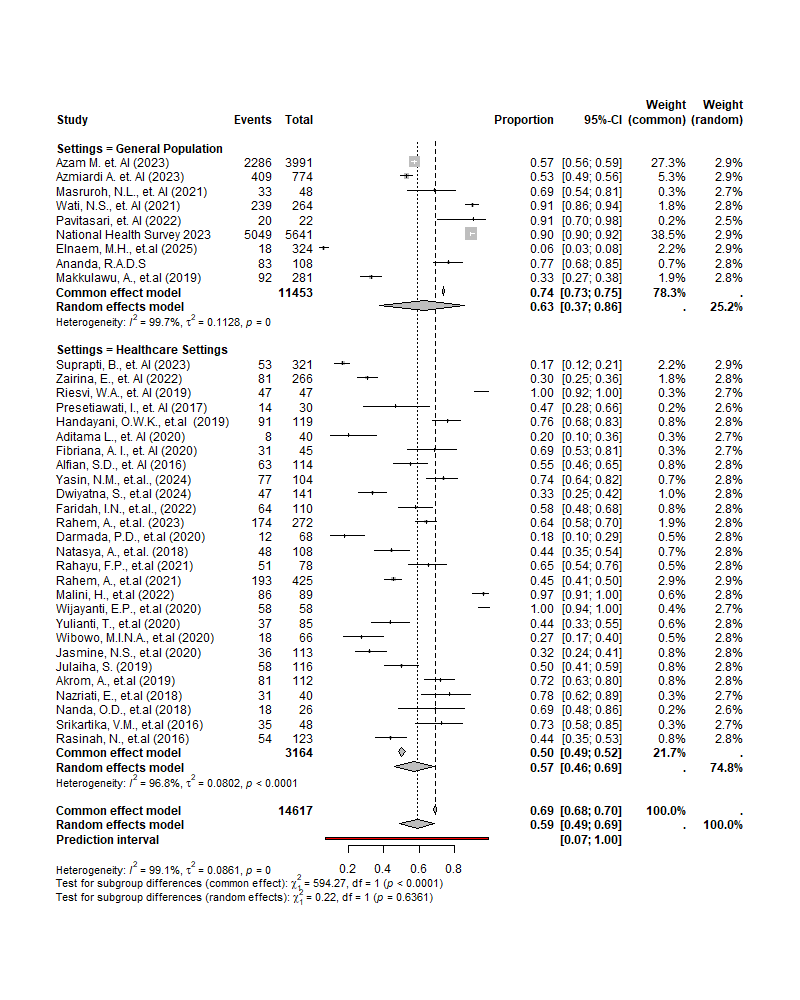


Figure E1 12 Pooled diabetes treatment adherence by settings


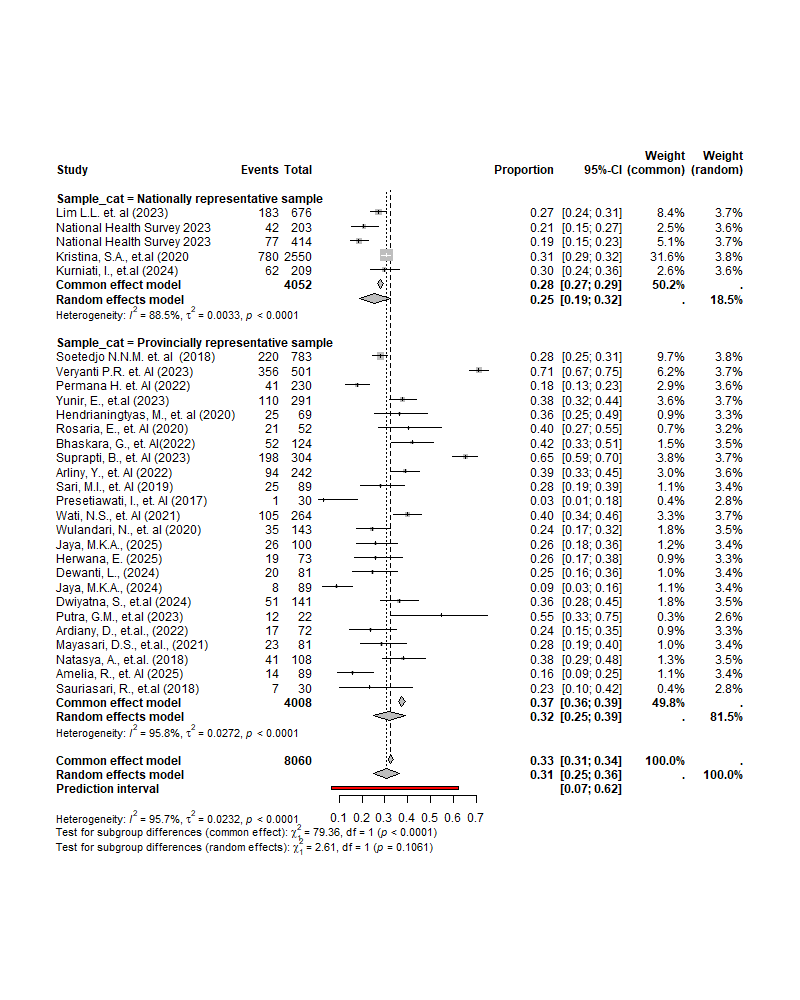


Figure E1 13 Pooled diabetes control by sample type


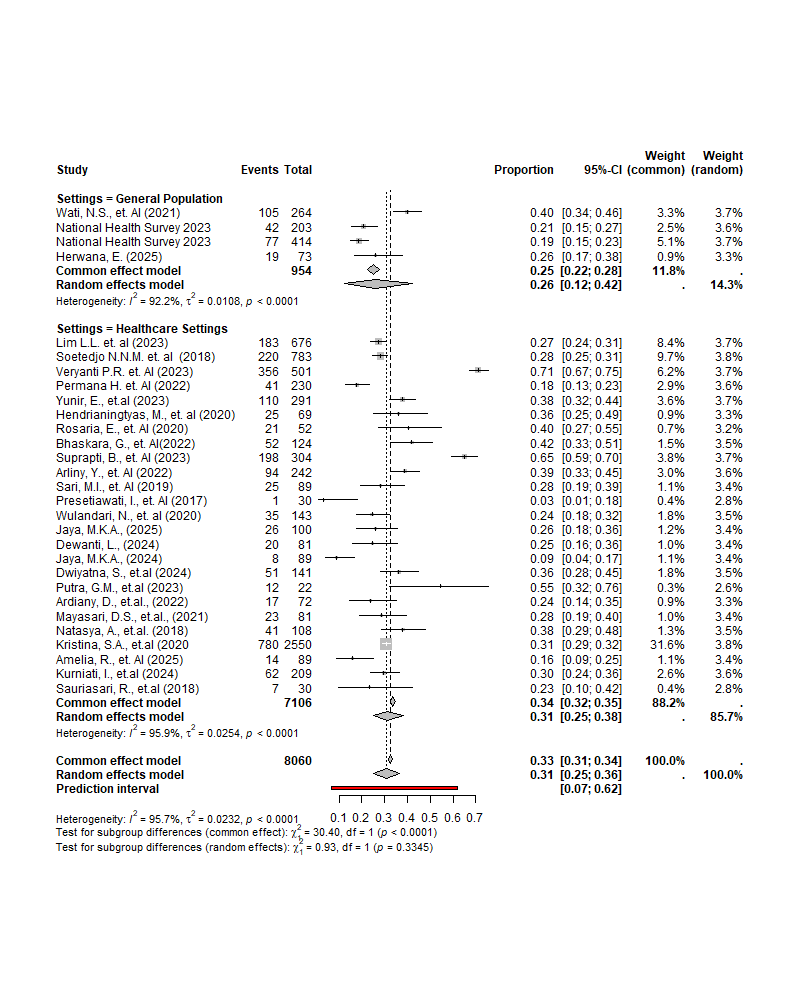


Figure E1 14 Pooled diabetes control by settings

## Subgroup analysis by measurement tool for awareness, screening, treatment, and adherence touchpoints

Table E 2 Summary of subgroup analysis by measurement tool or definition for awareness, screening, treatment, and adherence touchpoints

| **Touchpoints** | **Subgroups** | **N. studies** | **Pooled prevalence** | **95%CI** | **I2** | **Prediction interval** |
| --- | --- | --- | --- | --- | --- | --- |
| Awareness | Pooled | 11 | 71 | 54 – 85 | 97.7; p<0.0001 | 16 – 100 |
|  | Other | 4 | 59 | 49 – 69 | 70.9; p=0.0162 |  |
|  | DKQ24, cut-off unclear | 3 | 85 | 9 – 100 | 98.6; p<0.0001 |  |
|  | DKQ24, cut-off >9 | 2 | 87 | 71 – 97 | 0; p=0.5951 |  |
|  | DKQ24, cut-off >13 | 2 | 47 | 0 – 100 | 91.7; p=0.0005 |  |
| Screening | Pooled | 5 | 19 | 0 – 55 | 100; p=0 | 0 – 97 |
|  | Blood sugar check | 3 | 33 | 0 – 93 | 99.9; p =0 |  |
|  | Risk score | 2 | 4 | 0 – 100 | 100; p = 0 |  |
| Treated | Pooled | 25 | 89 | 80 – 95 | 100.0; p=0 | 34 – 100 |
|  | Any glucose medication | 24 | 90 | 83 – 96 | 99.3; p=0 |  |
|  | 23-day prescription | 1 | 32 | 32 – 32 |  |  |
| Adherence | Pooled | 36 | 59 | 49 – 69 | 99.1; p=0 | 7 – 100 |
|  | Other | 10 | 70 | 44 – 90 | 97.9; p<0.0001 |  |
|  | Single item (yes/no) | 4 | 75 | 38 – 98 | 99.8; p=0 |  |
|  | MMAS, cut-off unclear/ others | 9 | 59 | 38 – 77 | 96.6; p<0.0001 |  |
|  | MMAS, cut-off >5 | 5 | 62 | 45 – 78 | 86.8; p<0.0001 |  |
|  | ARMS, cut-off >13 | 2 | 31 | 15 – 50 | 0; p<0.0001 |  |
|  | MARS, cut off unclear/ others | 2 | 50 | 0 – 100 | 97.2; p<0.0001 |  |
|  | MARS, cut off >24 | 4 | 29 | 1 – 72 | 98.1’ p<0.0001 |  |


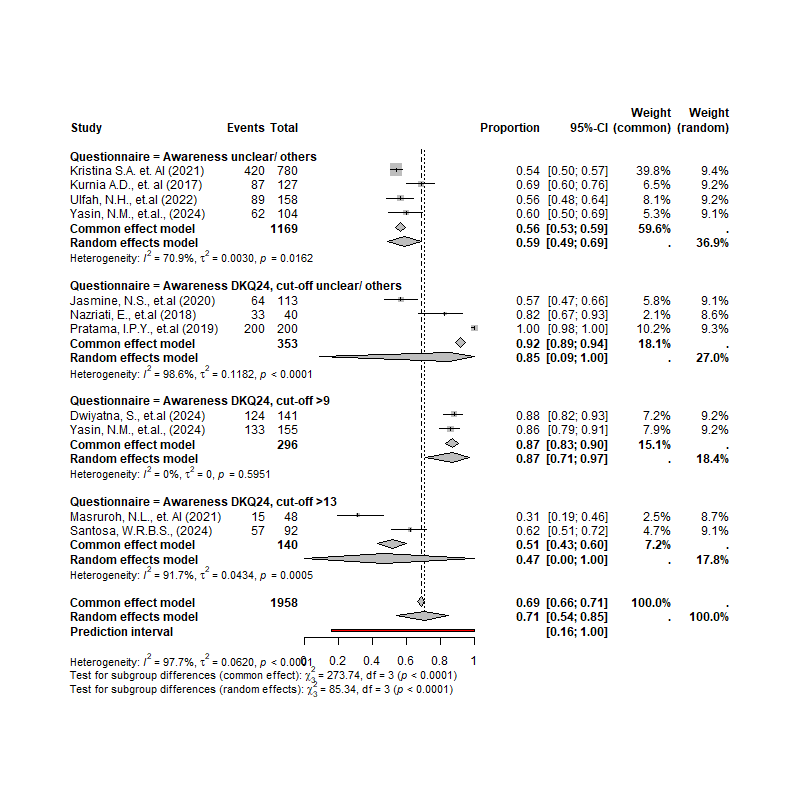


Figure E2 1 Pooled good diabetes awareness by measurement tools


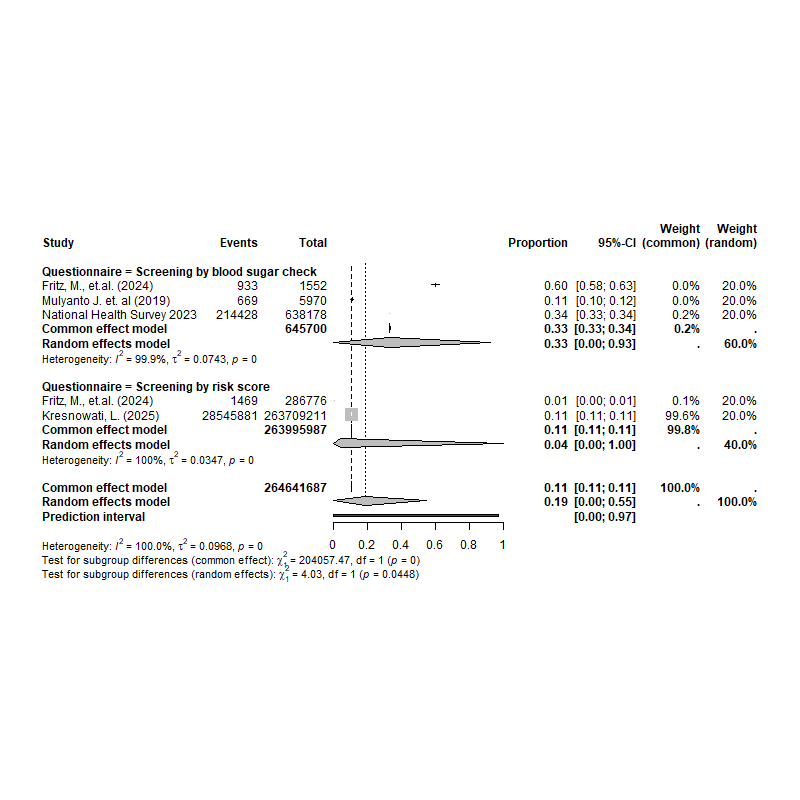


Figure E2 2 Pooled diabetes screening by screening type


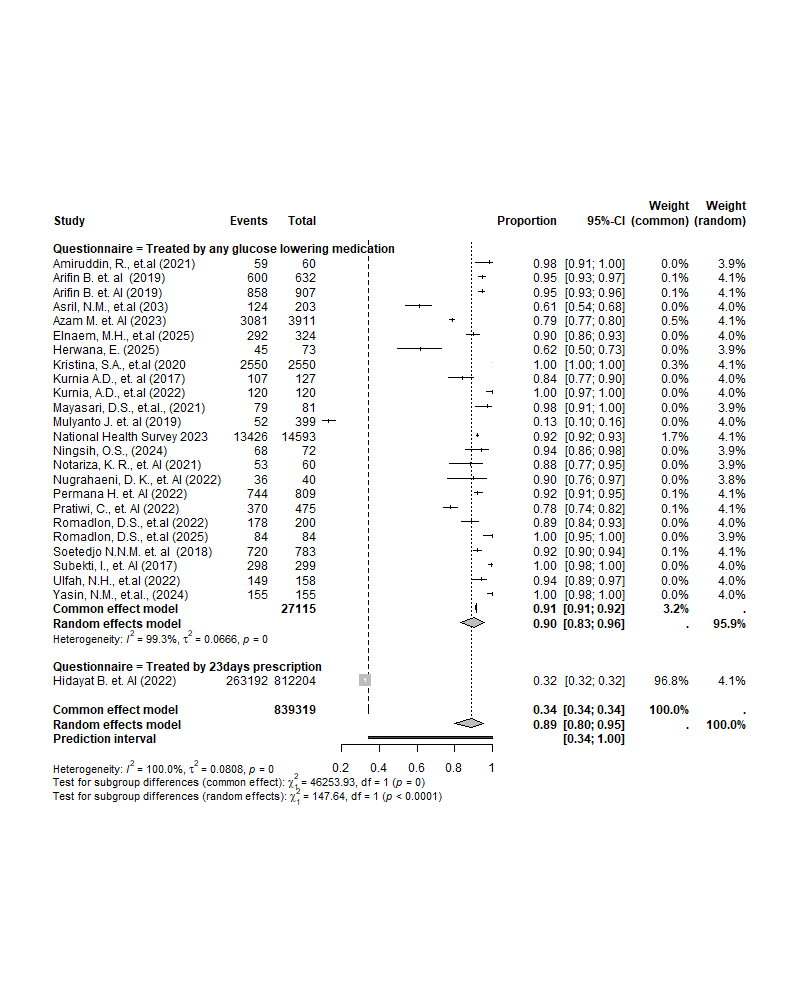


Figure E2 3 Pooled diabetes treatment by definition


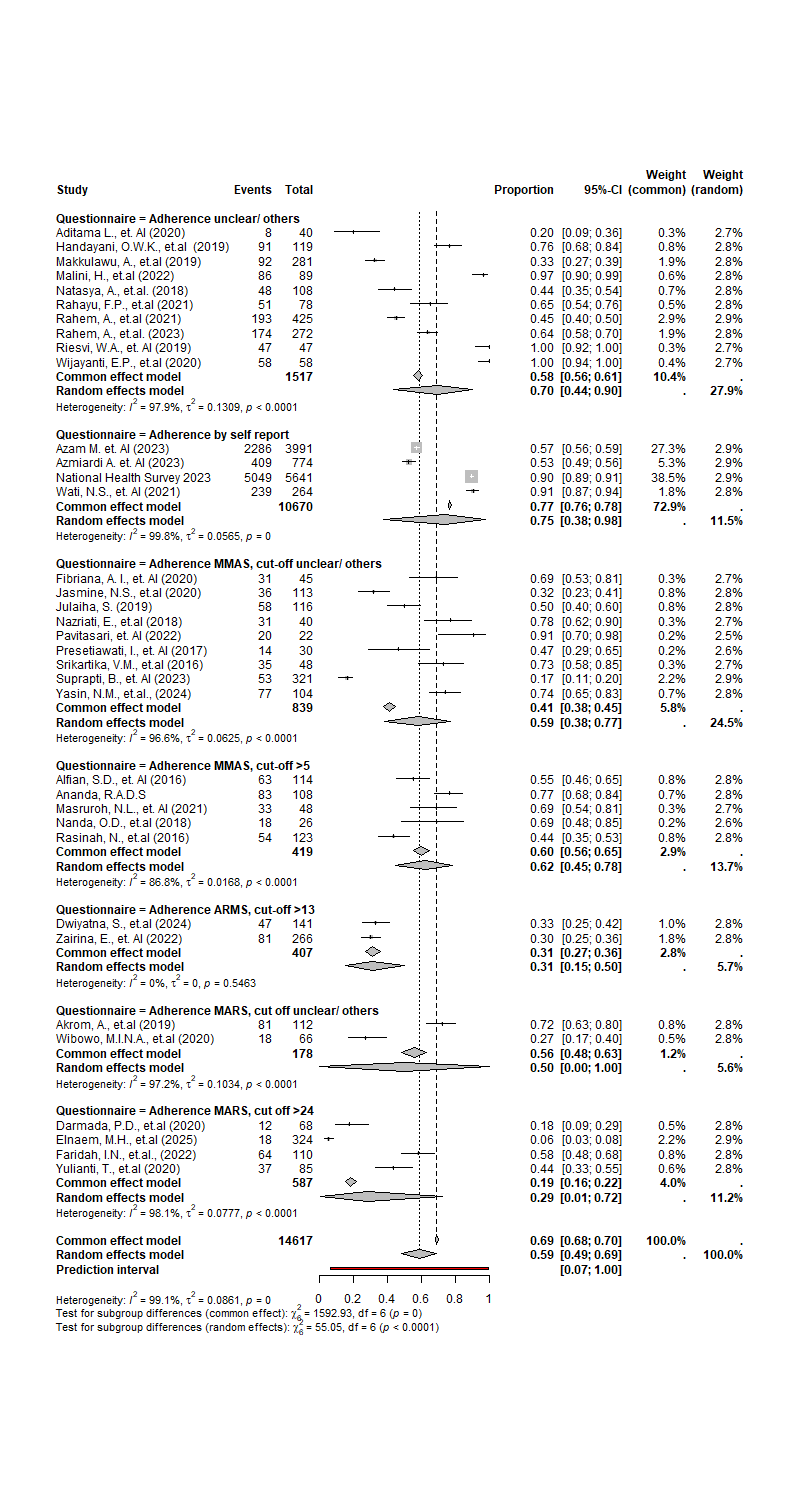


Figure E2 4 Pooled diabetes treatment adherence by measurement tools

# **Sensitivity Analysis**

Table F 1 Summary of subgroup analysis after excluding low-quality studies

| **Touchpoints** | **Subgroups** | **N. studies** | **Pooled prevalence** | **95%CI** | **I2** | **Prediction interval** |
| --- | --- | --- | --- | --- | --- | --- |
| True diabetes | Pooled | 5 | 10 | 8 – 12 | 96.6; p<0.0001 | 5 – 16 |
|  | Nationally representative studies | 4 | 10 | 7 – 14 | 97.4; p<0.0001 |  |
|  | Regionally representative studies | 0 |  |  |  |  |
|  | Provincially representative studies | 1 | 10 | 9 – 10 |  |  |
|  | General population settings | 5 | 10 | 8 – 12 | 96.6; p<0.0001 |  |
|  | Healthcare setting studies | 0 |  |  |  |  |
| Awareness | Pooled | 0 |  |  |  |  |
|  | Nationally representative studies | 0 |  |  |  |  |
|  | Regionally representative studies | 0 |  |  |  |  |
|  | Provincially representative studies | 0 |  |  |  |  |
|  | General population settings | 0 |  |  |  |  |
|  | Healthcare setting studies | 0 |  |  |  |  |
| Screening | Pooled | 4 | 11 | 0 – 41 | 100.0; p=0 | 0 – 82 |
|  | Nationally representative studies | 3 | 18 | 0 – 55 | 100.0; p=0 |  |
|  | Regionally representative studies | 1 | 1 | 0 – 1 |  |  |
|  | Provincially representative studies | 0 |  |  |  |  |
|  | General population settings | 4 | 11 | 0 – 41 | 100.0; p=0 |  |
|  | Healthcare settings | 0 |  |  |  |  |
| Diagnosis | Pooled | 4 | 4 | 0 – 11 | 99.5; p<0.0001 | 0 – 23 |
|  | Nationally representative studies | 3 | 3 | 1 – 6 | 98.1; p<0.0001 |  |
|  | Regionally representative studies | 0 |  |  |  |  |
|  | Provincially representative studies | 1 | 10 | 0 – 11 |  |  |
|  | General population settings | 4 | 4 | 0 – 11 | 99.5; p<0.0001 |  |
|  | Healthcare settings | 0 |  |  |  |  |
| Treatment | Pooled | 8 | 78 | 46 – 98 | 100.0; p=0 | 1 – 100 |
|  | Nationally representative studies | 6 | 75 | 28 – 100 | 100.0; p=0 |  |
|  | Regionally representative studies | 1 | 95 | 93 – 96 |  |  |
|  | Provincially representative studies | 1 | 78 | 74 – 81 |  |  |
|  | General population settings | 3 | 63 | 0 – 100 | 99.9; p=0 |  |
|  | Healthcare settings | 5 | 86 | 44 – 100 | 100.0; p=0 |  |
| Adherence | Pooled | 4 | 68 | 37 – 92 | 99.8; p=0 | 7 – 100 |
|  | Nationally representative studies | 3 | 68 | 14 – 100 | 99.9; p=0 |  |
|  | Regionally representative studies | 0 |  |  |  |  |
|  | Provincially representative studies | 0 |  |  |  |  |
|  | General population settings | 3 | 68 | 14 – 100 | 99.9; p=0 |  |
|  | Healthcare settings | 1 | 65 | 54 – 76 |  |  |
| Control | Pooled | 6 | 32 | 14 – 53 | 98.7; p<0.0001 | 0 – 85 |
|  | Nationally representative studies | 4 | 24 | 16 – 34 | 91.3; p<0.0001 |  |
|  | Regionally representative studies | 0 |  |  |  |  |
|  | Provincially representative studies | 2 | 50 | 0 – 100 | 98.3; p<0.0001 |  |
|  | General population settings | 2 | 19 | 8 – 33 | 0; p=0.526 |  |
|  | Healthcare settings | 4 | 39 | 10 – 73 | 99.1; p<0.0001 |  |


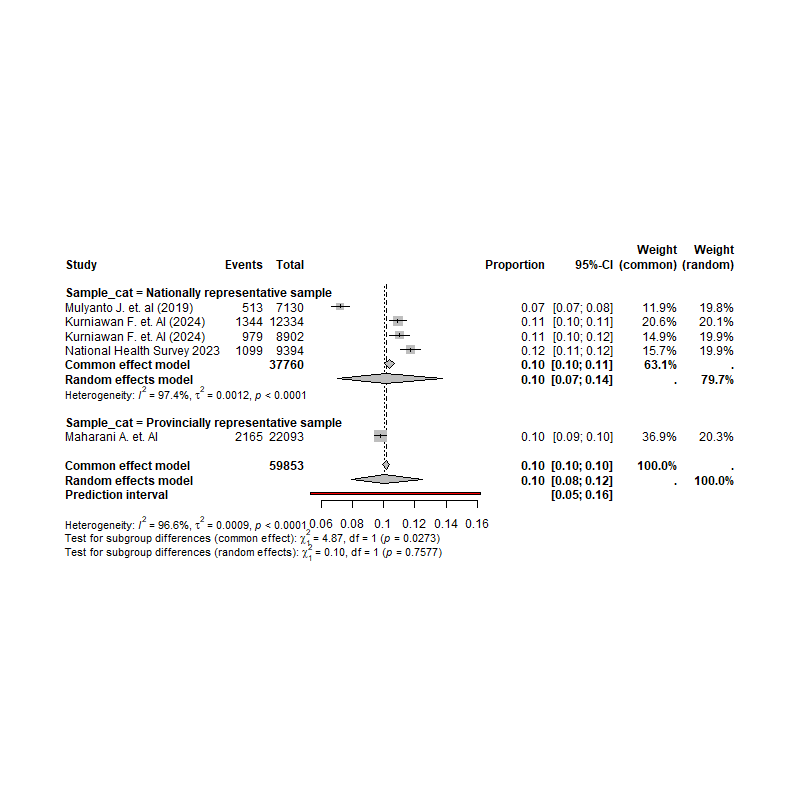


Figure F 1 Pooled diabetes prevalence by settings - sensitivity analysis


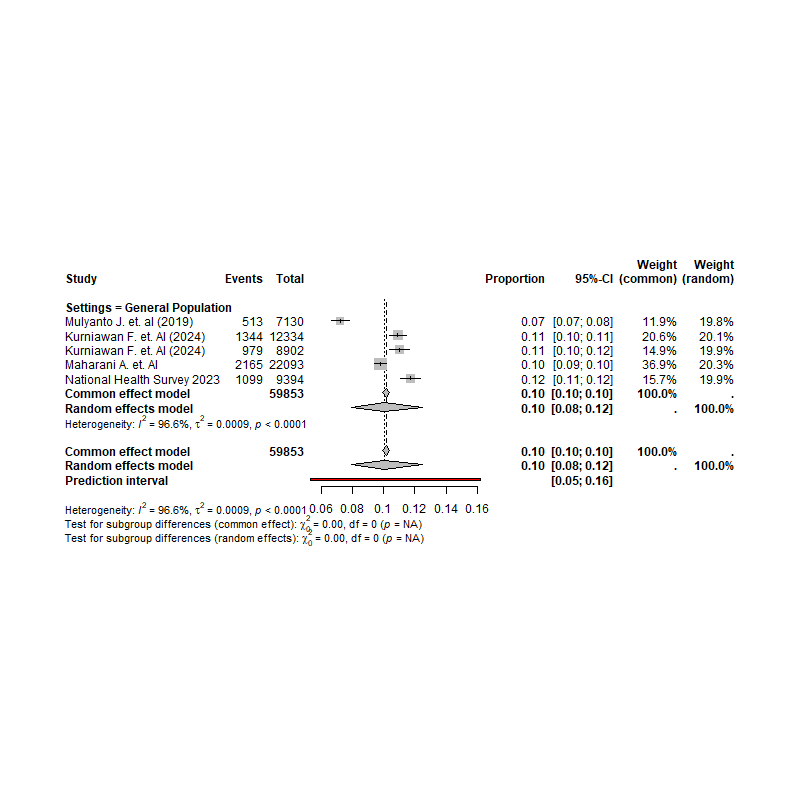


Figure F 2 Pooled diabetes prevalence by settings - sensitivity analysis


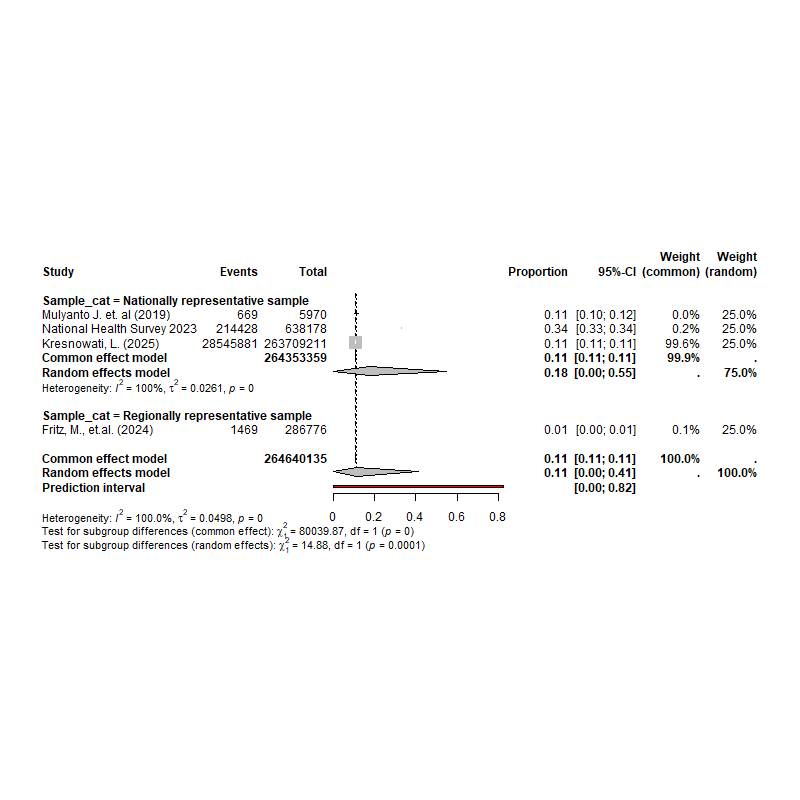


Figure F 3 Pooled diabetes screening by sample - sensitivity analysis


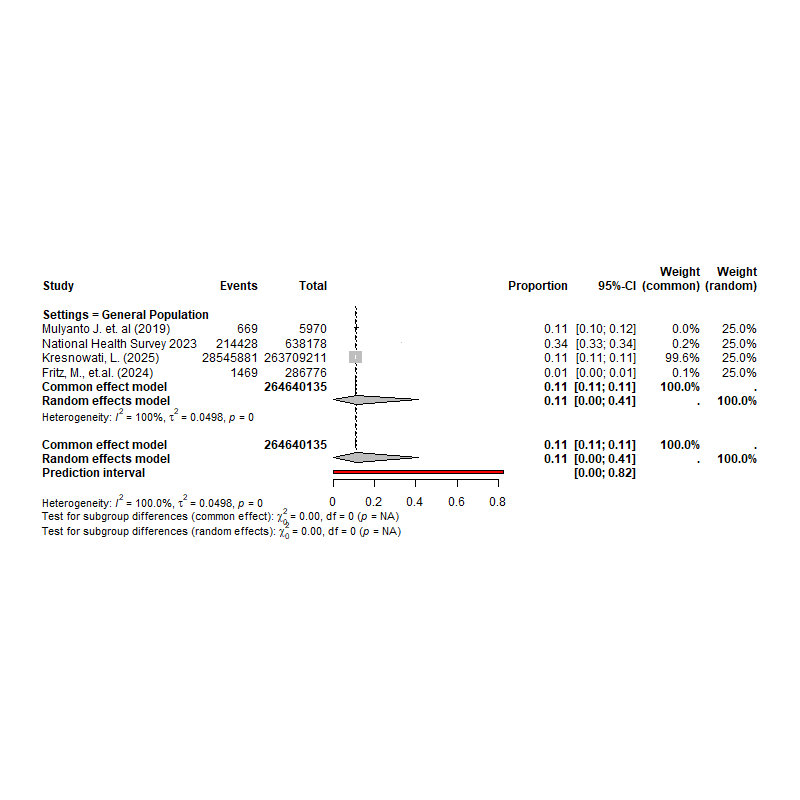


Figure F 4 Pooled diabetes screening by setting - sensitivity analysis


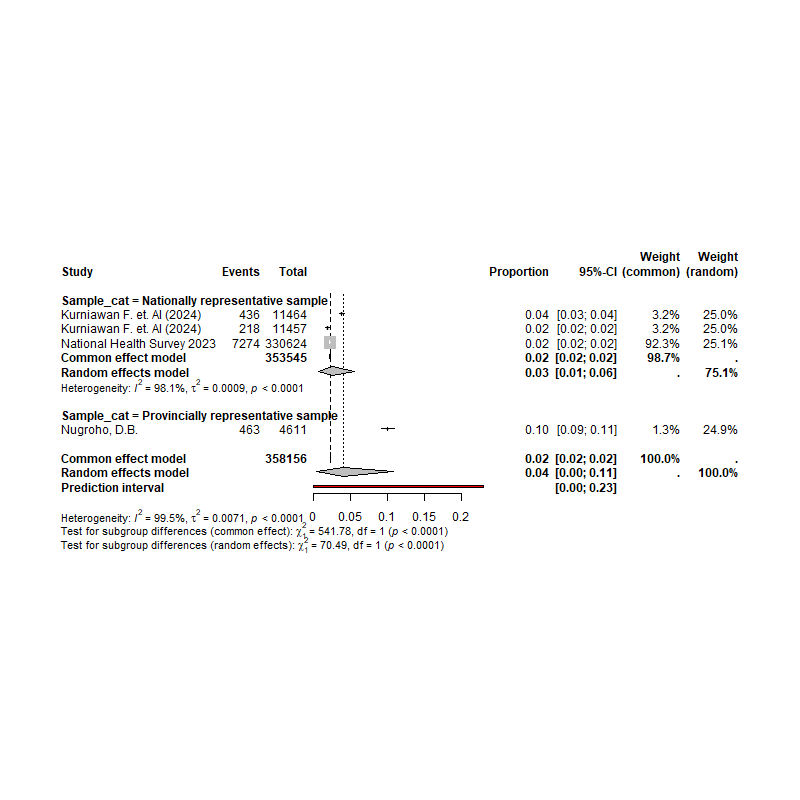


Figure F 5 Pooled diabetes diagnosis by sample - sensitivity analysis


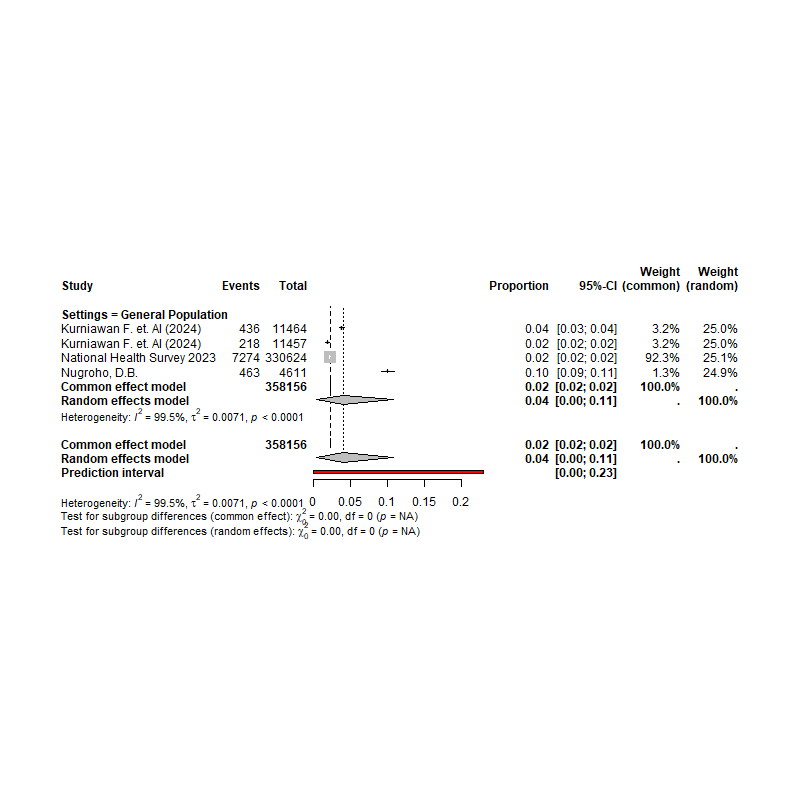


Figure F 6 Pooled diabetes diagnosis by settings - sensitivity analysis


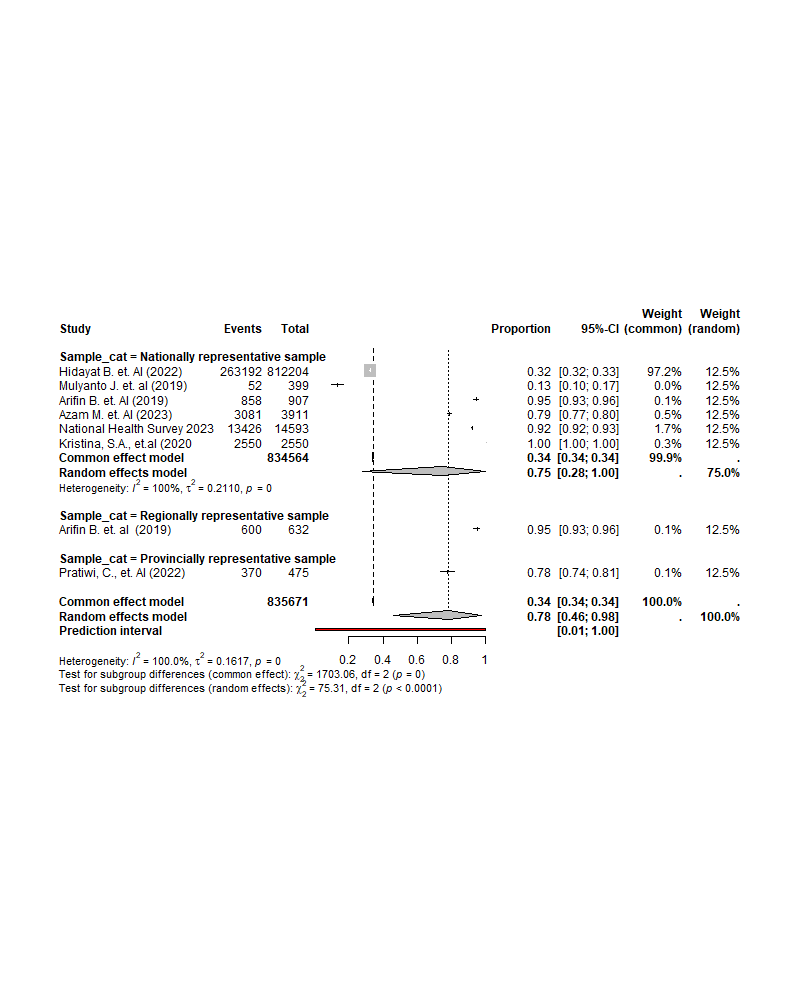


Figure F 7 Pooled diabetes treatment by sample - sensitivity analysis


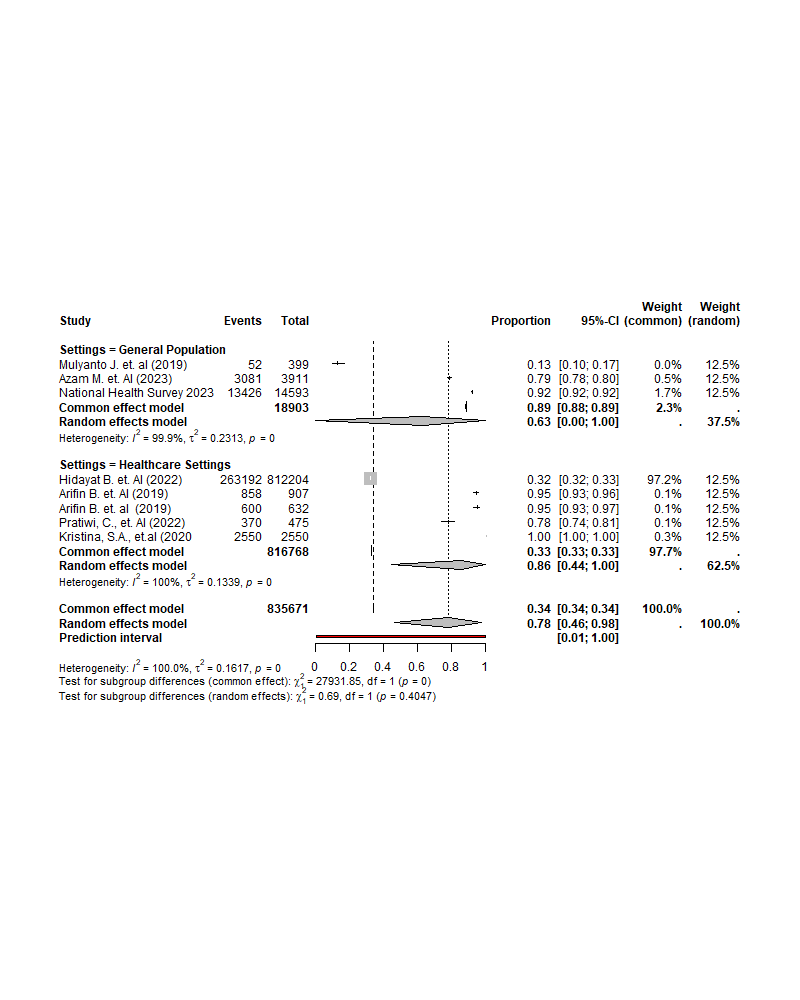


Figure F 8 Pooled diabetes treatment by settings - sensitivity analysis


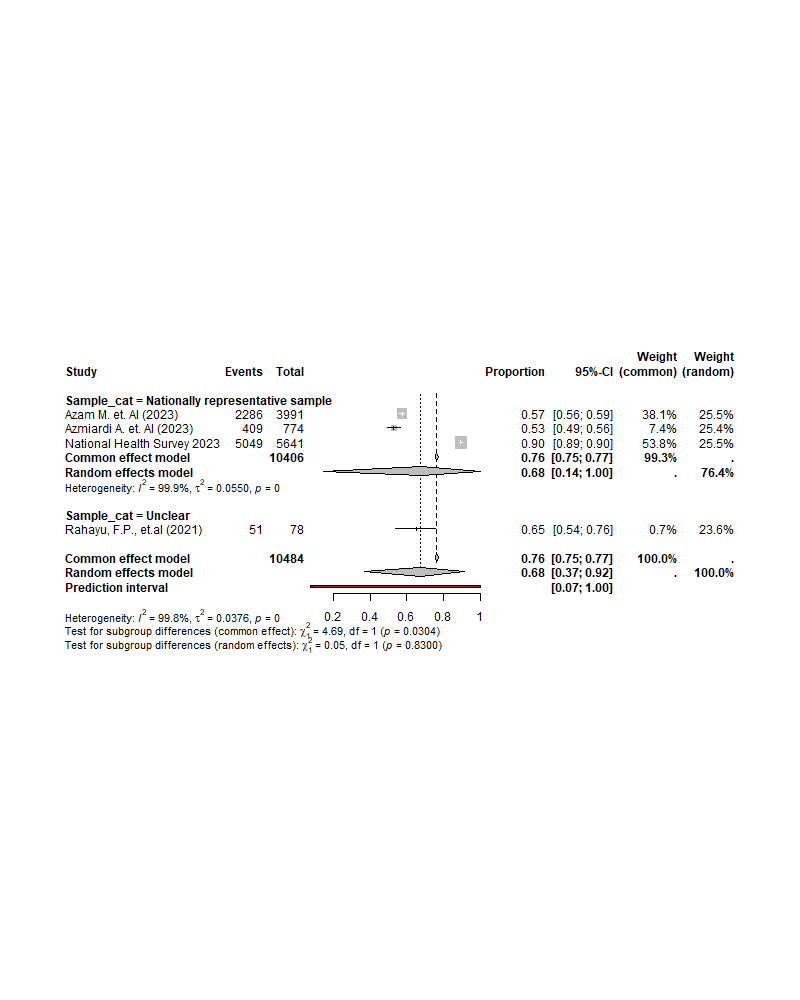


Figure F 9 Pooled diabetes treatment adherence by sample - sensitivity analysis


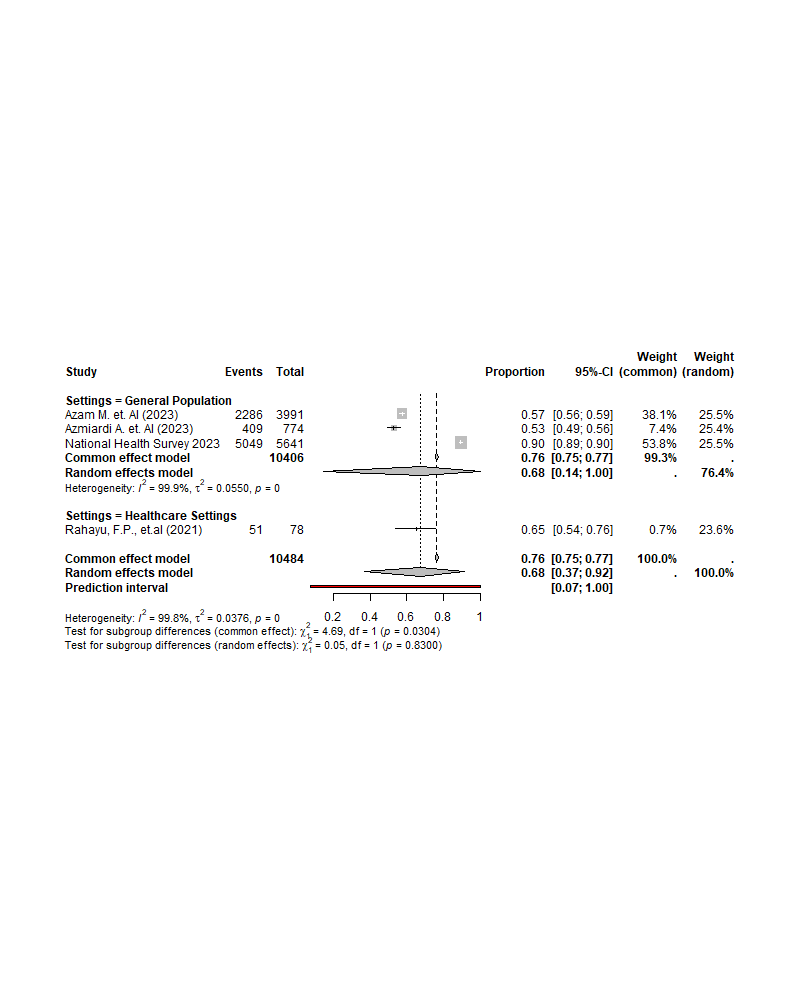


Figure F 10 Pooled diabetes treatment adherence by settings - sensitivity analysis


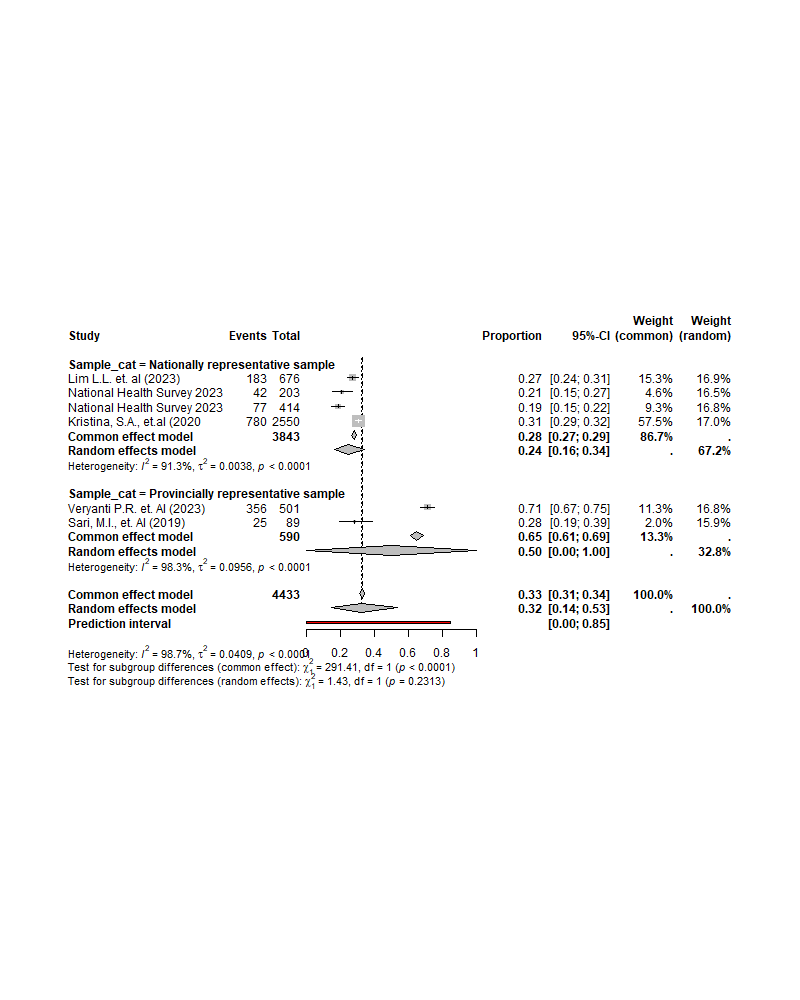


Figure F 11 Pooled diabetes control by sample - sensitivity analysis


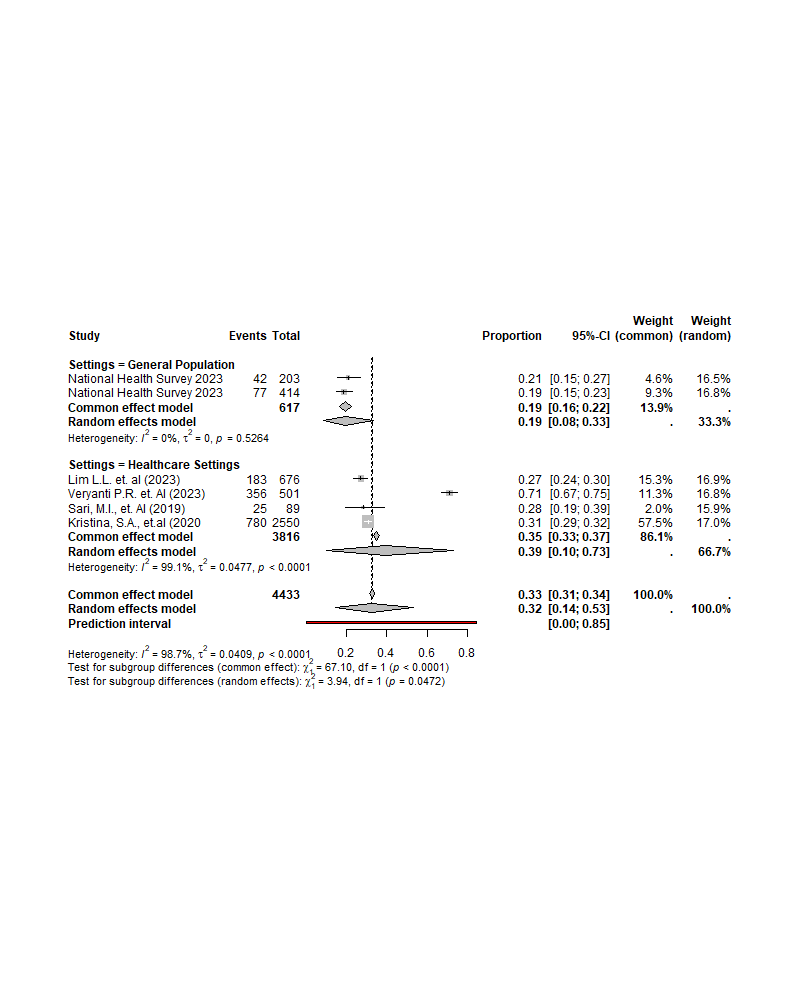


Figure F 12 Pooled diabetes control by settings - sensitivity analysis

# **References**

1. Hustrini NM, Susalit E, Rotmans JI. Prevalence and risk factors for chronic kidney disease in Indonesia: An analysis of the National Basic Health Survey 2018. J Glob Health. 2022 Oct 14;12:04074.

2. Pengpid S, Peltzer K. The prevalence of edentulism and their related factors in Indonesia, 2014/15. BMC Oral Health. 2018 Jul 3;18(1):118.

3. Hanafi AS, Sitorus N, Simbolon D, Martina M. Joint effect of high blood pressure and physical inactive on diabetes mellitus: a population-based crosssectional survey. J Prev Med Hyg. 2020 Nov 6;E614 Pages.

4. Khoiry QA, Alfian SD, Abdulah R. Sociodemographic and behavioural risk factors associated with low awareness of diabetes mellitus medication in Indonesia: Findings from the Indonesian Family Life Survey (IFLS-5). Front Public Health [Internet]. 2023;11. Available from: https://www.scopus.com/inward/record.uri?eid=2-s2.0-85147433434&doi=10.3389%2ffpubh.2023.1072085&partnerID=40&md5=349a6e235cb971ebe8d1a877e421ddea

5. Afifah AMN, Indriani D, Sebayang SK, Astutik E. RISK FACTORS FOR DIABETES MELLITUS IN INDONESIA: ANALYSIS OF IFLS DATA 2014. J Biom Dan Kependud. 2022;11(2):165–74.

6. Azam M, Sakinah LF, Kartasurya MI, Fibriana AI, Minuljo TT, Aljunid SM. Prevalence and determinants of obesity among individuals with diabetes in Indonesia [version 4; peer review: 2 approved]. 2023;

7. Arsyad DS, Westerink J, Cramer MJ, Ansar J, Wahiduddin, Visseren FLJ, et al. Modifiable risk factors in adults with and without prior cardiovascular disease: findings from the Indonesian National Basic Health Research. BMC Public Health. 2022 Apr 5;22(1):660.

8. Erlianti CP, Trihandini I. Hypertension, diabetes and cognitive impairment among elderly. Int J Public Health Sci. 2022;11(2):423–30.

9. Nugroho PS, Tianingrum NA, Sunarti S, Rachman A, Fahrurodzi DS, Amiruddin R. Predictor risk of diabetes mellitus in Indonesia, based on national health survey. Vol. 16, Malaysian Journal of Medicine and Health Sciences. 2020. p. 126–30.

10. Oktaviannoor H, Hidayat A, Hateriah S. Association between Hypertension withthe Incidence of Type 2 Diabetes Mellitus in South Kalimantan (Data Analysis of Indonesia Family Life Survey 5 Year 2014). Indian J Public Health Res Dev. 2022;13(2):210–8.

11. Zakaria SI, Alfian SD, Zakiyah N. Determinants of Cardiovascular Diseases in the Elderly Population in Indonesia: Evidence from Population-Based Indonesian Family Life Survey (IFLS). Vasc Health Risk Manag. 2022;18:905–14.

12. Adisasmito W, Amir V, Atin A, Megraini A, Kusuma D. Geographic and socioeconomic disparity in cardiovascular risk factors in Indonesia: analysis of the Basic Health Research 2018. BMC Public Health. 2020 Dec;20(1):1004.

13. Susilawati S, Wahyudi K, Jovina T, Amaliya A, Putri FM, Suwargiani AA. Indonesian Tooth Loss Predictor in Middle-aged and Elderly Populations based on Sociodemographic Factors and Systemic Disease: A Cross-sectional Study. Open Dent J. 2025 Feb 12;19(1):e18742106351915.

14. Dwi LF, Helda H. The Same Risk Factors of Hypertension in Women in Rural and Urban Areas (Following Analysis of Data Indonesia Family Life Survey 5 in 2014). Indian J Public Health Res Dev. 2020;11(3):1599–604.

15. Dilaga MS, Mat Ruzlin AN, Chen XW. Psychological distress among middle-aged adults with Diabetes Mellitus: findings from the Indonesia national population health survey (Riset Kesehatan Dasar, Riskesdas 2018). Discov Soc Sci Health. 2025 Jan 7;5(1):1.

16. Liberty IA, Kurniawan F, Wijaya CN, Soewondo P, Tahapary DL. The Impact of Lifestyle Changes on the Prevalence of Prediabetes and Diabetes in Urban and Rural Indonesia: Results from the 2013 and 2018 Indonesian Basic Health Research (RISKESDAS) Survey. Diabetology. 2024 Oct 23;5(6):537–53.

17. Merkel L, Teufel F, Malta DC, Theilmann M, Marcus ME, Flood D, et al. The Association Between Depressive Symptoms, Access to Diabetes Care, and Glycemic Control in Five Middle-Income Countries. Diabetes Care. 2024 Aug 1;47(8):1449–56.

18. Alfian SD, Sukandar H, Lestari K, Abdulah R. Medication Adherence Contributes to an Improved Quality of Life in Type 2 Diabetes Mellitus Patients: A Cross-Sectional Study. Diabetes Ther Res Treat Educ Diabetes Relat Disord. 2016 Dec;7(4):755–64.

19. Aditama L, Athiyah U, Utami W, Rahem A. Adherence behavior assessment of oral antidiabetic medication use: a study of patient decisions in long-term disease management in primary health care centers in Surabaya. J Basic Clin Physiol Pharmacol. 2020 Jan 18;30(6):/j/jbcpp.2019.30.issue-6/jbcpp-2019-0257/jbcpp-2019-0257.xml.

20. Alfian SD, Sukandar H, Arisanti N, Abdulah R. Complementary and alternative medicine use decreases adherence to prescribed medication in diabetes patients. Vol. 9, Annals of Tropical Medicine and Public Health. 2016. p. 174–9.

21. Akrom A, Sari OM, Urbayatun S, Saputri Z. Analisis determinan faktor-faktor yang berhubungan dengan kepatuhan minum obat pasien diabetes tipe 2 di pelayanan kesehatan primer. J Sains Farm Klin. 2019;6(1):54–62.

22. Amelia R, Harahap NS. Stroke among type 2 diabetes mellitus patients at Haji Adam Malik General Hospital, Medan, Indonesia. Vol. 7, Open Access Macedonian Journal of Medical Sciences. 2019. p. 2643–6.

23. Amelia R, Wahyuni AS, Yunanada Y, Fujiati II, Harahap J, Wijaya H, et al. Early Detection of Diabetic Peripheral Neuropathy in Diabetic Patients: A Cross-Sectional Study. Curr Diabetes Rev. 2024 Mar 28;

24. Amiruddin R, Jafar N, Ansar J, Limbong UB, Risnah. Motivational interviewing and physical activity on quality of life of type 2 dm patients in Makassar city. Indian J Forensic Med Toxicol. 2021;15(2):3303–13.

25. Ananda RADS, Pratiwi WR, Kristin E. Adherence and glycemic control among type 2 diabetes mellitus patients using antidiabetic medication: A cross sectional study on population registered in sleman health and demographic surveillance system. J Pharm Sci Res. 2019;11(9):3098–101.

26. Ardiany D, Pranoto A, Soelistijo SA, Libriansyah, Widjaja SA. Association between neutrophil–lymphocyte ratio on arterial stiffness in type-2 diabetes mellitus patients: a part of DiORS Study. Int J Diabetes Dev Ctries. 2022;42(2):305–12.

27. Arifin B, van Asselt ADI, Setiawan D, Atthobari J, Postma MJ, Cao Q. Diabetes distress in Indonesian patients with type 2 diabetes: a comparison between primary and tertiary care. BMC Health Serv Res. 2019;19(1):773.

28. Arifin B, Idrus LR, van Asselt ADI, Purba FD, Perwitasari DA, Thobari JA, et al. Health-related quality of life in Indonesian type 2 diabetes mellitus outpatients measured with the Bahasa version of EQ-5D. Qual Life Res. 2019;28(5):1179–90.

29. Arliny Y, Yanifitri DB, Mursalin D. High glycosylated hemoglobin level as a risk factor of latent tuberculosis infection in patients with uncomplicated type 2 diabetes mellitus. UNIVERSA Med. 2022 Apr;41(1):47–55.

30. Asril NM, Tabuchi K, Tsunematsu M, Kobayashi T, Kakehashi M. Predicting Healthy Lifestyle Behaviours Among Patients With Type 2 Diabetes in Rural Bali, Indonesia. Clin Med Insights Endocrinol Diabetes [Internet]. 2020;13((Asril N.M.; Tsunematsu M., tsunematsu@hiroshima-u.ac.jp; Kakehashi M.) Department of Health Informatics, Graduate School of Biomedical and Health Science, Hiroshima University, Hiroshima, Japan). Available from: https://www.embase.com/search/results?subaction=viewrecord&id=L2004713619&from=export

31. Azam M, Hidayati FN, Fibriana AI, Bahrudin U, Aljunid SM. Isolated Systolic Hypertension among Diabetes Mellitus Subjects; a national cross-sectional study in Indonesia. Vol. 19, Kemas. 2023. p. 53–64.

32. Bhaskara G, Budhiarta AAG, Gotera W, Saraswati MR, Dwipayana IMP, Semadi IMS, et al. Factors Associated with Diabetes-Related Distress in Type 2 Diabetes Mellitus Patients. Vol. 15, Diabetes, Metabolic Syndrome and Obesity. 2022. p. 2077–85.

33. Darmada PD, Wulandari DC. RELATION OF MEDICATION ADHERENCE TO THE INCIDENCE OF COMPLICATIONS IN TYPE 2 DIABETES MELLITUS PATIENTS. Asian J Pharm Clin Res. 2020;13(12):177–81.

34. Dewanti L, Graber MA, Pratama AP, Octora TN, Prajnaparamitha DA, Humaidy RF. Type 2 diabetes mellitus patients, profile, achievement and complications in primary health care in Surabaya, Indonesia. Popul Med. 2024;6(August):1–7.

35. Dwiyatna S, Suprapti B, Nilamsari WP, Nugroho CW, Ardiana SM. Analysis of adherence and factors affecting insulin therapy outcomes in outpatients with Diabetes Mellitus. Pharmacia. 2024;71((Dwiyatna S.; Ardiana S.M.) Clinical Pharmacy Program, Department of Pharmacy Practice, Universitas Airlangga, Surabaya, Indonesia):1–9.

36. Elnaem MH, Bukhori NAS, Tengku Mohd Kamil TK, Rahayu S, Ramatillah DL, Elrggal ME. Depression and anxiety in patients with type 2 diabetes in Indonesia and Malaysia: do age, diabetes duration, foot ulcers, and prescribed medication play a role? Psychol Health Med. 2025;30(3):555–71.

37. Faridah IN, Perwitasari DA, Maer K, Octapermatasari R, Novitasari L. Traditional Medicine and Its Impact on Patient Outcomes in Type 2 Diabetes Mellitus Therapy. Indones J Pharm. 2022;33(4):621–9.

38. Fibriana AI, Azam M, Maryuni S, Indrawati F, Windraswara R, Turnbull N. RISK FACTORS OF PULMONARY TUBERCULOSIS AMONG DIABETES MELLITUS PATIENTS: A CASE-CONTROL STUDY IN DR. KARIADI GENERAL HOSPITAL, SEAMRANG, INDONESIA. Vol. 20, Malaysian Journal of Public Health Medicine. 2020. p. 101–7.

39. Fritz M, Grimm M, My Hanh HT, Koot JAR, Nguyen GH, Nguyen TPL, et al. Effectiveness of community-based diabetes and hypertension prevention and management programmes in Indonesia and Viet Nam: a quasi-experimental study. BMJ Glob Health. 2024 May;9(5):e015053.

40. Fritz M, Grimm M, Weber I, Yom-Tov E, Praditya B. Can social media encourage diabetes self-screenings? A randomized controlled trial with Indonesian Facebook users. NPJ Digit Med. 2024 Sep 13;7(1):245.

41. Handayani OWK, Lindasari P, Rahayu SR, Yuniastuti A, Nugroho E. Path Analysis of Blood Glucose Determinant on Diabetes Mellitus Patients through Intervening Variables of Medication Adherence. Vol. 8, Unnes Journal of Public Health. 2019. p. 81–7.

42. Hendrianingtyas M, Rachmawati B, Adhipireno P. The Differences of Parathyroid Hormone, Vitamin D, and Calcium Ion Between Patients With Controlled and Uncontrolled Diabetes Mellitus. Pak J Med Health Sci. 2020 Dec;14(4):1794–7.

43. Herwana E, Febinia CA. Sirtuin, irisin, and vitamin D as predictors of diabetes mellitus with uncontrolled glycemia in Indonesian patients. Endocr Metab Sci [Internet]. 2025;17. Available from: https://www.scopus.com/inward/record.uri?eid=2-s2.0-85213252416&doi=10.1016%2fj.endmts.2024.100214&partnerID=40&md5=50b37dc3148005ebef00dc1c7bc3f31e

44. Hidayat B, Ramadani RV, Rudijanto A, Soewondo P, Suastika K, Siu Ng JY. Direct Medical Cost of Type 2 Diabetes Mellitus and Its Associated Complications in Indonesia. Value Health Reg Issues. 2022;28:82–9.

45. Indrayanti S, Anggriani Y, Andayani N. Risk factors for chronic kidney disease: a case-control study in a district hospital in Indonesia. J Pharm Sci Res. 2019;11(7):2549–54.

46. Jasmine NS, Wahyuningsih S, Thadeus MS. Analisis faktor tingkat kepatuhan minum obat pasien diabetes melitus di Puskesmas Pancoran Mas periode Maret - April 2019. J Manaj Kesehat Indones. 2020;8(1):61–6.

47. Jaya MKA, Rahmawati F, Yasin NM, Ikawati Z. Profile and factors associated with quality of life among outpatients with type 2 diabetes mellitus in Bali, Indonesia. Trop J Pharm Res. 2024;23(7):1101–9.

48. Jaya M, Rahmawati F, Yasin N, Ikawati Z. A case-control study on factors associated with hypoglycemia unawareness among the ambulatory type 2 diabetes mellitus patients in Bali, Indonesia. Trop J Pharm Res. 2025 Feb 11;24(1):109–16.

49. Julaiha S. Analisis Faktor Kepatuhan Berobat Berdasarkan Skor MMAS-8 pada Pasien Diabetes Mellitus Tipe 2. J Kesehat. 2019 Sep 13;10(2):203–14.

50. Kresnowati L, Suhartono S, Shaluhiyah Z, Widjanarko B. Drop-out to follow up screening of diabetes mellitus in Indonesia from national health insurance data 2022-2024. In 2025. Available from: https://www.scopus.com/inward/record.uri?eid=2-s2.0-85217209849&doi=10.1051%2fe3sconf%2f202560502005&partnerID=40&md5=4fc7ca7c2e5b92f6a400507403b78f65

51. Kristina SA, Endarti D, Andayani TM, Widayanti AW. Direct and indirect cost of diabetes mellitus in indonesia: A prevalence based study with human capital approach. Int J Pharm Res. 2020;13(1):2050–7.

52. Kristina SA, Salsabila FA, Suci Hanif AH. Awareness of diabetes mellitus among rural population in indonesia. Int J Pharm Res. 2021;13(1):168–75.

53. Kristanti D, Rahajeng E, Sulistiowati E, Kusumawardani N, Dany F. Determinants of diabetes comorbidities in Indonesia: a cohort study of non-communicable disease risk factor. UNIVERSA Med. 2021 Apr;40(1):3–13.

54. Kurnia AD, Amatayakul A, Karuncharernpanit S. Predictors of diabetes self-management among type 2 diabetics in Indonesia: Application theory of the health promotion model. Int J Nurs Sci. 2017 Jul 10;4(3):260–5.

55. Kurnia AD, Masruroh NL, Melizza N, Prasetyo YB, Hidayani HN. Factors Associated with Dietary Behaviour among Patients with Type 2 Diabetes Mellitus in Rural Indonesia. J ASEAN Fed Endocr Soc. 2022;37(2):1–5.

56. Kurniati I, Tjiptaningrum A, Harahap RIM, Jaya BPD. Serum Trace Element Levels in Type 2 DM Patients and its Correlation with Glycemic Control. Pharmacogn J. 2024;16(3):660–3.

57. Kurniawan F, Sigit FS, Trompet S, Yunir E, Tarigan TJE, Harbuwono DS, et al. Lifestyle and clinical risk factors in relation with the prevalence of diabetes in the Indonesian urban and rural populations: The 2018 Indonesian Basic Health Survey. Prev Med Rep. 2024 Feb;38:102629.

58. Lim LL, Lau ESH, Kong APS, Fu AWC, Lau V, Jia W, et al. Gender-associated cardiometabolic risk profiles and health behaviors in patients with type 2 diabetes: a cross-sectional analysis of the Joint Asia Diabetes Evaluation (JADE) program. Lancet Reg Health - West Pac. 2023 Mar;32:100663.

59. Maharani A, Sujarwoto, Praveen D, Oceandy D, Tampubolon G, Patel A. Cardiovascular disease risk factor prevalence and estimated 10-year cardiovascular risk scores in Indonesia: The SMARThealth Extend study [Internet]. Vol. 14, PLoS ONE. 2019. Available from: https://www.scopus.com/inward/record.uri?eid=2-s2.0-85065325224&doi=10.1371%2fjournal.pone.0215219&partnerID=40&md5=a728f9500c643eccbd96aa007631a731

60. Makkulawu A, Setiadi AP, Rahardjo TBW, Setiawan E. Analisis Profil dan Faktor-Faktor yang Memengaruhi Perilaku Kepatuhan Pengobatan untuk Pasien Diabetes Mellitus Lanjut Usia. J Kefarmasian Indones. 2019 Aug 30;114–25.

61. Malini H, Zhahara S, Lenggogeni DP, Putri ZM. SELF-CARE AND QUALITY OF LIFE PEOPLE WITH TYPE 2 DIABETES DURING THE COVID-19: CROSS-SECTIONAL STUDY. J Diabetes Metab Disord. 2022;21(1):785–90.

62. Masruroh NL, Pangastuti AF, Melizza N, Kurnia AD. Level of knowledge and family support toward medication adherence among patient with diabetes mellitus in malang, indonesia. Indian J Forensic Med Toxicol. 2021;15(1):1406–13.

63. Mayasari DS, Taufiq N, Hariawan H. Association of monocyte-to-high density lipoprotein ratio with arterial stiffness in patients with diabetes. BMC Cardiovasc Disord [Internet]. 2021;21(1). Available from: https://www.embase.com/search/results?subaction=viewrecord&id=L2013320864&from=export

64. Mulyanto J, Kringos DS, Kunst AE. Socioeconomic inequalities in the utilisation of hypertension and type 2 diabetes management services in Indonesia. Trop Med Int Health. 2019 Nov;24(11):1301–10.

65. Muhammadong J, Yuyun W, Muriman LY, Azis WA, Subhan M. Analysis of Associated Factors Contributing to Type 2 Diabetes Mellitus in the Coastal Community of Bahari Village, Southeast Sulawesi, Indonesia: A Cross-Sectional Study. Open Public Health J [Internet]. 2024;17. Available from: https://www.scopus.com/inward/record.uri?eid=2-s2.0-85196361448&doi=10.2174%2f0118749445299633240327062651&partnerID=40&md5=9d660bcf83b4ab84dcf0394b6064b2ad

66. Nanda OD, Wiryanto B, Triyono EA. Hubungan Kepatuhan Minum Obat Anti Diabetik dengan Regulasi Kadar Gula Darah pada Pasien Perempuan Diabetes Mellitus. Amerta Nutr. 2018 Dec 1;2(4):340.

67. Natasya A, Andrajati R, Sauriasari R. Cross-sectional study of association between glycemic control and quality of life among diabetic patients. Int J Appl Pharm. 2018;10(Special Issue 1):92–6.

68. Nazriati E, Pratiwi D, Restuastuti T. Pengetahuan pasien diabetes melitus tipe 2 dan hubungannya dengan kepatuhan minum obat di Puskesmas Mandau Kabupaten Bengkalis. Maj Kedokt Andalas. 2018 May 28;41(2):59.

69. Ningsih OS, Efendi F, Dewi YS. Factors associated with diabetes distress and depression in diabetes mellitus patients in rural East Nusa Tenggara Indonesia. Gac Med Caracas. 2024;132:s179–86.

70. Notariza KR, Nurcholis N, Yusaryahya H, Karimah NS, Mansur AY, Adhiguna G, et al. Gastroesophageal Reflux Disease among Elderly Type 2 Diabetes Mellitus in a Rural Area of Central Sulawesi: A Cross-sectional Study. Acta Medica Indones. 2021 Jan;53(1):42–51.

71. Nugrahaeni DK, Musri M, Inayah I, Mauliku NE. Factor Associated with Long-Term Control Blood Glucose Based on HbA1c Level in Type 2 Diabetes Mellitus Patients. Malays J Med Health Sci. 2022;18:19–23.

72. Nugroho DB, Sinorita H, Pramono B, Ikhsan R, Susanti V, Rochmah MA. Classification of Type 2 Diabetes Mellitus Using Machine Learning in Sleman District of Yogyakarta Special Region, Indonesia. Vol. 18, Malaysian Journal of Medicine and Health Sciences. 2022. p. 89–95.

73. Pratama IPY, Andayani TM, Kristina SA. Knowledge, Adherence, and Quality of Life Among Type 2 Diabetes Mellitus Patients. Int Res J Pharm. 2019;10(4):52–5.

74. Pratiwi C, Rumende M, Kshanti IAM, Soewondo P. Risk Factors for Inpatient Hypoglycemia in a Tertiary Care Hospital in Indonesia. J ASEAN Fed Endocr Soc. 2022;37(2):28–33.

75. Pavitasari A, Farapti F, Rachmah Q, Kalpana CA. Fiber Intake and Vegan Lifestyle Behaviour on Blood Glucose Control in Type 2 Diabetes Mellitus Patients: A Case-Control Study. Curr DIABETES Rev. 2023;19(5).

76. Permana H, Koesoemadinata RC, Soetedjo NNM, Dewi NF, Jayanti N, Imaculata S, et al. Diabetes mellitus patients in Indonesia: management in a tertiary hospital compared to primary health care. Universa Med. 2022 Aug 1;41(2):157–68.

77. Presetiawati I, Andrajati R, Sauriasari R. Effectiveness of a medication booklet and counseling on treatment adherence in type 2 diabetes mellitus patients. Int J Appl Pharm. 2017;9((Presetiawati I.; Andrajati R., andrajati@farmasi.ui.ac.id; Sauriasari R.) Department of Pharmaceutics, Faculty of Pharmacy, Universitas Indonesia, Depok, Indonesia):27–31.

78. Putra GM, Kawiyana KS, Wiratnaya GE, Suyasa K. Duration of Type 2 Diabetes Mellitus Over 5 Years, HbA1c Levels Over 7%, Alkaline Phospatase Over 130 IU/L, and C-Reactive Protein Over 3 mg/dL as Risk Factors for Osteoporosis in Type 2 DM Patients. Open Access Maced J Med Sci. 2023;11(B):714–9.

79. Rahayu FP, Wicaksana AL, Haryani. Medication, illness duration, and medication adherence among peer support groups of diabetic patients. Int J Pharm Res. 2021;13(3):582–9.

80. Rahem A, Athiyah U, Setiawan CD. The influence of participation of healthcare insurance and social security (BPJS) on therapeutic success in diabetes mellitus patients at primary healthcare centers in Madura. Trop J Nat Prod Res. 2021;5(1):71–6.

81. Rahem A, Athiyah U, Setiawan CD, Hermansyah A. The risk of combined use of herbal and conventional medicines in diabetic patients. Pharm Educ. 2023;23(4):185–8.

82. Universitas Negeri Gorontalo, Gorontalo, Indonesia, Rasdianah N, Martodiharjo S, Pascasarjana Fakultas Farmasi, Universitas Gadjah Mada, Yogyakarta, Indonesia, Andayani TM, Pascasarjana Fakultas Farmasi, Universitas Gadjah Mada, Yogyakarta, Indonesia, et al. The Description of Medication Adherence for Patients of Diabetes Mellitus Type 2 in Public Health Center Yogyakarta. Indones J Clin Pharm. 2016 Dec 1;5(4):249–57.

83. Romadlon DS, Huang HC, Chen YC, Hu SH, Hasan F, Chiang Morales MD, et al. Fatigue following type 2 diabetes: Psychometric testing of the Indonesian version of the multidimensional fatigue Inventory-20 and unmet fatigue-related needs. PloS One. 2022;17(11):e0278165.

84. Romadlon DS, Huang HC, Chen YC, Hu SH, Kurniawan R, Tarigan TJE, et al. Effects of Personalized DiaBetes TEXT Messaging Combined with Peer Support Education on Patients With Type 2 Diabetes: A Randomized Controlled Trial. J Diabetes Sci Technol. 2025 Jan 29;19322968251314501.

85. Rosaria E, Nurahmi, Kurniawan LB. Correlation of glycated haemoglobin with netrin-1 and high sensitive c-reactive proteinin type 2 diabetes melitus patients. Medico-Leg Update. 2020;20(4):1113–7.

86. Riesvi WA, Pristianty L, Faturrochmah A. Behavioral factors affecting patient’s compliance in consuming anti-diabetic oral drugs. J Glob Pharma Technol. 2019;11(4):147–52.

87. Risdahidayanti F, Prabadiyan R, Abu Bakar S. Analysis of risk factors related to coroner heart disease in one of the Indonesian national hospitals. Vol. 24, International Journal of Psychosocial Rehabilitation. 2020. p. 4089–97.

88. Santosa WRB, Nambiar N, Abdullah E. Uncovering the Multifaceted Influences on Type-2 Diabetes Mellitus Incidence in Public Health Centre, Indonesia. Malays J Nurs. 2024;16(1):80–8.

89. Sari MI, Tala ZZ, Wahyuni DD. Association between glycated hemoglobin with the levels of serum proinflammatory cytokines and antioxidants in patients with type 2 diabetes mellitus in universitas sumatera utara hospital. Open Access Maced J Med Sci. 2019;7(5):715–20.

90. Sauriasari R, Sakti RM. IMPACT OF A PHARMACIST-LED PATIENT EDUCATION INITIATIVE ON GLYCEMIC CONTROL OF PATIENTS WITH TYPE 2 DIABETES MELLITUS: A SINGLE-CENTER EXPERIENCE IN WEST JAKARTA, INDONESIA. Int J Appl Pharm. 2018 Dec 20;10(1):252.

91. Siregar F, Asfriyati A, Makmur T, Bestari R, Lubis I, Zein U. Identifying Adult Population at Risk for Undiagnosed Diabetes Mellitus in Medan City, Indonesia Targeted on Diabetes Prevention. Med Arch. 2023;77(6):455.

92. Srikartika alentina M, Cahya AD, Hardiati RSW. Analisis faktor yang memengaruhi kepatuhan penggunaan obat pasien diabetes melitus tipe 2. J Manaj Dan Pelayanan Kesehat. 2016;6(3).

93. Subekti I, Pramono LA, Dewiasty E, Harbuwono DS. Thyroid Dysfunction in Type 2 Diabetes Mellitus Patients. Acta Medica Indones. 2017 Oct;49(4):314–23.

94. Suprapti B, Izzah Z, Anjani AG, Andarsari MR, Nilamsari WP, Nugroho CW. Prevalence of medication adherence and glycemic control among patients with type 2 diabetes and influencing factors: A cross-sectional study. Glob Epidemiol [Internet]. 2023;5. Available from: https://www.scopus.com/inward/record.uri?eid=2-s2.0-85161645989&doi=10.1016%2fj.gloepi.2023.100113&partnerID=40&md5=c9aa9f9712cfc62cd0f7b393592a06b1

95. Tarigan M, Megawati ER. Prediabetes, undiagnosed diabetes, and associated factors in North Sumatra, Indonesia: A community-based study. Romanian J Diabetes Nutr Metab Dis. 2024;31(4):420–7.

96. Turana Y, Lipnicki DM, Handajani YS, Sani TP, Widayanti JR, Suswanti I, et al. Factors associated with odour identification in older Indonesian and white Australian adults. Aging Clin Exp Res. 2020 Feb;32(2):215–21.

97. Ulfah NH, Katmawanti S, Sukma AM, Rahmawati IT, Wongsasuluk P, Alma LR, et al. Personal characteristics, families, and community support associated with self-care behavior among Indonesian diabetic patients. J Public Health Afr [Internet]. 2022;13(s2). Available from: https://www.embase.com/search/results?subaction=viewrecord&id=L2018789721&from=export

98. Veryanti PR, Sauriasari R, Sartika RAD, Elya B. Factors Influencing Hypoglycemia in Type 2 Diabetes Mellitus Outpatients with State Health Insurance at Regional General Hospitals in Jakarta, Indonesia. Curr Diabetes Rev. 2024 Jan 11;

99. Wati NS, Wongsasuluk P, Soewondo P. A cross-sectional study on the telemedicine usage and glycemic status of diabetic patients during the COVID-19 pandemic. Med J Indones. 2021;30(3):215–20.

100. Wibowo MINA, Setiawan D, Ikhwaniati ND, Sukma FA. Pengaruh konseling dan alat bantu pengingat pengobatan terhadap kepatuhan minum obat dan outcome klinik pasien diabetes melitus dan hipertensi. J Ilmu Kefarmasian Indones. 18(2):169–76.

101. Wijayanti EP, Pristianty L, Faturrohmah A. Oral antidiabetic drug consumption adherence in primary health care through PRECEDE method. Res J Pharm Technol. 2020;13(2):543–6.

102. Wulandari N, Maifitrianti M, Hasanah F, Atika S, Putri RD. Medication adherence assessment among patients with type 2 diabetes mellitus treated polytherapy in indonesian community health center: A cross sectional-study. J Pharm Bioallied Sci. 2020;12(6):S758–62.

103. Wulan W, Widianawati E, Pantiawati I, Wulandari F. Telemedicine Homecare Among the Hypertension and Diabetes Mellitus Risk Elderly Group in Indonesian Primary Healthcare: A Technology Acceptance Model. HOME Health CARE Manag Pract. 2024 May;36(2):88–94.

104. Wungu CDK, Elvaretta C, Palupi DER, Qurnianingsih E, Susilo H, Lukitasari L, et al. RISK FACTOR PROFILE AND ROLE OF CARDIOVASCULAR DISEASE OUTREACH PROGRAM BY EXPERTS IN RURAL COMMUNITIES: A PILOT STUDY IN MAGETAN REGENCY, INDONESIA. J Keperawatan Soedirman. 2024;19(2):116–27.

105. Yasin NM, Ekasari MP, Prabasworo W, Arom S, Ridhayani F. Knowledge Level and Information Needs of Patients with Diabetes in Yogyakarta City, Indonesia. Health Educ Health Promot. 2024;12(3):431–8.

106. Yasin NM, Kurniawati F, Ridhayani F, Salsabila FA. Diabetes-related patients’ profile and strategy in improving their knowledge and adherence: A mixed method study. Pharm Educ. 2024 Nov 23;24(1):678–87.

107. Yulianti T, Anggraini L. Faktor-Faktor yang Mempengaruhi Kepatuhan Pengobatan pada Pasien Diabetes Mellitus Rawat Jalan di RSUD Sukoharjo. Pharmacon J Farm Indones. 2020 Dec 31;17(2):110–20.

108. Yunir E, Nugraha ARA, Rosana M, Kurniawan J, Iswati E, Sarumpaet A, et al. Risk factors of severe hypoglycemia among patients with type 2 diabetes mellitus in outpatient clinic of tertiary hospital in Indonesia. Sci Rep. 2023 Sep;13(1).

109. Zairina E, Nugraheni G, Sulistyarini A, Mufarrihah, Setiawan CD, Kripalani S, et al. Factors related to barriers and medication adherence in patients with type 2 diabetes mellitus: a cross-sectional study. J Diabetes Metab Disord. 2022;21(1):219–28.

110. Badan Kebijakan Pembangunan Kesehatan M of H of R of I. Survei Kesehatan Indonesia Dalam Angka (Indonesian Health Survey in Number) 2023 [Internet]. Jakarta, Indonesia; 2023 [cited 2024 Jun 11]. Available from: https://layanandata.kemkes.go.id/katalog-data/ski/ketersediaan-data/ski-2023

111. Munn Z, Moola S, Lisy K, Riitano D, Tufanaru C. Methodological guidance for systematic reviews of observational epidemiological studies reporting prevalence and cumulative incidence data. Int J Evid Based Healthc. 2015 Sep;13(3):147–53.
